# Supplementary material for: Effectiveness of postdischarge interventions for reducing the severity of chronic pain after total knee replacement: systematic review of randomised controlled trials
Source: BMJ Open. 2018 Feb 28;8(2):e020368. doi: 10.1136/bmjopen-2017-020368 (PMC5855247; doi:10.1136/bmjopen-2017-020368)
Supplement: Supplementary file 2 [file bmjopen-2017-020368supp002.pdf]

Appendix 2: Search terms

MEDLINE (Ovid) (1946 to 15 November 2016)

1 randomized controlled trial/ or randomized controlled trial.pt.

2 controlled clinical trial.pt.

3 randomized.ab.

4 placebo.ab.

5 randomly.ab

6 trial.ab

7 randomised.tw

8 1 or 2 or 3 or 4 or 5 or 6 or 7

9 review/

10 'systematic review$'.mp

11 9 or 10

12 8 or 11

13 Arthroplasty, Replacement, Knee/

14 Knee Prosthesis/

15 (arthoplast$ adj3 knee$).mp. [mp=title, abstract, original title, name of substance word, subject heading word, keyword heading word, protocol supplementary concept word, rare disease supplementary concept word, unique identifier]

16 (knee$ adj3 replac$).mp. [mp=title, abstract, original title, name of substance word, subject heading word, keyword heading word, protocol supplementary concept word, rare disease supplementary concept word, unique identifier]

17 (knee adj3 implant$).mp. [mp=title, abstract, original title, name of substance word, subject heading word, keyword heading word, protocol supplementary concept word, rare disease supplementary concept word, unique identifier]

18 13 or 14 or 15 or 16 or 17

19 12 and 18

EMBASE (Ovid) (1980 to 15 November 2016)

1 Randomized controlled trial/ or Randomization/ or Single blind procedure/ or Double blind procedure/ or Crossover procedure/ or Placebo/ or Randomised controlled trial$.tw. or Randomized controlled trial$.tw. or RCT.tw. or Random allocation.tw. or Randomly allocated.tw. or Allocated randomly.tw. or (allocated adj2 random).tw. or Single blind$.tw. or Double blind$.tw. or ((treble or triple) adj blind$).tw. or Placebo$.tw.

2 "systematic review"/

3 meta analysis/

4 2 or 3

5 1 or 4

6 knee arthroplasty/

7 total knee arthroplasty/

8 knee prosthesis/

9 (knee$ adj3 arthoplast$).mp. [mp=title, abstract, heading word, drug trade name, original title, device manufacturer, drug manufacturer, device trade name, keyword, floating subheading]

10 (knee$ adj3 replac$).mp. [mp=title, abstract, heading word, drug trade name, original title, device manufacturer, drug manufacturer, device trade name, keyword, floating subheading]

11 (knee$ adj3 implant$).mp. [mp=title, abstract, heading word, drug trade name, original title, device manufacturer, drug manufacturer, device trade name, keyword, floating subheading]

12 6 or 7 or 8 or 9 or 10 or 11

13 5 and 12

PsycINFO (Ovid) (inception [1806] to 15 November 2016)

1. (knee$ adj3 arthoplast$).mp. [mp=title, abstract, heading word, table of contents, key concepts, original title, tests & measures]

2. (knee$ adj3 replac$).mp. [mp=title, abstract, heading word, table of contents, key concepts, original title, tests & measures]

3. (knee$ adj3 surg$).mp. [mp=title, abstract, heading word, table of contents, key concepts, original title, tests & measures]

4. (knee$ adj3 implant$).mp. [mp=title, abstract, heading word, table of contents, key concepts, original title, tests & measures]

5. (knee adj3 prosthe$).mp. [mp=title, abstract, heading word, table of contents, key concepts, original title, tests & measures]

6. 1 or 2 or 3 or 4 or 5

The Cochrane Library (Wiley) (inception to 15 November 2016)

#1 MeSH descriptor: [Knee Prosthesis] explode all trees

#2 MeSH descriptor: [Arthroplasty, Replacement, Knee] explode all trees

#3 arthoplast* N3 knee*

#4 knee* N3 replac*

#5 knee* N3 implant*

#6 #1 or #2 or #3 or #4 or #5

CINAHL (EBSCOHOST) (1982 to 15 November 2016)

S25	S15 AND S23 Limiters - Exclude MEDLINE records
S24	S15 AND S23
S23	S16 OR S17 OR S18 OR S19 OR S20 OR S21 OR S22
S22	knee* N3 implant*
S21	knee* N3 arthoplast*
S20	arthoplast* N3 knee*
S19	knee* N3 replac*
S18	"knee prosthes*"
S17	MH "Knee surgery"
S16	MH "Arthroplasty, Replacement, Knee"
S15	(S8 OR S14)
S14	(S9 OR S10 OR S11 OR S12 OR S13)
S13	metaanalyses
S12	metaanalysis
S11	meta-analyses
S10	meta-analysis
S9	systematic review
S8	S1 OR S2 OR S3 OR S4 OR S5 OR S6 OR S7
S7	AB trial$
S6	AB randomly
S5	AB randomised OR randomized
S4	(MH "clinical trials")
S3	clinical trials
S2	(MH "randomized controlled trials")
S1	randomized controlled trials
