# Supplementary material for: Effectiveness of postdischarge interventions for reducing the severity of chronic pain after total knee replacement: systematic review of randomised controlled trials
Source: BMJ Open. 2018 Feb 28;8(2):e020368. doi: 10.1136/bmjopen-2017-020368 (PMC5855247; doi:10.1136/bmjopen-2017-020368)
Supplement: Supplementary file 3 [file bmjopen-2017-020368supp003.pdf]

Appendix 3: Trial results summary

Study	Primary aim of the study	Primary outcome (and measure)	Results summary
Bruun-Olsen et al, 2013	To examine the immediate and long-term effects of a walking-skill program compared with usual physiotherapy care.	Walking distance (6 minute walk test)	No significant difference in mean 0-100 KOOS pain scores (p-value not reported):<br>Intervention: 82 (SD 21)<br>Control: 74 (SD 23)<br><br>Mean change from baseline (6 weeks post-operative):<br>Intervention: 21<br>Control: 20
Buhagiar et al, 2017	To determine if 10 days of inpatient rehabilitation followed by a monitored home-based program provided greater improvements than a monitored home-based program alone.	Walking distance (6 minute walk test)	No significant difference in median 0-100 KOOS pain scores between groups (p-value not reported):<br>Intervention: 86 (IQR 74 to 97)<br>Control: 91 (IQR 78-98)<br><br>Mean change from baseline (pre-surgery):<br>Intervention: 53<br>Control: 55
Buker et al, 2014	Determine the functional differences between patients who were treated with supervised physiotherapy or a standardized home program and perform a cost analysis.	Not specified	No significant difference in mean pain 0-10 VAS scores (p-value not reported):<br>Rest pain - Intervention: 0.44 (SD 0.51); Control: 0.37 (SD 0.80)<br>Activity pain - Intervention: 3.11 (SD 1.96); Control: 2.50 (SD 1.77)<br><br>Mean change from baseline (pre-surgery):<br>Rest pain - Intervention: 4.86; Control: 4.78<br>Activity pain - Intervention: 6.14; Control: 5.46
Chen et al, 2016	To assess the impact of structured telephone reinforcement on patient	Not specified (pilot study)	No significant difference in mean 0-100 VAS pain scores (p-value not reported):

Appendix 3: Trial results summary

Study	Primary aim of the study	Primary outcome (and measure)	Results summary
	compliance with home exercises.		Intervention: 8.7 (SD 5.1)<br>Control: 9.3 (SD 5.5)<br><br>Mean change from baseline (pre-surgery):<br>Intervention: 63.5<br>Control: 63.8
Fransen et al, 2017	To evaluate the long-term benefit of providing a post-acute, class-based outpatient exercise program compared with current usual rehabilitation care.	Pain and function (WOMAC pain and function scales)	No significant difference in mean 0-20 WOMAC pain scores (p=0.71):<br>Intervention: 2.6 (SE 0.2)<br>Control: 2.5 (SE 0.2)<br><br>Mean change from baseline (pre-surgery):<br>Intervention: 8.7<br>Control: 8.5
Frost et al, 2002	To assess the feasibility of comparing traditional exercise regimes with a more functional and dynamic approach.	Not specified (feasibility study)	No significant difference in mean 1-5 OKS item 'pain on walking' scores (p=0.68):<br>Intervention: 1.6 (SD 0.8)<br>Control: 1.5 (SD 0.93)<br><br>Mean change from baseline (pre-surgery):<br>Intervention: 2.6<br>Control: 2.8
Kauppila et al, 2010	To examine whether a multidisciplinary rehabilitation programme can improve functional recovery and quality of life and reduce the use of rehabilitation services compared with conventional care.	Function (WOMAC function scale)	No significant difference in mean 0-100 WOMAC pain scores (p=0.17):<br>Intervention: 23.5 (SD 22.3)<br>Control: 19.3 (SD 17.5)<br><br>Mean change from baseline (pre-surgery):

Appendix 3: Trial results summary

Study	Primary aim of the study	Primary outcome (and measure)	Results summary
			Intervention: 39.3<br>Control: 37.1
Ko et al, 2013	To determine whether center-based, one-to-one physical therapy provides superior outcomes compared with group-based therapy or a simple monitored home-based program.	Pain and function (Oxford Knee Score)	No significant difference in mean 0-50 WOMAC pain scores (p=0.79):<br>1:1 sessions: Median 3.8 (IQR 0.5-9.6)<br>Group sessions: Median 1.6 (IQR 0-7.5)<br>Home programme: Median 2.5 (IQR 0-9.5)<br><br>Mean change from baseline (pre-surgery):<br>1:1 sessions: 25.65<br>Group sessions: 18.4<br>Home programme: 25.7
Kramer et al, 2003	To compare clinic-based rehabilitation delivered in outpatient physical therapy clinics and home-based rehabilitation monitored by a physical therapist via periodic telephone calls.	Not specified	No significant difference in mean WOMAC pain scores (p-value and mean pain scores not reported)
Liebs et al, 2010	Evaluate the effect of ergometer cycling on health-related quality of life and patient satisfaction.	Function (WOMAC function scale)	No significant difference in mean 0-100 WOMAC pain scores (p=0.454):<br>Intervention: 15.6 (SD 17.9)<br>Control: 13.0 (SD 14.9)<br><br>Mean change from baseline (pre-surgery):<br>Intervention: 38.8<br>Control: 41.1
Liebs et al, 2012	To evaluate if the timing of aquatic therapy influences clinical outcomes.	Function (WOMAC function scale)	No significant difference in mean 0-100 WOMAC pain scores (p=0.334):<br>Intervention: 13.2 (SD 15.0)<br>Control: 17.4 (SD 22.4)

Appendix 3: Trial results summary

Study	Primary aim of the study	Primary outcome (and measure)	Results summary
			Mean change from baseline (pre-surgery):<br>Intervention: 39.9<br>Control: 32.8
Minns Lowe et al, 2012	To evaluate a pilot trial of a postdischarge physiotherapy intervention to improve patient function versus usual physiotherapy.	Pain and function (Oxford Knee Score)	No statistical comparison of median 0-100 KOOS pain scores (pilot study):<br>Intervention: 80.6 (IQ 36)<br>Control: 90.3 (IQ 33)<br><br>Mean change from baseline (pre-surgery):<br>Intervention: 39.5<br>Control: 51.4
Moffet et al, 2004	To evaluate the effectiveness of a new intensive functional rehabilitation program on functional ability and quality of life.	Walking distance (6 minute walk test)	No significant difference in mean 0-100 WOMAC pain scores (p=0.161):<br>Intervention: 9.4 (SD 12.4)<br>Control: 11.8 (SD 13.0)<br><br>Mean change from baseline (2 months post-operative):<br>Intervention: 19<br>Control: 10.8
Monticone et al, 2013	To compare the improvement in disability, kinesiophobia, pain, and quality of life obtained by means of home-based functional exercises aimed at managing kinesiophobia with advice to stay active after discharge from a rehabilitation unit.	Pain and function (KOOS)	Mean 0-100 KOOS pain score significantly lower in intervention group (p<0.001):<br>Intervention: 87.35 (SD 11.71)<br>Control: 77.38 (SD 15.07)<br><br>Mean change from baseline (before discharge from the rehabilitation unit):<br>Intervention: 41.95<br>Control: 34.64
Petterson et al, 2009	To determine the effectiveness of progressive quadriceps strengthening	Quadriceps strength and volitional	No significant difference in mean 0-5 KOS ADL item 'affect of pain on function' (p value not reported):

Appendix 3: Trial results summary

Study	Primary aim of the study	Primary outcome (and measure)	Results summary
	with or without neuromuscular electrical stimulation (NMES).	muscle activation (burst superimposition technique)	Intervention: 0.82 (SD not reported)<br>Control: 0.89 (SD not reported)<br><br>Mean change from baseline (3-4 weeks post-operative):<br>Intervention: 1.42<br>Control: 1.55
Szots et al, 2016	To evaluate the effects of structured nurse-managed telephone follow-up.	Function (WOMAC function scale)	No significant difference in mean change in 0-100 WOMAC pain scores from baseline (3 days post hospital discharge) (p=0.329):<br>Intervention: -25.9 (95% CI = -30.8, -21.0)<br>Control: -29.5 (95% CI = -35.2, -23.8)
Vuorenmaa et al, 2014	To evaluate the efficacy of a delayed home exercise programme compared with normal care.	Pain (WOMAC pain scale)	No significant difference in mean change in 0-100 WOMAC pain scores from baseline (2 months post-operative) (p=0.70):<br>Intervention: -15 (95% CI -20 to -10)<br>Control: -14 (95% CI -19 to -9)
