# Supplementary material for: Effectiveness of postdischarge interventions for reducing the severity of chronic pain after total knee replacement: systematic review of randomised controlled trials
Source: BMJ Open. 2018 Feb 28;8(2):e020368. doi: 10.1136/bmjopen-2017-020368 (PMC5855247; doi:10.1136/bmjopen-2017-020368)
Supplement: Supplementary file 4 [file bmjopen-2017-020368supp004.pdf]

Appendix 4: Adverse event reporting and findings

Publication	Adverse events assessment/definition	Results summary
Buker et al, 2014	None	
Bruun-Olsen et al, 2013	None	
Buhagiar et al, 2017	Postdischarge complication and adverse event data were collected until 1 year after surgery by self-report at follow-up visits and by a review of hospital electronic medical records.	Detailed breakdown of post-operative complications is provided in Table 3. There were no significant between-group differences in emergency department visits, readmissions and manipulations under anaesthetic.
Chen et al, 2016	None	
Fransen et al, 2017	Adverse events defined as event resulting in readmission to the hospital or resulting in a medical intervention or reduced function for 3 or more days.	Intervention: 1 death, 24 hospital admissions (15 TKR related), 20 other adverse events<br><br>Control: 1 death, 16 hospital admissions (13 TKR related), 17 other adverse events
Frost et al, 2002	None	
Kauppila et al, 2010	Not stated	No adverse events due to the intervention were reported
Ko et al, 2013	Postoperative adverse events were monitored via treating therapists and documented by the blinded assessor.	Detailed breakdown of post-operative adverse events provided in Appendix 1. Superficial wound infection, major infection, venous thrombotic embolism, neurovascular, TKR-related readmission and manipulation under anaesthetic similar between groups. No adverse events were associated with any of the treatment arms.
Kramer et al, 2003	None	
Liebs et al, 2010	Not stated	Prevalence of postoperative complications was similar between groups.
Liebs et al, 2012	Not stated	5 patients in early aquatic therapy group and 1 patient in the late aquatic therapy group admitted to hospital within 3 months of surgery.
Minns Lowe et al, 2012	Not stated	No adverse events
Moffet et al, 2004	Not stated	No adverse events
Monticone et al, 2013	None	
Petterson et al, 2009	Not stated	No adverse events were related to the

		exercise intervention. Only 1 patient reported feeling dizzy and lightheaded following the first NMES treatment.
Szots et al, 2016	None	
Vuorenmaa et al, 2014	Not stated	Intervention: 5 patients discontinued training due to pain (2 reported knee pain on operated side, 1 reported knee pain on the contralateral side, 1 reported back pain, and 1 reported hip pain), 1 patient had surgery due to reduced range of motion
