# Supplementary material for: Effectiveness of postdischarge interventions for reducing the severity of chronic pain after total knee replacement: systematic review of randomised controlled trials
Source: BMJ Open. 2018 Feb 28;8(2):e020368. doi: 10.1136/bmjopen-2017-020368 (PMC5855247; doi:10.1136/bmjopen-2017-020368)
Supplement: Author's manuscript [file bmjopen-2017-020368.draft_revisions.pdf]

BMJ Open

BMJ Open is committed to open peer review. As part of this commitment we make the peer review history of every article we publish publicly available.

When an article is published we post the peer reviewers' comments and the authors' responses online. We also post the versions of the paper that were used during peer review. These are the versions that the peer review comments apply to.

The versions of the paper that follow are the versions that were submitted during the peer review process. They are not the versions of record or the final published versions. They should not be cited or distributed as the published version of this manuscript.

BMJ Open is an open access journal and the full, final, typeset and author-corrected version of record of the manuscript is available on our site with no access controls, subscription charges or pay-per-view fees (http://bmjopen.bmj.com).

If you have any questions on BMJ Open's open peer review process please email editorial.bmjopen@bmj.com

BMJ Open

The effectiveness of post-discharge intervention for reducing the severity of chronic pain after total knee replacement: Systematic review of randomised controlled trials

Journal:	*BMJ Open*
Manuscript ID	bmjopen-2017-020368
Article Type:	Research
Date Submitted by the Author:	30-Oct-2017
Complete List of Authors:	Wylde, Vikki; University of Bristol, Musculoskeletal Research Unit<br>Dennis, Jane; University of Bristol, Musculoskeletal Research Unit<br>Gooberman-Hill, Rachael; University of Bristol, School of Clinical Sciences<br>Beswick, Andrew; University of Bristol, School of Clinical Sciences
**Primary Subject Heading**:	Rehabilitation medicine
Secondary Subject Heading:	Surgery, Health services research
Keywords:	Total knee replacement, Chronic post-surgical pain, Prevention, Systematic review

SCHOLARONE™ Manuscripts

For peer review only

For peer review only - http://bmjopen.bmj.com/site/about/guidelines.xhtml

The effectiveness of post-discharge intervention for reducing the severity of chronic pain after total knee replacement: Systematic review of randomised controlled trials

Wylde V[1,2], Dennis J[1], Gooberman-Hill R[1,2], Beswick AD[1]

[1]Musculoskeletal Research Unit, Translational Health Sciences, Bristol Medical School, University of Bristol, UK

[2]National Institute for Health Research Bristol Biomedical Research Centre, University of Bristol, UK

E-mail addresses

Vikki Wylde: v.wylde@bristol.ac.uk

Jane Dennis: jane.dennis@bristol.ac.uk

Rachael Gooberman-Hill: r.gooberman-hill@bristol.ac.uk

Andrew Beswick: andy.beswick@bristol.ac.uk

Corresponding author

Dr Vikki Wylde, Musculoskeletal Research Unit, Translational Health Sciences, Bristol Medical School, Learning and Research Building, Southmead Hospital, Bristol, BS10 5NB, UK

E-mail: V.Wylde@bristol.ac.uk, Telephone: +44 (0)117 414 7878

Word count: 2744

Running title: Post-discharge interventions and chronic pain after TKR

For peer review only - http://bmjopen.bmj.com/site/about/guidelines.xhtml

For peer review only

ABSTRACT

Objective

Approximately 20% of patients experience chronic pain after total knee replacement (TKR). The aim of this systematic review was to evaluate the effectiveness of post-discharge interventions delivered in the first three months after surgery in reducing the severity of chronic pain after TKR

Design

The protocol for this systematic review was registered on PROSPERO (CRD42017041382). MEDLINE, Embase, CINAHL, PsycINFO and *The Cochrane Library* were searched from inception to November 2016. Randomised controlled trials of post-discharge intervention which commenced in the first three months after TKR surgery were included. The primary outcome of the review was self-report pain severity at 12 months or longer after TKR. Risk of bias was assessed using the Cochrane risk-of-bias tool.

Results

Sixteen trials with data from 2,451 randomised participants were included. The majority of trials evaluated physiotherapy interventions (n=12); other interventions included nurse-led interventions, neuromuscular electrical stimulation and a multidisciplinary intervention. Meta-analysis of six studies comparing physiotherapy with usual care found no difference in long-term pain (SMD -014, 95% CI -0.41, 0.13). Narrative synthesis supported the findings from meta-analysis. For non-physiotherapy interventions, there was insufficient evidence to draw conclusions about effectiveness.

Conclusion

For peer review only - http://bmjopen.bmj.com/site/about/guidelines.xhtml

This systematic review found evidence that post-discharge physiotherapy interventions delivered uniformly to all patients in the first three months after TKR do not appear to be effective at reducing the severity of chronic pain. Further research is needed to evaluate whether stratified physiotherapy care and multidisciplinary interventions can reduce the severity of chronic pain after TKR.

Key words: Total knee replacement, chronic post-surgical pain, prevention, systematic review

Strengths and limitations of this study

- This is the first systematic review to evaluate the effectiveness of post-discharge interventions delivered in the first three months after surgery in reducing the severity of chronic pain after total knee replacement.
- Synthesis of adverse events data was not possible because assessment and reporting was variable and often poor.
- We did not include studies that used a composite pain and function measure to assess pain outcome.

For peer review only

For peer review only - http://bmjopen.bmj.com/site/about/guidelines.xhtml

INTRODUCTION

Total knee replacement (TKR) is a common operation to provide pain relief, predominately due to osteoarthritis. Despite good outcomes for many, some patients report chronic pain in the months and years after TKR. Chronic post-surgical pain is defined as pain that is present or increases in intensity at ≥3 months after surgery [1]. In representative populations, unfavourable long-term pain outcomes have been reported by 10-34% of patients with TKR [2]. Patients with bothersome pain at ≥3 months after surgery are disappointed with their outcome [3 4]. Given the prevalence and impact of chronic pain, it is important to evaluate interventions that may optimise patients' outcomes after TKR.

During the hospital stay after TKR, rehabilitation focuses on regaining range of motion and improving mobility. After discharge, rehabilitation aims to enhance recovery, through supporting a person to regain function and quality of life, optimising pain relief and re-integration into social and personal environments [5]. While physiotherapy often focusses on functional health, another key outcome is the prevention of long-term pain [6]. Post-operative physiotherapy may be combined with other interventions to provide comprehensive, multidisciplinary and holistic rehabilitation [7]. Therefore, a key step to improving patients' outcomes after TKR is to evaluate if early post-operative rehabilitation interventions can reduce the severity of chronic pain after TKR.

The aim of this systematic review was to evaluate the effectiveness of post-discharge interventions delivered in the first three months after surgery for reducing the severity of chronic pain after TKR.

For peer review only

METHODS

The review was registered on the international prospective register of systematic reviews (PROSPERO) on 17th January 2017 (registration number CRD42017041382). The review was conducted following guidance from the Cochrane Handbook [8] and reported in accordance with PRISMA guidelines [9] (Appendix 1).

Eligibility criteria

Studies were eligible for inclusion in the review if they met the following criteria:

Population: Adults discharged from hospital after primary TKR predominantly for osteoarthritis.

Intervention: Any post-discharge intervention which commenced in the first three months after TKR surgery.

Control: Any, including no intervention, usual care, placebo or an alternative intervention.

Outcomes: The primary outcome was pain severity at 12 months or longer after TKR, as patient-reported levels of pain plateau by this time point [10 11]. Pain severity could be assessed using a patient-reported joint-specific pain measure (e.g. WOMAC or KOOS pain domains), a quality of life measure (e.g. SF-36 or SF-12) or a Visual Analogue Scale (VAS). The secondary outcome was serious adverse events.

Study type: Randomised controlled trials (RCTs).

Information sources and searches

MEDLINE, Embase, CINAHL, PsycINFO and *The Cochrane Library* were searched from inception to 15th November 2016 (Appendix 2). No language restrictions were applied and relevant non-English articles were translated and included if appropriate. Studies reported

For peer review only - http://bmjopen.bmj.com/site/about/guidelines.xhtml

For peer review only

only as abstracts or that were unobtainable as full text copies using inter-library loans or email contact with authors were excluded. Citations of key reviews and studies were checked in ISI Web of Science.

Screening

Records identified by searches were imported into Endnote X7 (Thomson Reuters) and duplicates removed. From the searches, an Endnote database of all RCTs and systematic reviews in TKR was established. Within this database, interventions conducted during the post-operative period were identified. An initial screen for potential eligibility was undertaken by one reviewer (ADB) to exclude articles that were clearly not relevant. Subsequently, abstracts and full-text articles were screened independently by two reviewers (VW and ADB or JD) and reasons for exclusion recorded.

Data extraction

Data from studies that met the eligibility criteria were extracted onto a standardised proforma by one reviewer (VW). Data extraction was checked against source articles by a second reviewer (JD). Extracted data comprised: country, date, participant characteristics, selection criteria; intervention and control treatment; follow up intervals; losses to follow-up; outcome data for pain (means and standard deviations or medians and ranges); serious adverse events and information for risk of bias assessment. Any disagreements between reviewers were discussed with a third reviewer (ADB) and consensus reached.

A single e-mail was sent to authors of studies with an appropriate follow-up period but no pain outcome to enquire if an appropriate outcome was available. If a combined pain and function outcome was reported, such as the OKS or the total WOMAC score, separate pain subscale data were requested. Authors were contacted when necessary for clarification purposes or to request unpublished relevant data. If a study reported data that were combined

For peer review only - http://bmjopen.bmj.com/site/about/guidelines.xhtml

for knee and hip replacement patients, then disaggregated data for patients with TKR were requested. If this was not available, then the study was excluded.

Risk of bias assessment

Potential sources of bias were assessed using the Cochrane risk of bias tool [8]. At the protocol stage, analysis was planned which included all studies, with sensitivity analyses conducted to exclude studies judged to be at high risk of bias.

Strategy for data synthesis

At the protocol stage, meta-analysis using RevMan 5 [12] was planned if two or more studies were identified with similar interventions and comparator groups and appropriate outcome data. If continuous pain outcomes were measured differently across studies, overall standardised mean differences and 95% confidence intervals would be calculated and presented alongside measures of heterogeneity (I^2). Where possible, subgroup analyses were planned to explore the effectiveness of different intervention content and intensity, and different comparator interventions. When pooling outcome data was not appropriate, a narrative synthesis was planned.

RESULTS

Searches identified 7,954 articles. After detailed evaluation of full-text articles, 16 studies with 2,451 randomised participants were included [13-28] (Figure 1). Two included studies were published after the search dates, but were identified from protocols published within the search dates [14 16].

Study characteristics

For peer review only

For peer review only - http://bmjopen.bmj.com/site/about/guidelines.xhtml

Table 1 provides an overview of study characteristics. Included studies were from Australia (n=3), Canada (n=2), Finland (n=2), Germany (n=2), United Kingdom (n=2), China (n=1), Denmark (n=1), Italy (n=1), Norway (n=1) and United States of America (n=1). The number of centres was reported for 14 studies: seven studies were conducted in a single centre, three studies were conducted in two centres, and four studies were conducted in ≥4 centres. Sample sizes ranged from 47 to 422 participants, with a median of 138. All studies had two arms, with the exception of one three-arm trial [19]. Three studies were described as pilot or feasibility studies [15 17 23].

Study quality

Risk of bias assessments for individual studies are displayed in Table 2. All studies were at high risk of bias for blinding of participants and pain outcome assessment due to the nature of the intervention and the self-reporting of pain. Six studies were at high risk of bias due to incomplete outcome data. Possible risk of selective outcome reporting was identified for two studies.

Outcomes assessment

Pain was most commonly assessed using the WOMAC pain scale (n=9); other tools included the KOOS pain scale (n=4), VAS (n=1), and single items from the OKS (n=1) and KOS ADL (n=1). Serious adverse events were poorly described and reported in the majority of studies and therefore pooling of harms data was not possible. A summary of adverse events findings is presented in Appendix 3. Pain was assessed at 12 months after TKR in 15 studies and at 14 months in one study. A summary of results from the individual studies is provided in Appendix 4.

Interventions

For peer review only - http://bmjopen.bmj.com/site/about/guidelines.xhtml
For peer review only

The majority of studies evaluated physiotherapy interventions (n=12); other interventions evaluated included nurse-led interventions (n=2), neuromuscular electrical stimulation (NMES) (n=1) and a multidisciplinary intervention (n=1).

Physiotherapy interventions

Twelve studies with 1,846 randomised patients evaluated the effectiveness of post-discharge physiotherapy interventions. There was considerable variation in the interventions evaluated. Seven studies compared physiotherapy interventions with usual or minimal care; these interventions included a walking skills programme [13], group-based circuit exercise classes [16], erogometer cycling [21], home-based functional rehabilitation [23], clinic-based functional rehabilitation [24], home-based functional exercises aimed at managing kinesiophobia [25], and delayed monitored home exercises [28]. Five studies compare two forms of treatment including inpatient rehabilitation compared with home exercise [14], home-based functional exercise and home-based traditional exercise [17], 1:1 physiotherapy and home-based rehabilitation [20], early aquatic therapy and late aquatic therapy [22], and a three-arm trial comparing 1:1 physiotherapy, group-based circuit classes and a monitored home exercise programme [19]. All interventions started within two months of surgery, with the majority commencing within two weeks of surgery. Of the 12 studies, only one trial reported a difference in pain outcomes between groups; patients randomised to home-based exercises aimed at managing kinesiophobia had lower pain scores at 12 months post-operative compared with patients randomised to usual care [25].

Meta-analysis was conducted with six studies that reported relevant pain outcome data to compare the effectiveness of physiotherapy interventions compared with usual care for reducing the severity of chronic pain after TKR [13 16 21 23-25] (Figure 2). Standardised mean differences were pooled using a random effects meta-analysis. No differences in pain

For peer review only - http://bmjopen.bmj.com/site/about/guidelines.xhtml

outcomes were observed between treatments, with a standardised mean difference of -0.14 (95% CI -0.41, 0.13; I^2 69%). Similar results were obtained in sensitivity analysis excluding two studies at high risk of bias from incomplete outcome data [21 24] (Appendix 5).

Due to the small number of studies, subgroup analyses to explore the effectiveness of different intervention content and intensity, and different comparator interventions, were not possible.

Nurse-led interventions

Two studies with 319 randomised patients reported evaluation of a nurse-led intervention compared with no care or usual care. Except for issues relating to blinding, both studies were at low risk of bias. Both studies evaluated nurse-led structured telephone follow-up; one aimed to improve adherence to home exercise [15] and the other to provide information regarding well-being, integrity, prophylaxis, safety and other issues relevant to patients after TKR [27]. Pain outcome data (mean and standard deviations) were not available for latter study and therefore meta-analysis was not possible. Neither study found a difference in pain scores at 12 months post-operative between the intervention and control group.

Other interventions

Two studies reported evaluations of other interventions. Except for issues relating to blinding, both studies were at low risk of bias. One trial involving 86 patients compared a group-based multidisciplinary programme with usual care [18]. This 10-day programme involved physiotherapy, Nordic walking, relaxation strategies, and sessions with a psychologist, social worker, nutritionist and orthopaedic surgeon. Another trial involving 200 patients evaluated a combined NMES and volitional strength training programme compared with volitional

For peer review only - http://bmjopen.bmj.com/site/about/guidelines.xhtml

For peer review only

strength training program without NMES [26]. Both studies found no difference in pain scores at 12 months post-operative between the intervention and control group.

Ongoing research

In searches of databases and citation searches on ISI Web of Science we identified a number of published RCT protocols that are evaluating post-discharge interventions with a pain outcome at ≥12 months after TKR. Interventions being evaluated include a digital activity coaching system for home exercise [29], Wii-enhanced rehabilitation [30], group-based outpatient physiotherapy with an individualised element [31], multicomponent rehabilitation for patients at risk of a poor outcome [32] and physiotherapy for patients performing poorly at six weeks after TKR [33]. Some of these studies are now finished and findings are likely to be reported imminently.

DISCUSSION

This systematic review aimed to evaluate the effectiveness of post-discharge interventions delivered in the first three months after surgery for reducing the severity of chronic pain after TKR. Interventions that predominately comprise physiotherapy have been evaluated in RCTs. The evidence suggests that post-discharge physiotherapy interventions appear not be effective at reducing the severity of chronic pain after TKR, although findings from the trial of home-based functional exercise programmed aimed at managing kinesiophobia [25] were encouraging and warrant further evaluation. There was insufficient evidence to draw conclusions about the effectiveness of non-physiotherapy interventions, and further research is needed.

For peer review only - http://bmjopen.bmj.com/site/about/guidelines.xhtml

There are a number of strengths and limitations to this systematic review. The main outcome of interest in this review was pain severity at ≥12 months after TKR. Although the primary outcome of many of the included studies was function, pain severity was an important secondary outcome in these studies. Studies that used a composite pain and function measure to assess outcome, for example the OKS or WOMAC, were excluded if authors were unable to provide pain subscales scores. Although this reduced the number of studies eligible for inclusion, this approach was taken because pain and function are distinct outcome domains, with different predictors and recovery trajectories [34 35]. The secondary outcome of this review was adverse events, to allow the synthesis of harms data. However, synthesis was not possible because assessment and reporting of adverse events was variable and often poor. The quality of adverse events reporting is a common issue in surgical trials [36], and evidence-based recommendations are needed to promote standardisation, improve quality and reduce heterogeneity of adverse events reporting in orthopaedic studies. A potential limitation of the included studies was that they were all at high risk of bias due to the lack of participant blinding for self-report pain. However, blinding of participants is rarely possible in RCTs of this nature. Also, it would be expected that the risk would arise from participants in the intervention group reporting less pain, which may potentially be an issue with shorter-term outcomes, but this was not evident from the longer-term follow-up of the studies included in this review.

This systematic review took a broad approach by evaluating the effectiveness of any type of post-discharge intervention that aimed to reduce the severity of chronic pain after TKR. Previous systematic reviews of interventions to improve long-term outcomes after TKR have been conducted, but these have evaluated pre-operative interventions or have been narrower in focus. Systematic review of pre-operative interventions have found that exercise and education have a limited effect on improving pain and function after TKR [37-39]. Previous

For peer review only - http://bmjopen.bmj.com/site/about/guidelines.xhtml

systematic reviews of post-discharge interventions have focussed on physiotherapy, finding some evidence of short-term benefit but not long-term benefit [6 40]. One systematic review has evaluated interventions for the management of chronic pain after TKR, identifying only a single RCT of botulinum toxin A injections [41]. Our systematic review adds to this literature by providing evidence that post-discharge physiotherapy interventions do not appear to be effective at reducing the severity of chronic pain after TKR and highlighting the need for further research on other intervention types.

It is important to consider potential reasons why post-discharge physiotherapy does not appear to be effective at reducing the severity of chronic pain after TKR. One possible reason is that the interventions were uniformly delivered to all patients, rather than just those patients who may be at most risk of poor outcome. Only 20% of patients will develop chronic pain after TKR [2] and delivering physiotherapy to all patients may reduce the ability to detect clinical benefit in terms of pain severity. This suggests a need to identify patients at high risk of chronic pain and provide these patients with intensive and comprehensive interventions that have been specifically designed to reduce their risk of developing chronic pain. However, identifying high risk patients is challenging; pre-operative models to identify patients at risk of a poor outcome have low predictive power [34 35], and the evidence for post-operative risk factors is limited [42]. However, research is currently ongoing to evaluate whether providing a rehabilitation programme for patients at risk of a poor outcome [43] or are 'functioning poorly' at six weeks after TKR [33] can improve longer-term outcomes.

Another possible explanation for the apparent lack of effectiveness of post-operative physiotherapy in reducing severity of chronic pain is the complexity of chronic pain after TKR. Although chronic pain after TKR is not yet fully understood, the aetiology of this pain is multifactorial, including surgical factors, complex regional pain syndrome, pain sensitisation, neuropathic pain and psychosocial factors [44-49]. Therefore, an intervention

For peer review only - http://bmjopen.bmj.com/site/about/guidelines.xhtml

comprising a single treatment modality, such as physiotherapy, may be insufficient to address and reduce the causes of pain for all patients. As with the treatment of other chronic pain conditions, this highlights the importance of focused, individualised and multidisciplinary treatment [50 51]. Such an approach is being evaluated in an ongoing RCT of a care pathway for patients with chronic pain after TKR (ISRCTN92545361). Therefore, stratified physiotherapy care, in the context of individualised assessment and a multidisciplinary care package, may be useful for some patients.

In conclusion, this systematic review found evidence that post-discharge physiotherapy interventions delivered uniformly to all patients in the first three months after TKR do not appear to be effective at reducing the severity of chronic pain. Further research is needed to evaluate whether stratified physiotherapy care and multidisciplinary interventions can reduce the severity of chronic pain after TKR.

For peer review only

For peer review only - http://bmjopen.bmj.com/site/about/guidelines.xhtml

ACKNOWLEDGEMENTS

We would like to thank all the study authors who took the time to reply to our requests for further clarification or additional data.

AUTHOR CONTRIBUTIONS

All authors contributed to the concept and design of the study. ADB, JD and VW contributed to the acquisition and analysis of data. VW drafted the article and ADB, JD and RGH revised it critically for important intellectual content. VW and ADB take responsibility for the integrity of the work as a whole, from inception to finished article.

FUNDING

This article presents independent research funded by the National Institute for Health Research (NIHR) under its Programme Grants for Applied Research programme (RP-PG-0613-20001). The views expressed are those of the authors and not necessarily those of the NHS, the NIHR or the Department of Health. The funder had no involvement in the study design, data collection, data analysis, interpretation of data or writing of the manuscript.

This study was supported by the NIHR Biomedical Research Centre at the University Hospitals Bristol NHS Foundation Trust and the University of Bristol. The views expressed in this publication are those of the author(s) and not necessarily those of the NHS, the National Institute for Health Research or the Department of Health.

COMPETING INTERESTS

The authors have no conflicts of interest to declare.

DATA SHARING STATEMENT

No additional data are available.

For peer review only - http://bmjopen.bmj.com/site/about/guidelines.xhtml

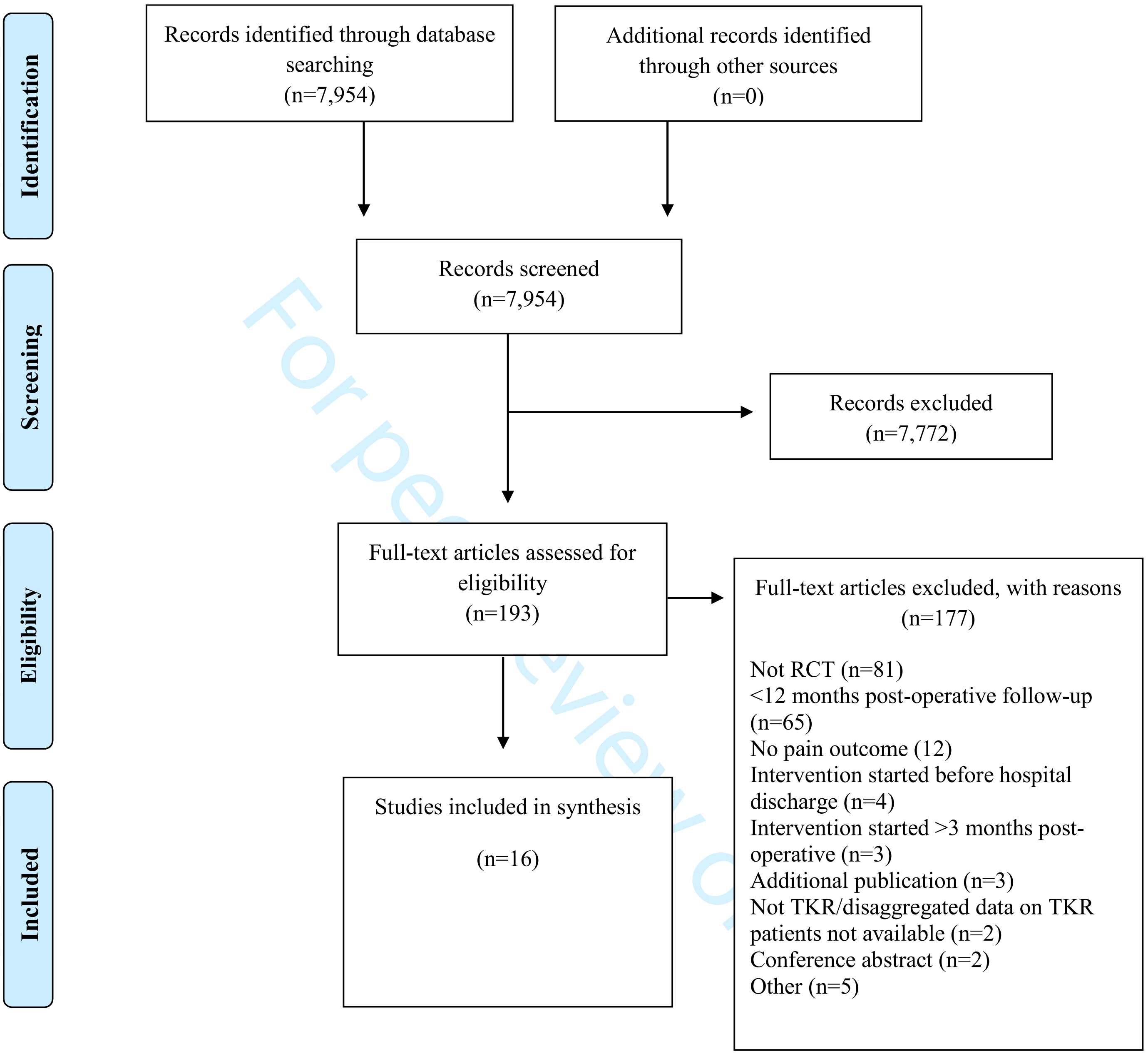

Figure 1: Systematic review flow diagram

For peer review only - http://bmjopen.bmj.com/site/about/guidelines.xhtml

Table 1: Overview of study characteristics

Publication<br>Location<br>Date of study<br>Number of centres	Randomised<br>Mean age<br>% female	Intervention treatment	Control treatment	Pain assessment<br>Adherence to treatment<br>Losses to follow-up
Bruun-Olsen et al, 2013<br>Norway<br>2008-2010<br>2 centres	N=57(29:28)<br>68:69 years<br>62:50%	Group-based physiotherapist-led walking skills programme (2-6 patients per group). Commenced 6 weeks after surgery. 12 sessions over 6-8 weeks.	1:1 usual physiotherapy care	KOOS pain scale<br>28/29 completed intervention<br>28/28 received control treatment<br>6 (2:4) lost to follow-up
Buhagiar et al, 2017<br>Australia<br>2012-2015<br>2 centres	N=165(81:84)<br>67:67 years<br>69:68%	Inpatient rehabilitation at rehabilitation facility with twice daily supervised sessions of 1:1 and group-based exercises. Commenced after hospital discharge for 10 days. Home exercise programme after discharge from rehabilitation facility.	Home exercise programme comprising of 2-3 group-based outpatient session to practice and progress exercises. Commenced 2 weeks after surgery.	KOOS pain scale<br>72/81 adhered to intervention<br>74/84 adhered to control treatment<br>6(2:4) lost to follow-up
Chen et al, 2016<br>China<br>2013-2014<br>1 centre	N=202(101:101)<br>66:67 years<br>63:67%	Structured telephone follow-up by nurse at 1, 3 and 6 weeks after hospital discharge to improve adherence to home exercise routine.	No telephone follow-up	Pain VAS<br>Adherence not reported<br>15(7:8) lost to follow-up
Fransen et al, 2017<br>Australia<br>2009-2012<br>12 centres	N=422(212:210)<br>64:65 years<br>54:52%	Group-based circuit exercise classes supervised by physiotherapist. Up to 6 patients per class. Commenced 6 weeks after surgery. Twice weekly sessions for at least 8 weeks.	Usual physiotherapy care	WOMAC pain scale<br>140/212 participants attended ≥12 classes<br>210/210 received control treatment<br>74(43:31) lost to follow-up
Frost et al, 2002<br>UK<br>1995-1996<br>Not reported	N=47(23:24)<br>72:71 years<br>48:50%	Home-based functional exercise. Commenced following discharge from hospital. Duration not reported.	Home-based traditional exercise.	OKS item (pain on walking)<br>Adherence not reported<br>20 (7:13) lost to follow-up
Kauppila et al, 2010$<br>Finland	N=86(44:42)<br>71:71 years	Group-based multidisciplinary rehabilitation programme. Up to 8 patients per group.	Usual physiotherapy care.	WOMAC pain scale<br>44/44 attended intervention

For peer review only - http://bmjopen.bmj.com/site/about/guidelines.xhtml

2002-2005<br>1 centre	76:79%	Commenced 2-4 months after surgery for 10 days.		42:42 received control treatment<br>11 (8:3) lost to follow-up
Ko et al, 2013<br>Australia<br>2008-2010<br>3 arm trial<br>4 centres	N=<br>249(85:84:80)<br>67:68:67 years<br>68:60:61%	1:1 physiotherapy with home-based sessions. Commenced 2 weeks after surgery. Twice weekly 1:1 and home-based sessions over 6 weeks.	1.) Group-based circuit classes supervised by physiotherapist with home-based sessions. Up to 8 patients per class. Commenced 2 weeks after surgery. Twice weekly group and home-based sessions over 6 weeks.<br><br>2.) Monitored home programme, 2 1:1 physiotherapy sessions and 1 telephone follow-up call. Commenced 2 weeks after surgery. 4 sessions per week for 6 weeks.	WOMAC pain scale<br>80% participants attended 9 or more 1:1 sessions, 77% attended 9 or more group sessions, 83% attended both sessions in monitored home programme group.<br>16(7:3:6) lost to follow-up
Kramer et al, 2003<br>Canada<br>Not reported<br>Not reported	N=160(80:80)<br>68:69 years<br>59:55%	1:1 clinic-based rehabilitation programme with home exercise programme. Commenced 2 weeks after surgery. Up to 2 sessions a week for 10 weeks.	Home-based rehabilitation, monitored by telephone calls from physiotherapist. Commenced 2 weeks after surgery. At least 1 telephone call in weeks 2-6 and 1 call in weeks 7-12.	WOMAC pain scale<br>76/80 received intervention<br>78/80 received control treatment<br>26 (15:22) lost to follow-up
Liebs et al, 2010*<br>Germany<br>2005-2006<br>5 centres	N=159(85:74)<br>70:70 years<br>73:70%	Ergometer cycling supervised by physiotherapist. Commenced 2 weeks after surgery. 3 sessions a week for at least 3 weeks.	Usual physiotherapy care	WOMAC pain scale<br>Adherence not reported<br>33(15:18) lost to follow-up
Liebs et al, 2012*<br>Germany<br>2003-2004<br>2 centres	N=185(87:98)<br>69:71 years<br>70:73%	Early aquatic therapy. Commenced 6 days after surgery. 3 times a week up to 5th week post-operative.	Late aquatic therapy. Commenced 14 days after surgery. 3 times a week up to 5th week post-operative.	WOMAC pain scale<br>Adherence not reported<br>41(18:23) lost to follow-up

For peer review only

For peer review only - http://bmjopen.bmj.com/site/about/guidelines.xhtml

Minns Lowe et al, 2012<br>UK<br>2006-2008<br>1 centre	N=107 (56:51)<br>68:71 years<br>57:59%	Home-based functional rehabilitation with 2 visits from physiotherapist at 2 weeks and 6-8 weeks after hospital discharge. Twice daily exercises for at least 3 months.	Usual physiotherapy care	KOOS pain scale<br>46/56 patients received 2 visits<br>47/51 received control treatment<br>9 (7:2) lost to follow-up
Moffet et al, 2004 [24]<br>Canada<br>1997-1999<br>5 centres	N=77(38:39)<br>67:69 years<br>63:56%	Functional rehabilitation programme with individualised home exercises. Commenced at 2 months after surgery. 12 supervised sessions over 6-8 weeks.	Usual physiotherapy care.	WOMAC pain scale<br>38/38 participated in 12 sessions<br>39/39 received control treatment<br>8(0:8) lost to follow-up
Monticone et al, 2010-2013<br>Italy<br>1 centre	N=110(55:55)<br>67:68 years<br>65:62%	Home-based functional exercises aimed at managing kinesiophobia, with monthly phone calls to encourage adherence. Commenced after hospital discharge. Twice weekly sessions for 6 months.	No physiotherapy, advice to stay active.	KOOS pain scale<br>Adherence not reported<br>0 lost to follow-up
Petterson et al, 2009<br>USA<br>200-2005<br>1 centre	N=200(100:100)<br>65:65 years<br>47:45%	Combined neuromuscular electric stimulation (NMES) and volitional strength training programme. Commenced 3-4 weeks after surgery. 2 or 3 sessions a week for 6 weeks.	Volitional strength training program without NMES.	KOS ADL item (effect of pain on function)<br>84/100 completed intervention<br>97/100 completed control treatment<br>51 (32:19) lost to follow-up
Szots et al, 2016<br>Denmark<br>2013<br>1 centre	N=117(59:58)<br>67:68 years<br>61:67%	Two nurse-led structured telephone follow-up calls. Telephone calls at 4 days and 14 days after hospital discharge.	Usual care	WOMAC pain scale<br>54/59 patients had both telephone follow-up calls<br>54/58 received control treatment<br>9(5:4) lost to follow-up
Vuorenmaa et al, 2014 **<br>Finland<br>2008-2010<br>1 centre	N=108(53:55)<br>69:69 years<br>57:65%	Delayed monitored home exercises, with guidance from physiotherapist at 2, 3 and 6 months post-operative. Commenced at 2 months after surgery for 12 months.	Usual care	WOMAC pain scale<br>72% of patients performed the training sessions at least twice per week in the first 6 months<br>53/53 received control treatment<br>4(2:2) lost to follow-up

* 24 month follow-up also conducted but data from 12 month follow-up included in table to be consistent with follow-up period of other studies

For peer review only - http://bmjopen.bmj.com/site/about/guidelines.xhtml

** Follow-up at 14 months post-operative
$ Pain-specific outcome data was provided by the authors for a previous review [52] and was used again in this review

For peer review only

For peer review only - http://bmjopen.bmj.com/site/about/guidelines.xhtml

Table 2: Risk of bias assessment for individual studies

	Random sequence generation (selection bias)	Allocation concealment (selection bias)	Blinding of participants and personnel (performance bias)	Blinding of outcome assessment (detection bias)	Incomplete outcome data (attrition bias)	Selective reporting (reporting bias)	Other bias
Bruun-Olsen 2013	+	+	−	−	+	+	+
Buhagiar 2017	+	+	−	−	+	+	+
Chen 2016	+	+	−	−	+	?	+
Fransen 2017	+	+	−	−	+	+	+
Frost 2002	+	?	−	−	−	?	+
Kauppila 2010	+	+	−	−	+	+	+
Ko 2013	+	+	−	−	+	+	+
Kramer 2003	?	?	−	−	+	+	+
Liebs 2010	+	+	−	−	−	+	+
Liebs 2012	+	+	−	−	−	?	+
Minns Lowe 2012	+	+	−	−	+	+	+
Moffet 2004	+	+	−	−	−	?	+
Monticone 2013	+	+	−	−	+	?	+
Petterson 2009	?	?	−	−	−	−	+
Szots 2016	+	+	−	−	+	+	+
Vuornmaa 2014	+	+	−	−	+	+	+

For peer review only - http://bmjopen.bmj.com/site/about/guidelines.xhtml

Study or Subgroup	Physiotherapy Mean	Physiotherapy SD	Physiotherapy Total	Usual care Mean	Usual care SD	Usual care Total	Weight	Std. Mean Difference IV, Random, 95% CI
Bruun-Olsen 2013	-82	21	29	-74	23	28	13.0%	-0.36 [-0.88, 0.17]
Fransen 2017	2.6	2.6758	169	2.5	2.6	179	21.9%	0.04 [-0.17, 0.25]
Liebs 2010	15.6	17.9	70	13	14.9	56	17.7%	0.16 [-0.20, 0.51]
Minns Lowe 2012	-77.49	18.96	49	-80.1	22.18	49	16.4%	0.13 [-0.27, 0.52]
Moffet 2004	9.4	12.4	38	11.8	13	31	14.2%	-0.19 [-0.66, 0.29]
Monticone 2013	-87.35	11.71	55	-77.38	15.07	55	16.7%	-0.73 [-1.12, -0.35]
**Total (95% CI)**			**410**			**398**	**100.0%**	**-0.14 [-0.41, 0.13]**

Heterogeneity: Tau² = 0.08; Chi² = 16.22, df = 5 (P = 0.006); I² = 69%
Test for overall effect: Z = 1.01 (P = 0.31)

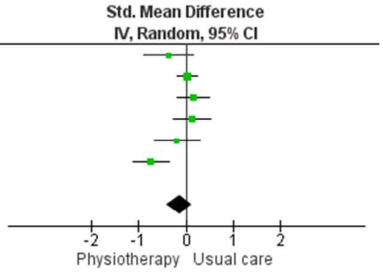

Figure 2: Forest plot of the effectiveness of post-discharge physiotherapy vs. usual care in reducing chronic pain after TKR

For peer review only

REFERENCES

1. Werner MU, Kongsgaard UE. I. Defining persistent post-surgical pain: is an update required? Br J Anaesth 2014;**113**(1):1-4.

2. Beswick AD, Wylde V, Gooberman-Hill R, et al. What proportion of patients report long-term pain after total hip or knee replacement for osteoarthritis? A systematic review of prospective studies in unselected patients. BMJ Open 2012;**2**(1):e000435.

3. Scott CE, Howie CR, MacDonald D, et al. Predicting dissatisfaction following total knee replacement: a prospective study of 1217 patients. J Bone Joint Surg Br 2010;**92**(9):1253-8.

4. Jeffery AE, Wylde V, Blom AW, et al. "It's there and I'm stuck with it": patients' experiences of chronic pain following total knee replacement surgery. Arthritis Care Res (Hoboken) 2011;**63**(2):286-92.

5. Artz N, Dixon S, Wylde V, et al. Physiotherapy provision following discharge after total hip and total knee replacement: a survey of current practice at high-volume NHS hospitals in England and wales. Musculoskeletal Care 2013;**11**(1):31-8.

6. Artz N, Elvers K, Minns Lowe C, et al. Effectiveness of physiotherapy exercise following total knee replacement: systematic review and meta-analysis. BMC Musculoskeletal Disorders 2015;**16**(15).

7. Khan F, Ng L, Gonzalez S, et al. Multidisciplinary rehabilitation programmes following joint replacement at the hip and knee in chronic arthropathy. Cochrane Database Syst Rev 2008(2):CD004957.

8. Higgins J, Green SE. Cochrane Handbook for Systematic Reviews of Interventions Version 5.1.0 [updated March 2011]. The Cochrane Collaboration, 2011. Available from www.cochrane-handbook.org, 2011.

9. Moher D, Liberati A, Tetzlaff J, et al. Preferred reporting items for systematic reviews and meta-analyses: the PRISMA statement. BMJ 2009;**339**:b2535.

10. Lenguerrand E, Wylde V, Gooberman-Hill R, et al. Trajectories of Pain and Function after Primary Hip and Knee Arthroplasty: The ADAPT Cohort Study. PLoS One 2016;**11**(2):e0149306.

11. Kennedy DM, Stratford PW, Hanna SE, et al. Modeling early recovery of physical function following hip and knee arthroplasty. BMC Musculoskelet Disord 2006;**7**:100.

For peer review only - http://bmjopen.bmj.com/site/about/guidelines.xhtml

12. Review Manager. (RevMan) [Computer program]. Version 5.3. Copenhagen: The Nordic Cochrane Centre, The Cochrane Collaboration. 2014.

13. Bruun-Olsen V, Heiberg KE, Wahl AK, et al. The immediate and long-term effects of a walking-skill program compared to usual physiotherapy care in patients who have undergone total knee arthroplasty (TKA): a randomized controlled trial. Disability and rehabilitation 2013;**35**(23):2008-15.

14. Buhagiar MA, Naylor JM, Harris IA, et al. Effect of Inpatient Rehabilitation vs a Monitored Home-Based Program on Mobility in Patients With Total Knee Arthroplasty: The HIHO Randomized Clinical Trial. JAMA 2017;**317**(10):1037-46.

15. Chen M, Li P, Lin F. Influence of structured telephone follow-up on patient compliance with rehabilitation after total knee arthroplasty. Patient Preference and Adherence 2016;**10**:257-64.

16. Fransen M, Nairn L, Bridgett L, et al. Post-Acute Rehabilitation After Total Knee Replacement: A Multicenter Randomized Clinical Trial Comparing Long-Term Outcomes. Arthritis Care Res (Hoboken) 2017;**69**(2):192-200.

17. Frost H, Lamb SE, Robertson S. A randomized controlled trial of exercise to improve mobility and function after elective knee arthroplasty. Feasibility, results and methodological difficulties. Clinical rehabilitation 2002;**16**(2):200-9.

18. Kauppila AM, Kyllonen E, Ohtonen P, et al. Multidisciplinary rehabilitation after primary total knee arthroplasty: a randomized controlled study of its effects on functional capacity and quality of life. Clinical rehabilitation 2010;**24**(5):398-411.

19. Ko V, Naylor J, Harris I, et al. One-to-one therapy is not superior to group or home-based therapy after total knee arthroplasty: a randomized, superiority trial. The Journal of bone and joint surgery. American volume 2013;**95**(21):1942-9.

20. Kramer JF, Speechley M, Bourne R, et al. Comparison of clinic- and home-based rehabilitation programs after total knee arthroplasty. Clinical orthopaedics and related research 2003(410):225-34.

21. Liebs TR, Herzberg W, Ruther W, et al. Ergometer cycling after hip or knee replacement surgery: a randomized controlled trial. The Journal of bone and joint surgery. American volume 2010;**92**(4):814-22.

22. Liebs T, Herzberg W, Rüther W, et al. Multicenter randomized controlled trial comparing early versus late aquatic therapy after total hip or knee arthroplasty. Archives of physical medicine and rehabilitation 2012; 93(2).

For peer review only

For peer review only - http://bmjopen.bmj.com/site/about/guidelines.xhtml

http://onlinelibrary.wiley.com/o/cochrane/clcentral/articles/334/CN-00832334/frame.html

http://ac.els-cdn.com/S0003999311008434/1-s2.0-S0003999311008434-main.pdf?_tid=41aa00ce-f508-11e6-b06b-00000aab0f27&acdnat=1487332829_31eb2562ac20096dc09cc02ed9cf1f7e.

23. Minns Lowe CJ, Barker KL, Holder R, et al. Comparison of postdischarge physiotherapy versus usual care following primary total knee arthroplasty for osteoarthritis: an exploratory pilot randomized clinical trial. Clinical rehabilitation 2012;**26**(7):629-41.

24. Moffet H, Collet JP, Shapiro SH, et al. Effectiveness of intensive rehabilitation on functional ability and quality of life after first total knee arthroplasty: A single-blind randomized controlled trial. Archives of physical medicine and rehabilitation 2004;**85**(4):546-56.

25. Monticone M, Ferrante S, Rocca B, et al. Home-based functional exercises aimed at managing kinesiophobia contribute to improving disability and quality of life of patients undergoing total knee arthroplasty: a randomized controlled trial. Archives of physical medicine and rehabilitation 2013;**94**(2):231-9.

26. Petterson SC, Mizner RL, Stevens JE, et al. Improved function from progressive strengthening interventions after total knee arthroplasty: a randomized clinical trial with an imbedded prospective cohort. Arthritis and rheumatism 2009;**61**(2):174-83.

27. Szöts K, Konradsen H, Solgaard S, et al. Long-term effects of telephone follow-up after total knee arthroplasty. Journal of Nursing Education and Practice 2016;**6**(7):151-54.

28. Vuorenmaa M, Ylinen J, Piitulainen K, et al. Efficacy of a 12-month, monitored home exercise programme compared with normal care commencing 2 months after total knee arthroplasty: a randomized controlled trial. Journal of rehabilitation medicine 2014;**46**(2):166-72.

29. Harmelink KEM, Zeegers A, Tonis TM, et al. The effectiveness of the use of a digital activity coaching system in addition to a two-week home-based exercise program in patients after total knee arthroplasty: study protocol for a randomized controlled trial. BMC Musculoskelet Disord 2017;**18**(1):290.

30. Negus JJ, Cawthorne DP, Chen JS, et al. Patient outcomes using Wii-enhanced rehabilitation after total knee replacement - the TKR-POWER study. Contemp Clin Trials 2015;**40**:47-53.

For peer review only - http://bmjopen.bmj.com/site/about/guidelines.xhtml

31. Wylde V, Artz N, Marques E, et al. Effectiveness and cost-effectiveness of outpatient physiotherapy after knee replacement for osteoarthritis: study protocol for a randomised controlled trial. Trials 2016;**17**(1):289.

32. Barker KL, Beard D, Price A, et al. COmmunity-based Rehabilitation after Knee Arthroplasty (CORKA): Study protocol for a randomised controlled trial. Trials 2016;**17**(1):no pagination.

33. Simpson AH, Hamilton DF, Beard DJ, et al. Targeted rehabilitation to improve outcome after total knee replacement (TRIO): study protocol for a randomised controlled trial. Trials 2014;**15**:44.

34. Judge A, Arden NK, Cooper C, et al. Predictors of outcomes of total knee replacement surgery. Rheumatology (Oxford) 2012;**51**(10):1804-13.

35. Lopez-Olivo MA, Landon GC, Siff SJ, et al. Psychosocial determinants of outcomes in knee replacement. Ann Rheum Dis 2011;**70**(10):1775-81.

36. Wylde V, Dennis J, Beswick AD, et al. Systematic review of management of chronic pain after surgery. British Journal of Surgery 2017;**104**(10):1293-306.

37. Blom AW, Artz N, Beswick AD, et al. Improving patients' experience and outcome of total joint replacement: the RESTORE programme. NIHR Journals Library. Southampton (UK), 2016.

38. Peer MA, Rush R, Gallacher PD, et al. Pre-surgery exercise and post-operative physical function of people undergoing knee replacement surgery: A systematic review and meta-analysis of randomized controlled trials. J Rehabil Med 2017;**49**(4):304-15.

39. Jordan RW, Smith NA, Chahal GS, et al. Enhanced education and physiotherapy before knee replacement; is it worth it? A systematic review. Physiotherapy 2014;**100**(4):305-12.

40. Minns Lowe CJ, Barker KL, Dewey M, et al. Effectiveness of physiotherapy exercise after knee arthroplasty for osteoarthritis: systematic review and meta-analysis of randomised controlled trials. BMJ 2007;**335**(7624):812.

41. Beswick AD, Wylde V, Gooberman-Hill R. Interventions for the prediction and management of chronic postsurgical pain after total knee replacement: systematic review of randomised controlled trials. BMJ Open 2015;**5**(5):e007387.

42. Wylde V, Beswick A, Dennis J, et al. Post-operative patient-related risk factors for chronic pain after total knee replacement: a systematic review. BMJ Open In press.

For peer review only

For peer review only - http://bmjopen.bmj.com/site/about/guidelines.xhtml

43. Barker KL, Beard D, Price A, et al. COmmunity-based Rehabilitation after Knee Arthroplasty (CORKA): study protocol for a randomised controlled trial. Trials 2016;**17**(1):501.

44. Haroutiunian S, Nikolajsen L, Finnerup NB, et al. The neuropathic component in persistent postsurgical pain: A systematic literature review. Pain 2013;**154**(1):95-102.

45. Howells N, Murray J, Wylde V, et al. Persistent pain after knee replacement: do factors associated with pain vary with degree of patient dissatisfaction? Osteoarthritis Cartilage 2016;**24**(12):2061-68.

46. Mandalia V, Eyres K, Schranz P, et al. Evaluation of patients with a painful total knee replacement. J Bone Joint Surg Br 2008;**90**(3):265-71.

47. Toms AD, Mandalia V, Haigh R, et al. The management of patients with painful total knee replacement. J Bone Joint Surg Br 2009;**91**(2):143-50.

48. van Bussel CM, Stronks DL, Huygen FJ. Complex regional pain syndrome type I of the knee: a systematic literature review. Eur J Pain 2014;**18**(6):766-73.

49. Vissers MM, Bussmann JB, Verhaar JA, et al. Psychological factors affecting the outcome of total hip and knee arthroplasty: a systematic review. Semin Arthritis Rheum 2012;**41**(4):576-88.

50. Turk DC, Wilson HD, Cahana A. Treatment of chronic non-cancer pain. Lancet 2011;**377**(9784):2226-35.

51. Lavand'homme P, Thienpont E. Pain after total knee arthroplasty: a narrative review focusing on the stratification of patients at risk for persistent pain. Bone Joint J 2015;**97-B**(10 Suppl A):45-8.

52. Artz N, Elvers KT, Lowe CM, et al. Effectiveness of physiotherapy exercise following total knee replacement: systematic review and meta-analysis. BMC musculoskeletal disorders 2015;**16**:15.

For peer review only

For peer review only - http://bmjopen.bmj.com/site/about/guidelines.xhtml

PRISMA 2009 Checklist

Section/topic	#	Checklist item	Reported on page #
**TITLE**			
Title	1	Identify the report as a systematic review, meta-analysis, or both.	1
**ABSTRACT**			
Structured summary	2	Provide a structured summary including, as applicable: background; objectives; data sources; study eligibility criteria, participants, and interventions; study appraisal and synthesis methods; results; limitations; conclusions and implications of key findings; systematic review registration number.	2-3
**INTRODUCTION**			
Rationale	3	Describe the rationale for the review in the context of what is already known.	4
Objectives	4	Provide an explicit statement of questions being addressed with reference to participants, interventions, comparisons, outcomes, and study design (PICOS).	4-5
**METHODS**			
Protocol and registration	5	Indicate if a review protocol exists, if and where it can be accessed (e.g., Web address), and, if available, provide registration information including registration number.	5
Eligibility criteria	6	Specify study characteristics (e.g., PICOS, length of follow-up) and report characteristics (e.g., years considered, language, publication status) used as criteria for eligibility, giving rationale.	5-6
Information sources	7	Describe all information sources (e.g., databases with dates of coverage, contact with study authors to identify additional studies) in the search and date last searched.	6
Search	8	Present full electronic search strategy for at least one database, including any limits used, such that it could be repeated.	Appendix 2
Study selection	9	State the process for selecting studies (i.e., screening, eligibility, included in systematic review, and, if applicable, included in the meta-analysis).	6-7
Data collection process	10	Describe method of data extraction from reports (e.g., piloted forms, independently, in duplicate) and any processes for obtaining and confirming data from investigators.	6-7
Data items	11	List and define all variables for which data were sought (e.g., PICOS, funding sources) and any assumptions and simplifications made.	6-7
Risk of bias in individual studies	12	Describe methods used for assessing risk of bias of individual studies (including specification of whether this was done at the study or outcome level), and how this information is to be used in any data synthesis.	7
Summary measures	13	State the principal summary measures (e.g., risk ratio, difference in means).	7
Synthesis of results	14	Describe the methods of handling data and combining results of studies, if done, including measures of consistency (e.g., I^2) for each meta-analysis.	7

For peer review only

PRISMA 2009 Checklist

Section/topic	#	Checklist item	Reported on page #
Risk of bias across studies	15	Specify any assessment of risk of bias that may affect the cumulative evidence (e.g., publication bias, selective reporting within studies).	7
Additional analyses	16	Describe methods of additional analyses (e.g., sensitivity or subgroup analyses, meta-regression), if done, indicating which were pre-specified.	7
**RESULTS**			
Study selection	17	Give numbers of studies screened, assessed for eligibility, and included in the review, with reasons for exclusions at each stage, ideally with a flow diagram.	Figure 1
Study characteristics	18	For each study, present characteristics for which data were extracted (e.g., study size, PICOS, follow-up period) and provide the citations.	Table 1
Risk of bias within studies	19	Present data on risk of bias of each study and, if available, any outcome level assessment (see item 12).	Table 2
Results of individual studies	20	For all outcomes considered (benefits or harms), present, for each study: (a) simple summary data for each intervention group (b) effect estimates and confidence intervals, ideally with a forest plot.	Figure 3, Appendix 3 and 4
Synthesis of results	21	Present results of each meta-analysis done, including confidence intervals and measures of consistency.	Figure 3
Risk of bias across studies	22	Present results of any assessment of risk of bias across studies (see Item 15).	Table 2
Additional analysis	23	Give results of additional analyses, if done (e.g., sensitivity or subgroup analyses, meta-regression [see Item 16]).	Appendix 5
**DISCUSSION**			
Summary of evidence	24	Summarize the main findings including the strength of evidence for each main outcome; consider their relevance to key groups (e.g., healthcare providers, users, and policy makers).	12-15
Limitations	25	Discuss limitations at study and outcome level (e.g., risk of bias), and at review-level (e.g., incomplete retrieval of identified research, reporting bias).	12-13
Conclusions	26	Provide a general interpretation of the results in the context of other evidence, and implications for future research.	13-15
**FUNDING**			
Funding	27	Describe sources of funding for the systematic review and other support (e.g., supply of data); role of funders for the systematic review.	16

For peer review only

Appendix 2: Search terms

MEDLINE (Ovid) (1946 to 15 November 2016)

1 randomized controlled trial/ or randomized controlled trial.pt.

2 controlled clinical trial.pt.

3 randomized.ab.

4 placebo.ab.

5 randomly.ab

6 trial.ab

7 randomised.tw

8 1 or 2 or 3 or 4 or 5 or 6 or 7

9 review/

10 'systematic review$'.mp

11 9 or 10

12 8 or 11

13 Arthroplasty, Replacement, Knee/

14 Knee Prosthesis/

15 (arthoplast$ adj3 knee$).mp. [mp=title, abstract, original title, name of substance word, subject heading word, keyword heading word, protocol supplementary concept word, rare disease supplementary concept word, unique identifier]

16 (knee$ adj3 replac$).mp. [mp=title, abstract, original title, name of substance word, subject heading word, keyword heading word, protocol supplementary concept word, rare disease supplementary concept word, unique identifier]

17 (knee adj3 implant$).mp. [mp=title, abstract, original title, name of substance word, subject heading word, keyword heading word, protocol supplementary concept word, rare disease supplementary concept word, unique identifier]

18 13 or 14 or 15 or 16 or 17

19 12 and 18

EMBASE (Ovid) (1980 to 15 November 2016)

1 Randomized controlled trial/ or Randomization/ or Single blind procedure/ or Double blind procedure/ or Crossover procedure/ or Placebo/ or Randomised controlled trial$.tw. or Randomized controlled trial$.tw. or RCT.tw. or Random allocation.tw. or Randomly allocated.tw. or Allocated randomly.tw. or (allocated adj2 random).tw. or Single blind$.tw. or Double blind$.tw. or ((treble or triple) adj blind$).tw. or Placebo$.tw.

2 "systematic review"/

3 meta analysis/

For peer review only - http://bmjopen.bmj.com/site/about/guidelines.xhtml

4 2 or 3

5 1 or 4

6 knee arthroplasty/

7 total knee arthroplasty/

8 knee prosthesis/

9 (knee$ adj3 arthoplast$).mp. [mp=title, abstract, heading word, drug trade name, original title, device manufacturer, drug manufacturer, device trade name, keyword, floating subheading]

10 (knee$ adj3 replac$).mp. [mp=title, abstract, heading word, drug trade name, original title, device manufacturer, drug manufacturer, device trade name, keyword, floating subheading]

11 (knee$ adj3 implant$).mp. [mp=title, abstract, heading word, drug trade name, original title, device manufacturer, drug manufacturer, device trade name, keyword, floating subheading]

12 6 or 7 or 8 or 9 or 10 or 11

13 5 and 12

PsycINFO (Ovid) (inception [1806] to 15 November 2016)

1. (knee$ adj3 arthoplast$).mp. [mp=title, abstract, heading word, table of contents, key concepts, original title, tests & measures]

2. (knee$ adj3 replac$).mp. [mp=title, abstract, heading word, table of contents, key concepts, original title, tests & measures]

3. (knee$ adj3 surg$).mp. [mp=title, abstract, heading word, table of contents, key concepts, original title, tests & measures]

4. (knee$ adj3 implant$).mp. [mp=title, abstract, heading word, table of contents, key concepts, original title, tests & measures]

5. (knee adj3 prosthe$).mp. [mp=title, abstract, heading word, table of contents, key concepts, original title, tests & measures]

6. 1 or 2 or 3 or 4 or 5

The Cochrane Library (Wiley) (inception to 15 November 2016)

#1 MeSH descriptor: [Knee Prosthesis] explode all trees

#2 MeSH descriptor: [Arthroplasty, Replacement, Knee] explode all trees

#3 arthoplast* N3 knee*

#4 knee* N3 replac*

#5 knee* N3 implant*

#6 #1 or #2 or #3 or #4 or #5

For peer review only

For peer review only - http://bmjopen.bmj.com/site/about/guidelines.xhtml

CINAHL (EBSCOHOST) (1982 to 15 November 2016)

S25	S15 AND S23 Limiters - Exclude MEDLINE records
S24	S15 AND S23
S23	S16 OR S17 OR S18 OR S19 OR S20 OR S21 OR S22
S22	knee* N3 implant*
S21	knee* N3 arthoplast*
S20	arthoplast* N3 knee*
S19	knee* N3 replac*
S18	"knee prosthes*"
S17	MH "Knee surgery"
S16	MH "Arthroplasty, Replacement, Knee"
S15	(S8 OR S14)
S14	(S9 OR S10 OR S11 OR S12 OR S13)
S13	metaanalyses
S12	metaanalysis
S11	meta-analyses
S10	meta-analysis
S9	systematic review
S8	S1 OR S2 OR S3 OR S4 OR S5 OR S6 OR S7
S7	AB trial$
S6	AB randomly
S5	AB randomised OR randomized
S4	(MH "clinical trials")
S3	clinical trials
S2	(MH "randomized controlled trials")
S1	randomized controlled trials

For peer review only - http://bmjopen.bmj.com/site/about/guidelines.xhtml

Appendix 3: Adverse event reporting and findings

Publication	Adverse events assessment/definition	Results summary
Bruun-Olsen et al, 2013 [1]	None	
Buhagiar et al, 2017 [2]	Postdischarge complication and adverse event data were collected until 1 year after surgery by self-report at follow-up visits and by a review of hospital electronic medical records.	Detailed breakdown of post-operative complications is provided in Table 3. There were no significant between-group differences in emergency department visits, readmissions and manipulations under anaesthetic.
Chen et al, 2016 [3]	None	
Fransen et al, 2017 [4]	Adverse events defined as event resulting in readmission to the hospital or resulting in a medical intervention or reduced function for 3 or more days.	Intervention: 1 death, 24 hospital admissions (15 TKR related), 20 other adverse events<br><br>Control: 1 death, 16 hospital admissions (13 TKR related), 17 other adverse events
Frost et al, 2002 [5]	None	
Kauppila et al, 2010 [6]	Not stated	No adverse events due to the intervention were reported
Ko et al, 2013 [7]	Postoperative adverse events were monitored via treating therapists and documented by the blinded assessor.	Detailed breakdown of post-operative adverse events provided in Appendix 1. Superficial wound infection, major infection, venous thrombotic embolism, neurovascular, TKR-related readmission and manipulation under anaesthetic similar between groups. No adverse events were associated with any of the treatment arms.
Kramer et al, 2003 [8]	None	
Liebs et al, 2010 [9]	Not stated	Prevalence of postoperative complications was similar between groups.
Liebs et al, 2012 [10]	Not stated	5 patients in early aquatic therapy group and 1 patient in the late aquatic therapy group admitted to hospital within 3 months of surgery.
Minns Lowe et al, 2012 [11]	Not stated	No adverse events
Moffet et al, 2004 [12]	Not stated	No adverse events
Monticone et al, 2013 [13]	None	
Petterson et al, 2009 [14]	Not stated	No adverse events were related to the exercise intervention. Only 1 patient reported feeling dizzy and lightheaded

For peer review only

		following the first NMES treatment.
Szots et al, 2016 [15]	None	
Vuorenmaa et al, 2014 [16]	Not stated	Intervention: 5 patients discontinued training due to pain (2 reported knee pain on operated side, 1 reported knee pain on the contralateral side, 1 reported back pain, and 1 reported hip pain), 1 patient had surgery due to reduced range of motion

For peer review only

Study	Results summary
Bruun-Olsen et al, 2013	No significant difference in mean KOOS pain scores (p-value not reported)<br>Intervention: 82 (SD 21)<br>Control: 74 (SD 23)
Buhagiar et al, 2017	No significant difference in median KOOS pain scores between groups (p-value not reported)<br>Intervention: 86 (IQR 74 to 97)<br>Control: 91 (IQR 78-98)
Chen et al, 2016	No significant difference in mean VAS pain scores (p-value not reported)<br>Intervention: 8.7 (SD 5.1)<br>Control: 9.3 (SD 5.5)
Fransen et al, 2017	No significant difference in mean WOMAC pain scores (p=0.71)<br>Intervention: 2.6 (SE 0.2)<br>Control: 2.5 (SE 0.2)
Frost et al, 2002	No significant difference in mean OKS item 'pain on walking' scores (p=0.68)<br>Intervention: 1.6 (SD 0.8)<br>Control: 1.5 (SD 0.93)
Kauppila et al, 2010	No significant difference in mean WOMAC pain scores (p=0.17)<br>Intervention: 23.5 (SD 22.3)<br>Control: 19.3 (SD 17.5)
Ko et al, 2013	No significant difference in mean WOMAC pain scores (p=0.79)<br>1:1 sessions: Median 3.8 (IQR 0.5-9.6)<br>Group sessions: Median 1.6 (IQR 0-7.5)<br>Home programme: Median 2.5 (IQR 0-9.5)
Kramer et al, 2003	No significant difference in mean WOMAC pain scores (p-value and mean pain scores not reported)
Liebs et al, 2010	No significant difference in mean WOMAC pain scores (p=0.454)<br>Intervention: 15.6 (SD 17.9)<br>Control: 13.0 (SD 14.9)
Liebs et al, 2012	No significant difference in mean WOMAC pain scores (p=0.334)<br>Intervention: 13.2 (SD 15.0)<br>Control: 17.4 (SD 22.4)
Minns Lowe et al, 2012	No statistical comparison of median KOOS pain scores (pilot study)<br>Intervention: 80.6 (IQ 36)<br>Control: 90.3 (IQ 33)
Moffet et al, 2004	No significant difference in mean WOMAC pain scores (p=0.161)<br>Intervention: 9.4 (SD 12.4)<br>Control: 11.8 (SD 13.0)
Monticone et al, 2013	Mean KOOS pain score significantly lower in intervention group (p<0.001)<br>Intervention: 87.35 (SD 11.71)<br>Control: 77.38 (SD 15.07)
Petterson et al,	No significant difference in mean KOS ADL item 'affect of pain on function' (p

For peer review only - http://bmjopen.bmj.com/site/about/guidelines.xhtml

Study	Results summary
2009	value not reported)<br>Intervention: 0.82 (SD not reported)<br>Control: 0.89 (SD not reported)
Szots et al, 2016	No significant difference in mean change in WOMAC pain scores (p=0.329)<br>Intervention: -25.9 (95% CI = -30.8, -21.0)<br>Control: -29.5 (95% CI = -35.2, -23.8)
Vuorenmaa et al, 2014	No significant difference in mean change in WOMAC pain scores (p=0.70)<br>Intervention: -15 (95% CI -20 to -10)<br>Control: -14 (95% CI -19 to -9)

For peer review only

	Physiotherapy			Usual care				Std. Mean Difference
Study or Subgroup	Mean	SD	Total	Mean	SD	Total	Weight	IV, Random, 95% CI
Bruun-Olsen 2013	-82	21	29	-74	23	28	20.7%	-0.36 [-0.88, 0.17]
Fransen 2017	2.6	2.6758	169	2.5	2.6	179	30.0%	0.04 [-0.17, 0.25]
Minns Lowe 2012	-77.49	18.96	49	-80.1	22.18	49	24.5%	0.13 [-0.27, 0.52]
Monticone 2013	-87.35	11.71	55	-77.38	15.07	55	24.8%	-0.73 [-1.12, -0.35]
**Total (95% CI)**			**302**			**311**	**100.0%**	**-0.21 [-0.61, 0.18]**

Heterogeneity: Tau² = 0.12; Chi² = 14.11, df = 3 (P = 0.003); I² = 79%
Test for overall effect: Z = 1.07 (P = 0.28)

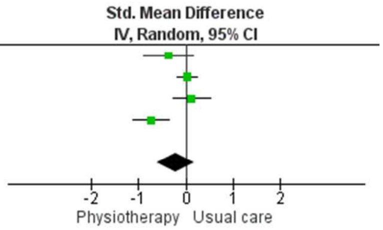

For peer review only

BMJ Open

The effectiveness of post-discharge intervention for reducing the severity of chronic pain after total knee replacement: Systematic review of randomised controlled trials

Journal:	*BMJ Open*
Manuscript ID	bmjopen-2017-020368.R1
Article Type:	Research
Date Submitted by the Author:	12-Dec-2017
Complete List of Authors:	Wylde, Vikki; University of Bristol, Musculoskeletal Research Unit<br>Dennis, Jane; University of Bristol, Musculoskeletal Research Unit<br>Gooberman-Hill, Rachael; University of Bristol, School of Clinical Sciences<br>Beswick, Andrew; University of Bristol, School of Clinical Sciences
**Primary Subject Heading**:	Rehabilitation medicine
Secondary Subject Heading:	Surgery, Health services research
Keywords:	Total knee replacement, Chronic post-surgical pain, Prevention, Systematic review

SCHOLARONE™
Manuscripts

For peer review only

The effectiveness of post-discharge intervention for reducing the severity of chronic pain after total knee replacement: Systematic review of randomised controlled trials

Wylde V[1,2], Dennis J[1], Gooberman-Hill R[1,2], Beswick AD[1]

[1]Musculoskeletal Research Unit, Translational Health Sciences, Bristol Medical School, University of Bristol, UK

[2]National Institute for Health Research Bristol Biomedical Research Centre, University of Bristol, UK

E-mail addresses

Vikki Wylde: v.wylde@bristol.ac.uk

Jane Dennis: jane.dennis@bristol.ac.uk

Rachael Gooberman-Hill: r.gooberman-hill@bristol.ac.uk

Andrew Beswick: andy.beswick@bristol.ac.uk

Corresponding author

Dr Vikki Wylde, Musculoskeletal Research Unit, Translational Health Sciences, Bristol Medical School, Learning and Research Building, Southmead Hospital, Bristol, BS10 5NB, UK

E-mail: V.Wylde@bristol.ac.uk, Telephone: +44 (0)117 414 7878

Word count: 2744

Running title: Post-discharge interventions and chronic pain after TKR

For peer review only - http://bmjopen.bmj.com/site/about/guidelines.xhtml

ABSTRACT

Objective

Approximately 20% of patients experience chronic pain after total knee replacement (TKR). The aim of this systematic review was to evaluate the effectiveness of post-discharge interventions commenced in the first three months after surgery in reducing the severity of chronic pain after TKR

Design

The protocol for this systematic review was registered on PROSPERO (CRD42017041382). MEDLINE, Embase, CINAHL, PsycINFO and *The Cochrane Library* were searched from inception to November 2016. Randomised controlled trials of post-discharge intervention which commenced in the first three months after TKR surgery were included. The primary outcome of the review was self-report pain severity at 12 months or longer after TKR. Risk of bias was assessed using the Cochrane risk-of-bias tool.

Results

Seventeen trials with data from 2,485 randomised participants were included. The majority of trials evaluated physiotherapy interventions (n=13); other interventions included nurse-led interventions (n=2), neuromuscular electrical stimulation (n=1) and a multidisciplinary intervention (n=1). Opportunities for meta-analysis were limited by heterogeneity. No study found a difference in long-term pain severity between trial arms, with the exception of one trial which found home-based functional exercises aimed at managing kinesiophobia resulted in lower pain severity scores at 12 months post-operative compared to advice to stay active.

Conclusion

For peer review only

For peer review only - http://bmjopen.bmj.com/site/about/guidelines.xhtml

This systematic review and narrative synthesis found no evidence that one type of physiotherapy intervention is more effective than another at reducing the severity of chronic pain after TKR. Further research is needed to evaluate non-physiotherapy interventions, including the provision of care as part of a stratified and multidisciplinary care package.

Key words: Total knee replacement, chronic post-surgical pain, prevention, systematic review

Strengths and limitations of this study

- This is the first systematic review to evaluate the effectiveness of post-discharge interventions delivered in the first three months after surgery in reducing the severity of chronic pain after total knee replacement.
- Synthesis of adverse events data was not possible because assessment and reporting was variable and often poor.
- We did not include studies that used a composite pain and function measure to assess pain outcome.

For peer review only

INTRODUCTION

Total knee replacement (TKR) is a common operation to provide pain relief, predominately due to osteoarthritis. Despite good outcomes for many, some patients report chronic pain in the months and years after TKR. Chronic post-surgical pain is defined as pain that is present or increases in intensity at ≥3 months after surgery [1]. In representative populations, unfavourable long-term pain outcomes have been reported by 10-34% of patients with TKR [2]. Patients with bothersome pain at ≥3 months after surgery are disappointed with their outcome [3 4]. Given the prevalence and impact of chronic pain, it is important to evaluate interventions that may optimise patients' outcomes after TKR.

During the hospital stay after TKR, rehabilitation focuses on regaining range of motion and improving mobility. After discharge, rehabilitation aims to enhance recovery, through supporting a person to regain function and quality of life, optimising pain relief and re-integration into social and personal environments [5]. While physiotherapy often focusses on functional health, another key outcome is the prevention of long-term pain [6]. Post-operative physiotherapy may be combined with other interventions to provide comprehensive, multidisciplinary and holistic rehabilitation [7]. A key step to improving patients' outcomes after TKR is to evaluate if early post-operative rehabilitation interventions can reduce the severity of chronic pain after TKR. Chronic pain is difficult to treat once established [8], and therefore it is important to evaluate the effectiveness of early post-operative interventions in reducing the severity of chronic pain.

The aim of this systematic review was to evaluate the effectiveness of post-discharge interventions delivered in the first three months after surgery for reducing the severity of chronic pain after TKR.

For peer review only - http://bmjopen.bmj.com/site/about/guidelines.xhtml

METHODS

The review was registered on the international prospective register of systematic reviews (PROSPERO) on 17th January 2017 (registration number CRD42017041382). The review was conducted following guidance from the Cochrane Handbook [9] and reported in accordance with PRISMA guidelines [10] (Appendix 1).

Eligibility criteria

Studies were eligible for inclusion in the review if they met the following criteria:

Population: Adults discharged from hospital after primary TKR predominantly for osteoarthritis.

Intervention: Any post-discharge intervention which commenced in the first three months after TKR surgery.

Control: Any, including no intervention, usual care, placebo or an alternative intervention.

Outcomes: The primary outcome was pain severity at 12 months or longer after TKR, as patient-reported levels of pain plateau by this time point [11 12]. Pain severity could be assessed using a patient-reported joint-specific pain measure (e.g. WOMAC or KOOS pain domains), a quality of life measure (e.g. SF-36 or SF-12) or a Visual Analogue Scale (VAS). The secondary outcome was adverse events.

Study type: Randomised controlled trials (RCTs).

Information sources and searches

MEDLINE, Embase, CINAHL, PsycINFO and *The Cochrane Library* were searched from inception to 15th November 2016 (Appendix 2). No language restrictions were applied and relevant non-English articles were translated and included if appropriate. Studies reported

For peer review only - http://bmjopen.bmj.com/site/about/guidelines.xhtml

only as abstracts or that were unobtainable as full text copies using inter-library loans or email contact with authors were excluded. Citations of key reviews and studies were checked in ISI Web of Science.

Screening

Records identified by searches were imported into Endnote X7 (Thomson Reuters) and duplicates removed. From the searches, an Endnote database of all RCTs and systematic reviews in TKR was established. Within this database, interventions conducted during the post-operative period were identified. An initial screen for potential eligibility was undertaken by one reviewer (ADB) to exclude articles that were clearly not relevant. Subsequently, abstracts and full-text articles were screened independently by two reviewers (VW and ADB or JD). Results of screening were compared, and any discrepancies were resolved through further review of the full text articles and discussion between reviewers. Reasons for exclusion were recorded.

Data extraction

Data from studies that met the eligibility criteria were extracted onto a standardised proforma by one reviewer (VW). Data extraction was checked against source articles by a second reviewer (JD). Extracted data comprised: country, date, participant characteristics, selection criteria; intervention and control treatment; follow up intervals; losses to follow-up; primary outcome; outcome data for pain; adverse events (any untoward medical occurrence in a clinical study participant regardless of the causal relationship with the study treatment) and information for risk of bias assessment. Any disagreements between reviewers were discussed with a third reviewer (ADB) and consensus reached.

A single e-mail was sent to authors of studies with an appropriate follow-up period but no pain outcome to enquire if an appropriate outcome was available. If a combined pain and

For peer review only - http://bmjopen.bmj.com/site/about/guidelines.xhtml

function outcome was reported, such as the OKS or the total WOMAC score, separate pain subscale data were requested. Authors were contacted when necessary for clarification purposes or to request unpublished relevant data. If a study reported data that were combined for knee and hip replacement patients, then disaggregated data for patients with TKR were requested. If this was not available, then the study was excluded.

Risk of bias assessment

Potential sources of bias were assessed using the Cochrane risk of bias tool [9]. At the protocol stage, analysis was planned which included all studies, with sensitivity analyses conducted to exclude studies judged to be at high risk of bias.

Strategy for data synthesis

At the protocol stage, meta-analysis using RevMan 5 [13] was planned if two or more studies were identified with similar interventions and comparator groups and appropriate outcome data. If continuous pain outcomes were measured differently across studies, overall standardised mean differences and 95% confidence intervals would be calculated and presented alongside measures of heterogeneity (I^2). Where possible, subgroup analyses were planned to explore the effectiveness of different intervention content and intensity, and different comparator interventions.

Opportunities for pooling outcome data in meta-analysis were limited by heterogeneity. This included the content, duration and intensity of both the treatments in both the intervention and comparison group. For example, a number of the trials were pragmatic with the control group receiving ‘usual care’, which varied considerably between studies. Therefore, a narrative synthesis was performed.

For peer review only - http://bmjopen.bmj.com/site/about/guidelines.xhtml

RESULTS

Searches identified 7,954 articles. After detailed evaluation of full-text articles, 17 studies with 2,485 randomised participants were included [14-30] (Figure 1). Two included studies were published after the search dates, but were identified from protocols published within the search dates [15 17].

Study characteristics

Table 1 provides an overview of study characteristics. Included studies were from Australia (n=3), Canada (n=2), Finland (n=2), Germany (n=2), United Kingdom (n=2), China (n=1), Denmark (n=1), Italy (n=1), Norway (n=1), Turkey (n=1) and United States of America (n=1). The number of centres was reported for 15 studies: eight studies were conducted in a single centre, three studies were conducted in two centres, and four studies were conducted in ≥4 centres. Sample sizes ranged from 34 to 422 participants, with a median of 117. All studies had two arms, with the exception of one three-arm trial [20]. Three studies were described as pilot or feasibility studies [16 18 24].

Study quality

Risk of bias assessments for individual studies are displayed in Figure 2. All studies were at high risk of bias for blinding of participants and pain outcome assessment due to the nature of the intervention and the self-reporting of pain. Five studies were at high risk of bias due to incomplete outcome data and one due to selective outcome reporting.

Outcomes assessment

The primary outcome was specified for 13 trials; this was function in eight trials; a composite of pain and function in four trials, and pain in one trial (Appendix 3). Pain severity was most commonly assessed using the WOMAC pain scale (n=9); other tools included the KOOS pain

For peer review only - http://bmjopen.bmj.com/site/about/guidelines.xhtml

scale (n=4), VAS (n=2), and single items from the OKS (n=1) and KOS ADL (n=1). Adverse events were poorly described and reported in the majority of studies and therefore pooling of harms data was not possible. A summary of adverse events findings is presented in Appendix 4. Pain was assessed at 12 months after TKR in 16 studies and at 14 months in one study. A summary of results from the individual studies is provided in Appendix 3.

Interventions

The majority of studies evaluated physiotherapy interventions (n=13); other interventions evaluated included nurse-led interventions (n=2), neuromuscular electrical stimulation (NMES) (n=1) and a multidisciplinary intervention (n=1).

Physiotherapy interventions

Thirteen studies with 1,880 randomised patients evaluated the effectiveness of post-discharge physiotherapy interventions. All interventions started within two months of surgery, with the majority commencing within two weeks of surgery. In addition to all studies being at risk of bias due to issues with blinding, risk of bias due to incomplete outcome data was evident for four studies [18 22 23 25]. Seven studies compared physiotherapy interventions with usual care or minimal care; interventions included a walking skills programme [14], group-based circuit exercise classes [17], erogometer cycling [22], home-based functional rehabilitation [24], clinic-based functional rehabilitation [25], home-based functional exercises aimed at managing kinesiophobia [26], and delayed monitored home exercises [29]. Five studies compare two forms of treatment including inpatient rehabilitation compared with home exercise [15], home-based functional exercise and home-based traditional exercise [18], 1:1 physiotherapy and home-based rehabilitation [21], supervised and home-based physiotherapy [30], early aquatic therapy and late aquatic therapy [23], and a three-arm trial comparing 1:1 physiotherapy, group-based circuit classes and a monitored home exercise programme [20].

For peer review only - http://bmjopen.bmj.com/site/about/guidelines.xhtml

For peer review only

Of the 13 studies, only one trial reported a difference in pain severity between groups; patients randomised to 6 months of home-based exercises aimed at managing kinesiophobia had lower pain severity scores at 12 months post-operative compared with patients who received general advice to stay active [26].

Nurse-led interventions

Two studies with 319 randomised patients reported evaluation of a nurse-led intervention compared with no care or usual care. Except for issues relating to blinding, both studies were at low risk of bias. Both studies evaluated nurse-led structured telephone follow-up; one aimed to improve adherence to home exercise [16] and the other to provide information regarding well-being, integrity, prophylaxis, safety and other issues relevant to patients after TKR [28]. Pain outcome data (mean and standard deviations) were not available for latter study and therefore meta-analysis was not possible. Neither study found a difference in pain severity scores at 12 months post-operative between the intervention and control group.

Other interventions

Two studies reported evaluations of other interventions. Except for issues relating to blinding, both studies were at low risk of bias. One trial involving 86 patients compared a group-based multidisciplinary programme with usual care [19]. This 10-day programme involved physiotherapy, Nordic walking, relaxation strategies, and sessions with a psychologist, social worker, nutritionist and orthopaedic surgeon. Another trial with 200 patients, at high risk of bias due to incomplete outcome data and selective outcome reporting, evaluated a combined NMES and volitional strength training programme compared with volitional strength training program without NMES [27]. Both studies found no difference in pain severity scores at 12 months post-operative between the intervention and control group.

For peer review only - http://bmjopen.bmj.com/site/about/guidelines.xhtml

Ongoing research

In searches of databases and citation searches on ISI Web of Science we identified a number of published RCT protocols that are evaluating post-discharge interventions with a pain severity outcome at ≥12 months after TKR. Interventions being evaluated include a digital activity coaching system for home exercise [31], Wii-enhanced rehabilitation [32], group-based outpatient physiotherapy with an individualised element [33], multicomponent rehabilitation for patients at risk of a poor outcome [34] and physiotherapy for patients performing poorly at six weeks after TKR [35]. Some of these studies are now finished and findings are likely to be reported imminently.

DISCUSSION

This systematic review aimed to evaluate the effectiveness of post-discharge interventions delivered in the first three months after surgery for reducing the severity of chronic pain after TKR. Interventions that predominately comprise physiotherapy have been evaluated in RCTs. In most studies, the control group received some form of physiotherapy care and therefore the aim of the trials was to compare the effectiveness of different types of physiotherapy, rather than comparing the effectiveness of physiotherapy to no care. A narrative synthesis of the evidence suggests that no physiotherapy intervention appears to be more effective than another at reducing the severity of chronic pain after TKR. However, findings from the trial of a 6 month home-based functional exercise programme aimed at managing kinesiophobia [26] compared to advice to stay active were encouraging and warrant further evaluation. Few studies have been conducted to evaluate the effectiveness of non-physiotherapy interventions at reducing chronic pain after TKR, and further research is needed.

For peer review only - http://bmjopen.bmj.com/site/about/guidelines.xhtml

For peer review only

There are a number of strengths and limitations to this systematic review. The main outcome of interest in this review was pain severity at ≥12 months after TKR. Although the primary outcome of many of the included studies was function, pain severity was an important secondary outcome in these studies. Studies that used a composite pain and function measure to assess outcome, for example the OKS or WOMAC, were excluded if authors were unable to provide pain subscales scores. Although this reduced the number of studies eligible for inclusion, this approach was taken because pain and function are distinct outcome domains, with different predictors and recovery trajectories [36 37]. The secondary outcome of this review was adverse events, to allow the synthesis of harms data. However, synthesis was not possible because assessment and reporting of adverse events was variable and often poor. The quality of adverse events reporting is a common issue in surgical trials [38], and evidence-based recommendations are needed to promote standardisation, improve quality and reduce heterogeneity of adverse events reporting in orthopaedic studies. A potential limitation of the included studies was that they were all at high risk of bias due to the lack of participant blinding for self-report pain. However, blinding of participants is rarely possible in RCTs of this nature. Also, it would be expected that the risk would arise from participants in the intervention group reporting less pain, which may potentially be an issue with shorter-term outcomes, but this was not evident from the longer-term follow-up of the studies included in this review.

This systematic review took a broad approach by evaluating the effectiveness of any type of post-discharge intervention that aimed to reduce the severity of chronic pain after TKR. Interventions that span the post-operative period may be delivered as part of a comprehensive peri-operative package of care, and these would not have been identified in this review; however, evaluations of the effectiveness of pre-operative and peri-operative interventions for reducing chronic pain severity are being conducted separately (CRD42017041382).

For peer review only - http://bmjopen.bmj.com/site/about/guidelines.xhtml

Previous systematic reviews of interventions to improve long-term outcomes after TKR have been conducted, but these have evaluated pre-operative interventions or have been narrower in focus. Systematic reviews of pre-operative interventions have found that exercise and education have a limited effect on improving long-term pain and function after TKR [39-43]. Previous systematic reviews of post-discharge interventions have focussed on physiotherapy, finding some evidence of short-term benefit but a lack of evidence to draw conclusions about long-term benefit [6 44 45]. One systematic review has evaluated interventions for the management of chronic pain after TKR, identifying only a single RCT of botulinum toxin A injections [46]. Our systematic review adds to this literature by providing evidence that no specific type of post-discharge physiotherapy intervention appears to be more effective than another at reducing the severity of chronic pain after TKR, although the positive impact of a home-based programme aimed at managing kinesiophobia compared with advice to stay active warrants further investigation.

The aim of this review was to evaluate the effectiveness of post-discharge interventions at reducing chronic pain severity after TKR. However, the primary aim of most trials included in the review was to improve functional ability after TKR. Only one trial had a primary outcome of pain severity [29], although a number of other trials assessed their primary outcome with a composite measure of pain and function [17 20 24 26]. However, pain severity was assessed as a secondary outcome in these trials and therefore it was expected that the intervention may reduce long-term pain. All but one study found that the intervention did not provide any benefit on long-term pain severity compared to the control group. However, the treatment received in the control group, particularly in the physiotherapy trials, varied considerably between studies, including a different form or intensity of physiotherapy, provision of physiotherapy based on a needs assessment, delayed treatment or no treatment. Therefore, it is not appropriate to draw conclusions on the effectiveness of any particular type

For peer review only - http://bmjopen.bmj.com/site/about/guidelines.xhtml

of physiotherapy intervention based on the findings of this review. However, the evidence does suggest that no type of physiotherapy intervention is more effective than another at reducing the severity of chronic pain after TKR. An important finding of this review is that only four trials have been conducted which have evaluated non-physiotherapy interventions, highlighting the need for more research in this field. In particular, further research with pain severity as the pain outcome is needed to ensure that RCTs are adequately powered to evaluate the effectiveness of post-discharge interventions on reducing chronic pain severity.

There are important considerations in the design and delivery of future post-discharge interventions that warrant further discussion. All the interventions included in this review were uniformly delivered to all patients, rather than just those patients who may be at most risk of poor outcome. Only 20% of patients will develop chronic pain after TKR [2] and delivering physiotherapy to all patients may reduce the ability to detect clinical benefit in terms of pain severity. In the future, interventions might be more effective if they include processes to identify patients at high risk of chronic pain and provide these patients with intensive and comprehensive interventions that have been specifically designed to reduce their risk of developing chronic pain. However, identifying high risk patients is challenging; pre-operative models to identify patients at risk of a poor outcome have low predictive power [36 37], and the evidence for post-operative risk factors is limited [47]. However, research is currently ongoing to evaluate whether providing a rehabilitation programme for patients at risk of a poor outcome [48] or are 'functioning poorly' at six weeks after TKR [35] can improve longer-term outcomes.

Preventing chronic pain after TKR is challenging because of the complexity of this pain condition. Although chronic pain after TKR is not yet fully understood, the aetiology of this pain is multifactorial, including surgical factors, complex regional pain syndrome, pain sensitisation, neuropathic pain and psychosocial factors [49-54]. Therefore, an intervention

For peer review only - http://bmjopen.bmj.com/site/about/guidelines.xhtml

comprising a single treatment modality may be insufficient to address and reduce the causes of pain for all patients. As with the treatment of other chronic pain conditions, this highlights the importance of focused, individualised and multidisciplinary treatment [8 55]. Such an approach is being evaluated in an ongoing RCT of a care pathway for patients with chronic pain after TKR (ISRCTN92545361). Therefore, further research is needed to evaluate the effectiveness of providing stratified and multidisciplinary care packages for preventing chronic pain after TKR.

In conclusion, findings from this systematic review and narrative synthesis are that there is no evidence that one type of physiotherapy intervention is more effective than another at reducing the severity of chronic pain after TKR. Further research is needed to evaluate non-physiotherapy interventions, including the provision of care as part of stratified and multidisciplinary care package.

For peer review only

For peer review only - http://bmjopen.bmj.com/site/about/guidelines.xhtml

FIGURE LEGENDS

Figure 1: Systematic review flow diagram

Figure 2: Risk of bias assessment for individual studies

For peer review only

For peer review only - http://bmjopen.bmj.com/site/about/guidelines.xhtml

ACKNOWLEDGEMENTS

We would like to thank all the study authors who took the time to reply to our requests for further clarification or additional data.

AUTHOR CONTRIBUTIONS

All authors contributed to the concept and design of the study. ADB, JD and VW contributed to the acquisition and analysis of data. VW drafted the article and ADB, JD and RGH revised it critically for important intellectual content. VW and ADB take responsibility for the integrity of the work as a whole, from inception to finished article.

FUNDING

This article presents independent research funded by the National Institute for Health Research (NIHR) under its Programme Grants for Applied Research programme (RP-PG-0613-20001). The views expressed are those of the authors and not necessarily those of the NHS, the NIHR or the Department of Health. The funder had no involvement in the study design, data collection, data analysis, interpretation of data or writing of the manuscript.

This study was supported by the NIHR Biomedical Research Centre at the University Hospitals Bristol NHS Foundation Trust and the University of Bristol. The views expressed in this publication are those of the author(s) and not necessarily those of the NHS, the National Institute for Health Research or the Department of Health.

COMPETING INTERESTS

The authors have no conflicts of interest to declare.

DATA SHARING STATEMENT

No additional data are available.

For peer review only

Table 1: Overview of study characteristics

Publication<br>Location<br>Date of study<br>Number of centres	Randomised<br>Mean age<br>% female	Intervention treatment	Control treatment	Pain assessment<br>Adherence to treatment<br>Losses to follow-up
Bruun-Olsen et al, 2013<br>Norway<br>2008-2010<br>2 centres	N=57(29:28)<br>68:69 years<br>62:50%	Group-based physiotherapist-led walking skills programme (2-6 patients per group). Commenced 6 weeks after surgery. 12 sessions over 6-8 weeks.	1:1 usual physiotherapy care consisting of 12 individual physiotherapy sessions. Commenced 6 weeks after surgery. Twice weekly sessions until 12-14 weeks after surgery.	KOOS pain scale<br>28/29 completed intervention<br>28/28 received control treatment<br>6 (2:4) lost to follow-up
Buhagiar et al, 2017<br>Australia<br>2012-2015<br>2 centres	N=165(81:84)<br>67:67 years<br>69:68%	Inpatient rehabilitation at rehabilitation facility with twice daily supervised sessions of 1:1 and group-based exercises. Commenced after hospital discharge for 10 days. Home exercise programme after discharge from rehabilitation facility.	Home exercise programme comprising of 2-3 group-based outpatient session to practice and progress exercises. Commenced 2 weeks after surgery.	KOOS pain scale<br>72/81 adhered to intervention<br>74/84 adhered to control treatment<br>6(2:4) lost to follow-up
Buker et al, 2014*<br>Turkey<br>2009-2011<br>1 centre	N=34 (18:16)<br>64:68 years<br>89:94%	20 sessions of supervised physiotherapy and rehabilitation including range of motion and strengthening exercises, application of heat and TENS application. Five days a week for four weeks.	Home exercises including range of motion and strengthening exercise for one hour per day. Five days a week for four weeks.	Pain VAS<br>Adherence not reported<br>Losses to follow-up not reported
Chen et al, 2016<br>China<br>2013-2014<br>1 centre	N=202(101:101)<br>66:67 years<br>63:67%	Structured telephone follow-up by nurse at 1, 3 and 6 weeks after hospital discharge to improve adherence to home exercise routine.	No telephone follow-up	Pain VAS<br>Adherence not reported<br>15(7:8) lost to follow-up
Fransen et al, 2017<br>Australia<br>2009-2012	N=422(212:210)<br>64:65 years<br>54:52%	Group-based circuit exercise classes supervised by physiotherapist. Up to 6 patients per class. Commenced 6 weeks after surgery. Twice	Usual physiotherapy care. 22% of participants reported 6 or more occasions of physiotherapy	WOMAC pain scale<br>140/212 participants attended ≥12 classes

For peer review only - http://bmjopen.bmj.com/site/about/guidelines.xhtml

12 centres		weekly sessions for at least 8 weeks.	during the 6–12-week period after TKR	210/210 received control treatment<br>74(43:31) lost to follow-up
Frost et al, 2002<br>UK<br>1995-1996<br>Not reported	N=47(23:24)<br>72:71 years<br>48:50%	Home-based functional exercise. Commenced following discharge from hospital. Duration not reported.	Home-based traditional exercise.	OKS item (pain on walking)<br>Adherence not reported<br>20 (7:13) lost to follow-up
Kauppila et al, 2010[$]<br>Finland<br>2002-2005<br>1 centre	N=86(44:42)<br>71:71 years<br>76:79%	Group-based multidisciplinary rehabilitation programme. Up to 8 patients per group. Commenced 2-4 months after surgery for 10 days.	Usual physiotherapy care. Supervised exercise programme at 2 month outpatient visit, with provision of further rehabilitation based on needs assessment.	WOMAC pain scale<br>44/44 attended intervention<br>42:42 received control treatment<br>11 (8:3) lost to follow-up
Ko et al, 2013<br>Australia<br>2008-2010<br>3 arm trial<br>4 centres	N=<br>249(85:84:80)<br>67:68:67 years<br>68:60:61%	1:1 physiotherapy with home-based sessions. Commenced 2 weeks after surgery. Twice weekly 1:1 and home-based sessions over 6 weeks.	1.) Group-based circuit classes supervised by physiotherapist with home-based sessions. Up to 8 patients per class. Commenced 2 weeks after surgery. Twice weekly group and home-based sessions over 6 weeks.<br><br>2.) Monitored home programme, 2 1:1 physiotherapy sessions and 1 telephone follow-up call. Commenced 2 weeks after surgery. 4 sessions per week for 6 weeks.	WOMAC pain scale<br>80% participants attended 9 or more 1:1 sessions, 77% attended 9 or more group sessions, 83% attended both sessions in monitored home programme group.<br>16(7:3:6) lost to follow-up
Kramer et al, 2003<br>Canada<br>Not reported<br>Not reported	N=160(80:80)<br>68:69 years<br>59:55%	1:1 clinic-based rehabilitation programme with home exercise programme. Commenced 2 weeks after surgery. Up to 2 sessions a week for 10 weeks.	Home-based rehabilitation, monitored by telephone calls from physiotherapist. Commenced 2 weeks after surgery. At least 1 telephone call in weeks 2-6 and 1 call in	WOMAC pain scale<br>76/80 received intervention<br>78/80 received control treatment<br>26 (15:22) lost to follow-up

For peer review only - http://bmjopen.bmj.com/site/about/guidelines.xhtml

			weeks 7-12.	
Liebs et al, 2010*<br>Germany<br>2005-2006<br>5 centres	N=159(85:74)<br>70:70 years<br>73:70%	Ergometer cycling supervised by physiotherapist. Commenced 2 weeks after surgery. 3 sessions a week for at least 3 weeks.	No ergometer cycling.	WOMAC pain scale<br>Adherence not reported<br>33(15:18) lost to follow-up
Liebs et al, 2012*<br>Germany<br>2003-2004<br>2 centres	N=185(87:98)<br>69:71 years<br>70:73%	Early aquatic therapy. Commenced 6 days after surgery. 3 times a week up to 5th week post-operative.	Late aquatic therapy. Commenced 14 days after surgery. 3 times a week up to 5th week post-operative.	WOMAC pain scale<br>Adherence not reported<br>41(18:23) lost to follow-up
Minns Lowe et al, 2012<br>UK<br>2006-2008<br>1 centre	N=107 (56:51)<br>68:71 years<br>57:59%	Home-based functional rehabilitation with 2 visits from physiotherapist at 2 weeks and 6-8 weeks after hospital discharge. Twice daily exercises for at least 3 months.	Usual physiotherapy care involving provision of an exercise booklet, with outpatient physiotherapy on a needs only basis. No additional home visits.	KOOS pain scale<br>46/56 patients received 2 visits<br>47/51 received control treatment<br>9 (7:2) lost to follow-up
Moffet et al, 2004 [25]<br>Canada<br>1997-1999<br>5 centres	N=77(38:39)<br>67:69 years<br>63:56%	Functional rehabilitation programme with individualised home exercises. Commenced at 2 months after surgery. 12 supervised sessions over 6-8 weeks.	Usual physiotherapy care, which included supervised home rehabilitation visits for 26% of patients.	WOMAC pain scale<br>38/38 participated in 12 sessions<br>39/39 received control treatment<br>8(0:8) lost to follow-up
Monticone et al, 2010-2013<br>Italy<br>1 centre	N=110(55:55)<br>67:68 years<br>65:62%	Home-based functional exercises aimed at managing kinesiophobia, with monthly phone calls to encourage adherence. Commenced after hospital discharge. Twice weekly sessions for 6 months.	No physiotherapy, advice to stay active.	KOOS pain scale<br>Adherence not reported<br>0 lost to follow-up
Petterson et al, 2009<br>USA<br>200-2005<br>1 centre	N=200(100:100)<br>65:65 years<br>47:45%	Combined neuromuscular electric stimulation (NMES) and volitional strength training programme. Commenced 3-4 weeks after surgery. 2 or 3 sessions a week for 6 weeks.	Volitional strength training program without NMES.	KOS ADL item (effect of pain on function)<br>84/100 completed intervention<br>97/100 completed control treatment<br>51 (32:19) lost to follow-up
Szots et al, 2016<br>Denmark	N=117(59:58)<br>67:68 years	Two nurse-led structured telephone follow-up calls. Telephone calls at 4 days and 14 days	No telephone follow-up.	WOMAC pain scale<br>54/59 patients had both telephone

For peer review only - http://bmjopen.bmj.com/site/about/guidelines.xhtml

2013<br>1 centre	61:67%	after hospital discharge.		follow-up calls<br>54/58 received control treatment<br>9(5:4) lost to follow-up
Vuorenmaa et al, 2014 **<br>Finland<br>2008-2010<br>1 centre	N=108(53:55)<br>69:69 years<br>57:65%	Delayed monitored home exercises, with guidance from physiotherapist at 2, 3 and 6 months post-operative. Commenced at 2 months after surgery for 12 months.	Usual care, which involved no additional guidance from 2 months post-operative.	WOMAC pain scale<br>72% of patients performed the training sessions at least twice per week in the first 6 months<br>53/53 received control treatment<br>4(2:2) lost to follow-up

* 24 month follow-up also conducted but data from 12 month follow-up included in table to be consistent with follow-up period of other studies

** Follow-up at 14 months post-operative

$ Pain-specific outcome data was provided by the authors for a previous review [56] and was used again in this review

For peer review only

For peer review only - http://bmjopen.bmj.com/site/about/guidelines.xhtml

REFERENCES

1. Werner MU, Kongsgaard UE. I. Defining persistent post-surgical pain: is an update required? Br J Anaesth 2014;**113**(1):1-4.

2. Beswick AD, Wylde V, Gooberman-Hill R, et al. What proportion of patients report long-term pain after total hip or knee replacement for osteoarthritis? A systematic review of prospective studies in unselected patients. BMJ Open 2012;**2**(1):e000435.

3. Scott CE, Howie CR, MacDonald D, et al. Predicting dissatisfaction following total knee replacement: a prospective study of 1217 patients. J Bone Joint Surg Br 2010;**92**(9):1253-8.

4. Jeffery AE, Wylde V, Blom AW, et al. "It's there and I'm stuck with it": patients' experiences of chronic pain following total knee replacement surgery. Arthritis Care Res (Hoboken) 2011;**63**(2):286-92.

5. Artz N, Dixon S, Wylde V, et al. Physiotherapy provision following discharge after total hip and total knee replacement: a survey of current practice at high-volume NHS hospitals in England and wales. Musculoskeletal Care 2013;**11**(1):31-8.

6. Artz N, Elvers K, Minns Lowe C, et al. Effectiveness of physiotherapy exercise following total knee replacement: systematic review and meta-analysis. BMC Musculoskeletal Disorders 2015;**16**(15).

7. Khan F, Ng L, Gonzalez S, et al. Multidisciplinary rehabilitation programmes following joint replacement at the hip and knee in chronic arthropathy. Cochrane Database Syst Rev 2008(2):CD004957.

8. Turk DC, Wilson HD, Cahana A. Treatment of chronic non-cancer pain. Lancet 2011;**377**(9784):2226-35.

9. Higgins J, Green SE. Cochrane Handbook for Systematic Reviews of Interventions Version 5.1.0 [updated March 2011]. The Cochrane Collaboration, 2011. Available from www.cochrane-handbook.org, 2011.

10. Moher D, Liberati A, Tetzlaff J, et al. Preferred reporting items for systematic reviews and meta-analyses: the PRISMA statement. BMJ 2009;**339**:b2535.

11. Lenguerrand E, Wylde V, Gooberman-Hill R, et al. Trajectories of Pain and Function after Primary Hip and Knee Arthroplasty: The ADAPT Cohort Study. PLoS One 2016;**11**(2):e0149306.

For peer review only - http://bmjopen.bmj.com/site/about/guidelines.xhtml

12. Kennedy DM, Stratford PW, Hanna SE, et al. Modeling early recovery of physical function following hip and knee arthroplasty. BMC Musculoskelet Disord 2006;**7**:100.

13. Review Manager. (RevMan) [Computer program]. Version 5.3. Copenhagen: The Nordic Cochrane Centre, The Cochrane Collaboration. 2014.

14. Bruun-Olsen V, Heiberg KE, Wahl AK, et al. The immediate and long-term effects of a walking-skill program compared to usual physiotherapy care in patients who have undergone total knee arthroplasty (TKA): a randomized controlled trial. Disability and rehabilitation 2013;**35**(23):2008-15.

15. Buhagiar MA, Naylor JM, Harris IA, et al. Effect of Inpatient Rehabilitation vs a Monitored Home-Based Program on Mobility in Patients With Total Knee Arthroplasty: The HIHO Randomized Clinical Trial. JAMA 2017;**317**(10):1037-46.

16. Chen M, Li P, Lin F. Influence of structured telephone follow-up on patient compliance with rehabilitation after total knee arthroplasty. Patient Preference and Adherence 2016;**10**:257-64.

17. Fransen M, Nairn L, Bridgett L, et al. Post-Acute Rehabilitation After Total Knee Replacement: A Multicenter Randomized Clinical Trial Comparing Long-Term Outcomes. Arthritis Care Res (Hoboken) 2017;**69**(2):192-200.

18. Frost H, Lamb SE, Robertson S. A randomized controlled trial of exercise to improve mobility and function after elective knee arthroplasty. Feasibility, results and methodological difficulties. Clinical rehabilitation 2002;**16**(2):200-9.

19. Kauppila AM, Kyllonen E, Ohtonen P, et al. Multidisciplinary rehabilitation after primary total knee arthroplasty: a randomized controlled study of its effects on functional capacity and quality of life. Clinical rehabilitation 2010;**24**(5):398-411.

20. Ko V, Naylor J, Harris I, et al. One-to-one therapy is not superior to group or home-based therapy after total knee arthroplasty: a randomized, superiority trial. The Journal of bone and joint surgery. American volume 2013;**95**(21):1942-9.

21. Kramer JF, Speechley M, Bourne R, et al. Comparison of clinic- and home-based rehabilitation programs after total knee arthroplasty. Clinical orthopaedics and related research 2003(410):225-34.

22. Liebs TR, Herzberg W, Ruther W, et al. Ergometer cycling after hip or knee replacement surgery: a randomized controlled trial. The Journal of bone and joint surgery. American volume 2010;**92**(4):814-22.

For peer review only - http://bmjopen.bmj.com/site/about/guidelines.xhtml

23. Liebs T, Herzberg W, Rüther W, et al. Multicenter randomized controlled trial comparing early versus late aquatic therapy after total hip or knee arthroplasty. Archives of physical medicine and rehabilitation 2012; 93(2). http://onlinelibrary.wiley.com/o/cochrane/clcentral/articles/334/CN-00832334/frame.html

http://ac.els-cdn.com/S0003999311008434/1-s2.0-S0003999311008434-main.pdf?_tid=41aa00ce-f508-11e6-b06b-00000aab0f27&acdnat=1487332829_31eb2562ac20096dc09cc02ed9cf1f7e.

24. Minns Lowe CJ, Barker KL, Holder R, et al. Comparison of postdischarge physiotherapy versus usual care following primary total knee arthroplasty for osteoarthritis: an exploratory pilot randomized clinical trial. Clinical rehabilitation 2012;**26**(7):629-41.

25. Moffet H, Collet JP, Shapiro SH, et al. Effectiveness of intensive rehabilitation on functional ability and quality of life after first total knee arthroplasty: A single-blind randomized controlled trial. Archives of physical medicine and rehabilitation 2004;**85**(4):546-56.

26. Monticone M, Ferrante S, Rocca B, et al. Home-based functional exercises aimed at managing kinesiophobia contribute to improving disability and quality of life of patients undergoing total knee arthroplasty: a randomized controlled trial. Archives of physical medicine and rehabilitation 2013;**94**(2):231-9.

27. Petterson SC, Mizner RL, Stevens JE, et al. Improved function from progressive strengthening interventions after total knee arthroplasty: a randomized clinical trial with an imbedded prospective cohort. Arthritis and rheumatism 2009;**61**(2):174-83.

28. Szöts K, Konradsen H, Solgaard S, et al. Long-term effects of telephone follow-up after total knee arthroplasty. Journal of Nursing Education and Practice 2016;**6**(7):151-54.

29. Vuorenmaa M, Ylinen J, Piitulainen K, et al. Efficacy of a 12-month, monitored home exercise programme compared with normal care commencing 2 months after total knee arthroplasty: a randomized controlled trial. Journal of rehabilitation medicine 2014;**46**(2):166-72.

30. Buker N, Akkaya S, Akkaya N, et al. Comparison of effects of supervised physiotherapy and a standardized home program on functional status in patients with total knee arthroplasty: a prospective study. J Phys Ther Sci 2014;**26**(10):1531-6.

31. Harmelink KEM, Zeegers A, Tonis TM, et al. The effectiveness of the use of a digital activity coaching system in addition to a two-week home-based exercise program in

For peer review only - http://bmjopen.bmj.com/site/about/guidelines.xhtml

patients after total knee arthroplasty: study protocol for a randomized controlled trial. BMC Musculoskelet Disord 2017;**18**(1):290.

32. Negus JJ, Cawthorne DP, Chen JS, et al. Patient outcomes using Wii-enhanced rehabilitation after total knee replacement - the TKR-POWER study. Contemp Clin Trials 2015;**40**:47-53.
33. Wylde V, Artz N, Marques E, et al. Effectiveness and cost-effectiveness of outpatient physiotherapy after knee replacement for osteoarthritis: study protocol for a randomised controlled trial. Trials 2016;**17**(1):289.
34. Barker KL, Beard D, Price A, et al. COmmunity-based Rehabilitation after Knee Arthroplasty (CORKA): Study protocol for a randomised controlled trial. Trials 2016;**17**(1):no pagination.
35. Simpson AH, Hamilton DF, Beard DJ, et al. Targeted rehabilitation to improve outcome after total knee replacement (TRIO): study protocol for a randomised controlled trial. Trials 2014;**15**:44.
36. Judge A, Arden NK, Cooper C, et al. Predictors of outcomes of total knee replacement surgery. Rheumatology (Oxford) 2012;**51**(10):1804-13.
37. Lopez-Olivo MA, Landon GC, Siff SJ, et al. Psychosocial determinants of outcomes in knee replacement. Ann Rheum Dis 2011;**70**(10):1775-81.
38. Wylde V, Dennis J, Beswick AD, et al. Systematic review of management of chronic pain after surgery. British Journal of Surgery 2017;**104**(10):1293-306.
39. Blom AW, Artz N, Beswick AD, et al. Improving patients' experience and outcome of total joint replacement: the RESTORE programme. NIHR Journals Library. Southampton (UK), 2016.
40. Peer MA, Rush R, Gallacher PD, et al. Pre-surgery exercise and post-operative physical function of people undergoing knee replacement surgery: A systematic review and meta-analysis of randomized controlled trials. J Rehabil Med 2017;**49**(4):304-15.
41. Jordan RW, Smith NA, Chahal GS, et al. Enhanced education and physiotherapy before knee replacement; is it worth it? A systematic review. Physiotherapy 2014;**100**(4):305-12.
42. Wang L, Lee M, Zhang Z, et al. Does preoperative rehabilitation for patients planning to undergo joint replacement surgery improve outcomes? A systematic review and meta-analysis of randomised controlled trials. BMJ Open 2016;**6**(2):e009857.

For peer review only

For peer review only - http://bmjopen.bmj.com/site/about/guidelines.xhtml

43. Chesham RA, Shanmugam S. Does preoperative physiotherapy improve postoperative, patient-based outcomes in older adults who have undergone total knee arthroplasty? A systematic review. Physiother Theory Pract 2017;**33**(1):9-30.

44. Minns Lowe CJ, Barker KL, Dewey M, et al. Effectiveness of physiotherapy exercise after knee arthroplasty for osteoarthritis: systematic review and meta-analysis of randomised controlled trials. BMJ 2007;**335**(7624):812.

45. Florez-Garcia M, Garcia-Perez F, Curbelo R, et al. Efficacy and safety of home-based exercises versus individualized supervised outpatient physical therapy programs after total knee arthroplasty: a systematic review and meta-analysis. Knee Surg Sports Traumatol Arthrosc 2017;**25**(11):3340-53.

46. Beswick AD, Wylde V, Gooberman-Hill R. Interventions for the prediction and management of chronic postsurgical pain after total knee replacement: systematic review of randomised controlled trials. BMJ Open 2015;**5**(5):e007387.

47. Wylde V, Beswick A, Dennis J, et al. Post-operative patient-related risk factors for chronic pain after total knee replacement: a systematic review. BMJ Open In press.

48. Barker KL, Beard D, Price A, et al. COmmunity-based Rehabilitation after Knee Arthroplasty (CORKA): study protocol for a randomised controlled trial. Trials 2016;**17**(1):501.

49. Haroutiunian S, Nikolajsen L, Finnerup NB, et al. The neuropathic component in persistent postsurgical pain: A systematic literature review. Pain 2013;**154**(1):95-102.

50. Howells N, Murray J, Wylde V, et al. Persistent pain after knee replacement: do factors associated with pain vary with degree of patient dissatisfaction? Osteoarthritis Cartilage 2016;**24**(12):2061-68.

51. Mandalia V, Eyres K, Schranz P, et al. Evaluation of patients with a painful total knee replacement. J Bone Joint Surg Br 2008;**90**(3):265-71.

52. Toms AD, Mandalia V, Haigh R, et al. The management of patients with painful total knee replacement. J Bone Joint Surg Br 2009;**91**(2):143-50.

53. van Bussel CM, Stronks DL, Huygen FJ. Complex regional pain syndrome type I of the knee: a systematic literature review. Eur J Pain 2014;**18**(6):766-73.

54. Vissers MM, Bussmann JB, Verhaar JA, et al. Psychological factors affecting the outcome of total hip and knee arthroplasty: a systematic review. Semin Arthritis Rheum 2012;**41**(4):576-88.

For peer review only - http://bmjopen.bmj.com/site/about/guidelines.xhtml

55. Lavand'homme P, Thienpont E. Pain after total knee arthroplasty: a narrative review focusing on the stratification of patients at risk for persistent pain. Bone Joint J 2015;**97-B**(10 Suppl A):45-8.

56. Artz N, Elvers KT, Lowe CM, et al. Effectiveness of physiotherapy exercise following total knee replacement: systematic review and meta-analysis. BMC musculoskeletal disorders 2015;**16**:15.

For peer review only

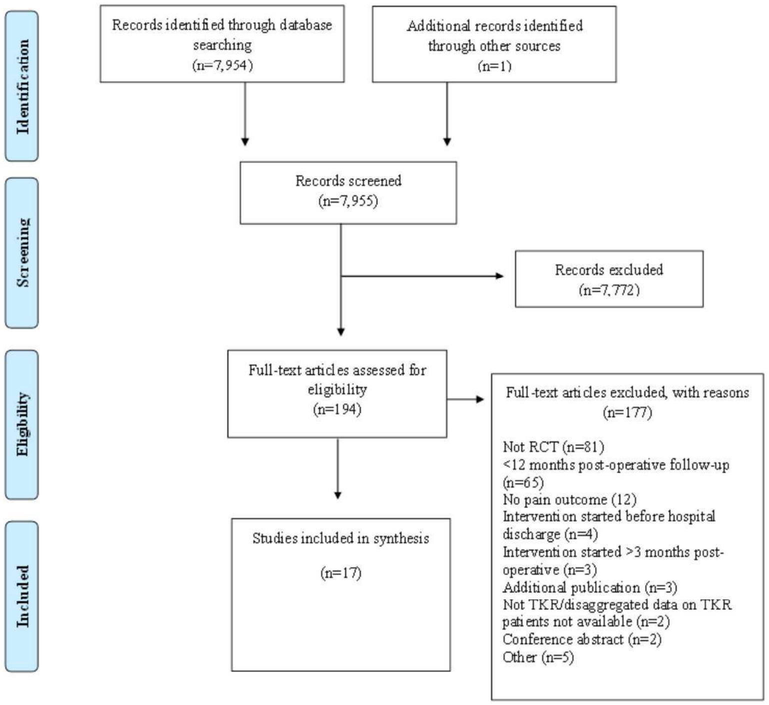

Figure 1: Systematic review flow diagram

254x190mm (300 x 300 DPI)

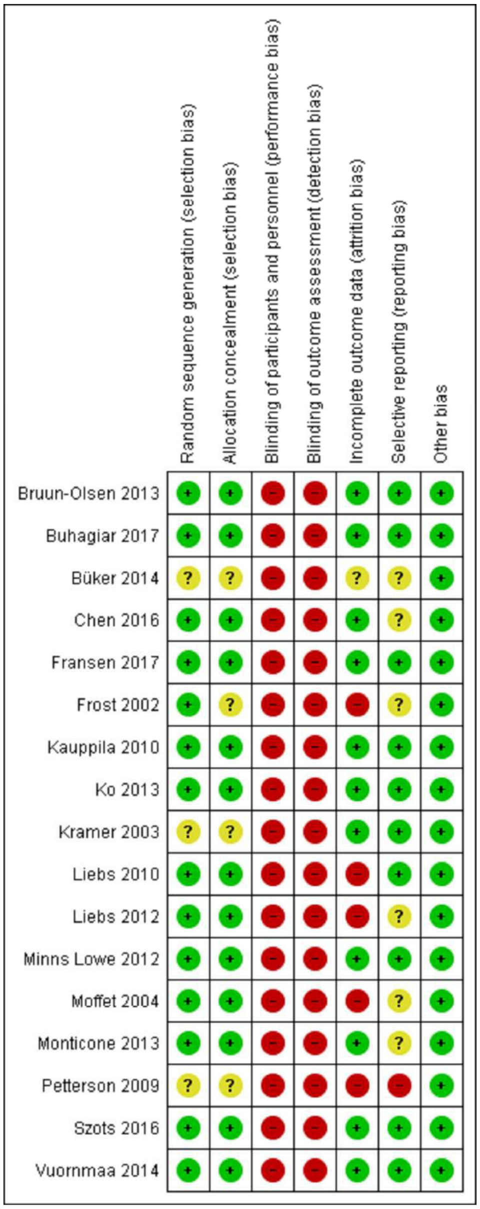

Figure 2: Risk of bias assessment for individual studies

211x535mm (300 x 300 DPI)

Appendix 1: PRISMA checklist

Section/topic	#	Checklist item	Reported on page #
**TITLE**			
Title	1	Identify the report as a systematic review, meta-analysis, or both.	1
**ABSTRACT**			
Structured summary	2	Provide a structured summary including, as applicable: background; objectives; data sources; study eligibility criteria, participants, and interventions; study appraisal and synthesis methods; results; limitations; conclusions and implications of key findings; systematic review registration number.	2-3
**INTRODUCTION**			
Rationale	3	Describe the rationale for the review in the context of what is already known.	4
Objectives	4	Provide an explicit statement of questions being addressed with reference to participants, interventions, comparisons, outcomes, and study design (PICOS).	4-5
**METHODS**			
Protocol and registration	5	Indicate if a review protocol exists, if and where it can be accessed (e.g., Web address), and, if available, provide registration information including registration number.	5
Eligibility criteria	6	Specify study characteristics (e.g., PICOS, length of follow-up) and report characteristics (e.g., years considered, language, publication status) used as criteria for eligibility, giving rationale.	5-6
Information sources	7	Describe all information sources (e.g., databases with dates of coverage, contact with study authors to identify additional studies) in the search and date last searched.	6
Search	8	Present full electronic search strategy for at least one database, including any limits used, such that it could be repeated.	Appendix 2
Study selection	9	State the process for selecting studies (i.e., screening, eligibility, included in systematic review, and, if applicable, included in the meta-analysis).	6-7
Data collection process	10	Describe method of data extraction from reports (e.g., piloted forms, independently, in duplicate) and any processes for obtaining and confirming data from investigators.	6-7
Data items	11	List and define all variables for which data were sought (e.g., PICOS, funding sources) and any assumptions and simplifications made.	6-7
Risk of bias in individual studies	12	Describe methods used for assessing risk of bias of individual studies (including specification of whether this was done at the study or outcome level), and how this information is to be used in any data synthesis.	7
Summary measures	13	State the principal summary measures (e.g., risk ratio, difference in means).	7
Synthesis of results	14	Describe the methods of handling data and combining results of studies, if done, including measures of consistency (e.g., I^2) for each meta-analysis.	7

For peer review only

Appendix 1: PRISMA checklist

Section/topic	#	Checklist item	Reported on page #
Risk of bias across studies	15	Specify any assessment of risk of bias that may affect the cumulative evidence (e.g., publication bias, selective reporting within studies).	7
Additional analyses	16	Describe methods of additional analyses (e.g., sensitivity or subgroup analyses, meta-regression), if done, indicating which were pre-specified.	7
**RESULTS**			
Study selection	17	Give numbers of studies screened, assessed for eligibility, and included in the review, with reasons for exclusions at each stage, ideally with a flow diagram.	Figure 1
Study characteristics	18	For each study, present characteristics for which data were extracted (e.g., study size, PICOS, follow-up period) and provide the citations.	Table 1
Risk of bias within studies	19	Present data on risk of bias of each study and, if available, any outcome level assessment (see item 12).	Table 2
Results of individual studies	20	For all outcomes considered (benefits or harms), present, for each study: (a) simple summary data for each intervention group (b) effect estimates and confidence intervals, ideally with a forest plot.	Figure 3, Appendix 3 and 4
Synthesis of results	21	Present results of each meta-analysis done, including confidence intervals and measures of consistency.	N/A
Risk of bias across studies	22	Present results of any assessment of risk of bias across studies (see Item 15).	Table 2
Additional analysis	23	Give results of additional analyses, if done (e.g., sensitivity or subgroup analyses, meta-regression [see Item 16]).	N/A
**DISCUSSION**			
Summary of evidence	24	Summarize the main findings including the strength of evidence for each main outcome; consider their relevance to key groups (e.g., healthcare providers, users, and policy makers).	12-15
Limitations	25	Discuss limitations at study and outcome level (e.g., risk of bias), and at review-level (e.g., incomplete retrieval of identified research, reporting bias).	12-13
Conclusions	26	Provide a general interpretation of the results in the context of other evidence, and implications for future research.	13-15
**FUNDING**			
Funding	27	Describe sources of funding for the systematic review and other support (e.g., supply of data); role of funders for the systematic review.	16

For peer review only

Appendix 2: Search terms

MEDLINE (Ovid) (1946 to 15 November 2016)

1 randomized controlled trial/ or randomized controlled trial.pt.

2 controlled clinical trial.pt.

3 randomized.ab.

4 placebo.ab.

5 randomly.ab

6 trial.ab

7 randomised.tw

8 1 or 2 or 3 or 4 or 5 or 6 or 7

9 review/

10 'systematic review$'.mp

11 9 or 10

12 8 or 11

13 Arthroplasty, Replacement, Knee/

14 Knee Prosthesis/

15 (arthoplast$ adj3 knee$).mp. [mp=title, abstract, original title, name of substance word, subject heading word, keyword heading word, protocol supplementary concept word, rare disease supplementary concept word, unique identifier]

16 (knee$ adj3 replac$).mp. [mp=title, abstract, original title, name of substance word, subject heading word, keyword heading word, protocol supplementary concept word, rare disease supplementary concept word, unique identifier]

17 (knee adj3 implant$).mp. [mp=title, abstract, original title, name of substance word, subject heading word, keyword heading word, protocol supplementary concept word, rare disease supplementary concept word, unique identifier]

18 13 or 14 or 15 or 16 or 17

19 12 and 18

EMBASE (Ovid) (1980 to 15 November 2016)

1 Randomized controlled trial/ or Randomization/ or Single blind procedure/ or Double blind procedure/ or Crossover procedure/ or Placebo/ or Randomised controlled trial$.tw. or Randomized controlled trial$.tw. or RCT.tw. or Random allocation.tw. or Randomly allocated.tw. or Allocated randomly.tw. or (allocated adj2 random).tw. or Single blind$.tw. or Double blind$.tw. or ((treble or triple) adj blind$).tw. or Placebo$.tw.

2 "systematic review"/

3 meta analysis/

For peer review only - http://bmjopen.bmj.com/site/about/guidelines.xhtml

4 2 or 3

5 1 or 4

6 knee arthroplasty/

7 total knee arthroplasty/

8 knee prosthesis/

9 (knee$ adj3 arthoplast$).mp. [mp=title, abstract, heading word, drug trade name, original title, device manufacturer, drug manufacturer, device trade name, keyword, floating subheading]

10 (knee$ adj3 replac$).mp. [mp=title, abstract, heading word, drug trade name, original title, device manufacturer, drug manufacturer, device trade name, keyword, floating subheading]

11 (knee$ adj3 implant$).mp. [mp=title, abstract, heading word, drug trade name, original title, device manufacturer, drug manufacturer, device trade name, keyword, floating subheading]

12 6 or 7 or 8 or 9 or 10 or 11

13 5 and 12

PsycINFO (Ovid) (inception [1806] to 15 November 2016)

1. (knee$ adj3 arthoplast$).mp. [mp=title, abstract, heading word, table of contents, key concepts, original title, tests & measures]

2. (knee$ adj3 replac$).mp. [mp=title, abstract, heading word, table of contents, key concepts, original title, tests & measures]

3. (knee$ adj3 surg$).mp. [mp=title, abstract, heading word, table of contents, key concepts, original title, tests & measures]

4. (knee$ adj3 implant$).mp. [mp=title, abstract, heading word, table of contents, key concepts, original title, tests & measures]

5. (knee adj3 prosthe$).mp. [mp=title, abstract, heading word, table of contents, key concepts, original title, tests & measures]

6. 1 or 2 or 3 or 4 or 5

The Cochrane Library (Wiley) (inception to 15 November 2016)

#1 MeSH descriptor: [Knee Prosthesis] explode all trees

#2 MeSH descriptor: [Arthroplasty, Replacement, Knee] explode all trees

#3 arthoplast* N3 knee*

#4 knee* N3 replac*

#5 knee* N3 implant*

#6 #1 or #2 or #3 or #4 or #5

For peer review only - http://bmjopen.bmj.com/site/about/guidelines.xhtml

CINAHL (EBSCOHOST) (1982 to 15 November 2016)

S25	S15 AND S23 Limiters - Exclude MEDLINE records
S24	S15 AND S23
S23	S16 OR S17 OR S18 OR S19 OR S20 OR S21 OR S22
S22	knee* N3 implant*
S21	knee* N3 arthoplast*
S20	arthoplast* N3 knee*
S19	knee* N3 replac*
S18	"knee prosthes*"
S17	MH "Knee surgery"
S16	MH "Arthroplasty, Replacement, Knee"
S15	(S8 OR S14)
S14	(S9 OR S10 OR S11 OR S12 OR S13)
S13	metaanalyses
S12	metaanalysis
S11	meta-analyses
S10	meta-analysis
S9	systematic review
S8	S1 OR S2 OR S3 OR S4 OR S5 OR S6 OR S7
S7	AB trial$
S6	AB randomly
S5	AB randomised OR randomized
S4	(MH "clinical trials")
S3	clinical trials
S2	(MH "randomized controlled trials")
S1	randomized controlled trials

For peer review only

Appendix 3: Trial results summary

Study	Primary aim of the study	Primary outcome (and measure)	Results summary
Buker et al, 2014	Determine the functional differences between patients who were treated with supervised physiotherapy or a standardized home program and perform a cost analysis.	Not specified	No significant difference in mean pain VAS scores (p-value not reported)<br><br>Rest pain<br>Intervention: 0.44 (SD 0.51)<br>Control: 0.37 (SD 0.80)<br><br>Activity pain<br>Intervention: 3.11 (SD 1.96)<br>Control: 2.50 (SD 1.77)
Bruun-Olsen et al, 2013	To examine the immediate and long-term effects of a walking-skill program compared with usual physiotherapy care.	Walking distance (6 minute walk test)	No significant difference in mean KOOS pain scores (p-value not reported)<br><br>Intervention: 82 (SD 21)<br>Control: 74 (SD 23)
Buhagiar et al, 2017	To determine if 10 days of inpatient rehabilitation followed by a monitored home-based program provided greater improvements than a monitored home-based program alone.	Walking distance (6 minute walk test)	No significant difference in median KOOS pain scores between groups (p-value not reported)<br><br>Intervention: 86 (IQR 74 to 97)<br>Control: 91 (IQR 78-98)
Chen et al, 2016	To assess the impact of structured telephone reinforcement on patient compliance with home exercises.	Not specified (pilot study)	No significant difference in mean VAS pain scores (p-value not reported)<br><br>Intervention: 8.7 (SD 5.1)<br>Control: 9.3 (SD 5.5)
Fransen et al, 2017	To evaluate the long-term benefit of providing a post-acute, class-based outpatient exercise program compared	Pain and function (WOMAC pain and function scales)	No significant difference in mean WOMAC pain scores (p=0.71)<br><br>Intervention: 2.6 (SE 0.2)

For peer review only

Appendix 3: Trial results summary

Study	Primary aim of the study	Primary outcome (and measure)	Results summary
	with current usual rehabilitation care.		Control: 2.5 (SE 0.2)
Frost et al, 2002	To assess the feasibility of comparing traditional exercise regimes with a more functional and dynamic approach.	Not specified (feasibility study)	No significant difference in mean OKS item 'pain on walking' scores (p=0.68)<br>Intervention: 1.6 (SD 0.8)<br>Control: 1.5 (SD 0.93)
Kauppila et al, 2010	To examine whether a multidisciplinary rehabilitation programme can improve functional recovery and quality of life and reduce the use of rehabilitation services compared with conventional care.	Function (WOMAC function scale)	No significant difference in mean WOMAC pain scores (p=0.17)<br>Intervention: 23.5 (SD 22.3)<br>Control: 19.3 (SD 17.5)
Ko et al, 2013	To determine whether center-based, one-to-one physical therapy provides superior outcomes compared with group-based therapy or a simple monitored home-based program.	Pain and function (Oxford Knee Score)	No significant difference in mean WOMAC pain scores (p=0.79)<br>1:1 sessions: Median 3.8 (IQR 0.5-9.6)<br>Group sessions: Median 1.6 (IQR 0-7.5)<br>Home programme: Median 2.5 (IQR 0-9.5)
Kramer et al, 2003	To compare clinic-based rehabilitation delivered in outpatient physical therapy clinics and home-based rehabilitation monitored by a physical therapist via periodic telephone calls.	Not specified	No significant difference in mean WOMAC pain scores (p-value and mean pain scores not reported)
Liebs et al, 2010	Evaluate the effect of ergometer cycling on health-related quality of life and patient satisfaction.	Function (WOMAC function scale)	No significant difference in mean WOMAC pain scores (p=0.454)<br>Intervention: 15.6 (SD 17.9)<br>Control: 13.0 (SD 14.9)
Liebs et al, 2012	To evaluate if the timing of aquatic	Function (WOMAC function scale)	No significant difference in mean

For peer review only

Appendix 3: Trial results summary

Study	Primary aim of the study	Primary outcome (and measure)	Results summary
	therapy influences clinical outcomes.		WOMAC pain scores (p=0.334)<br>Intervention: 13.2 (SD 15.0)<br>Control: 17.4 (SD 22.4)
Minns Lowe et al, 2012	To evaluate a pilot trial of a postdischarge physiotherapy intervention to improve patient function versus usual physiotherapy.	Pain and function (Oxford Knee Score)	No statistical comparison of median KOOS pain scores (pilot study)<br>Intervention: 80.6 (IQ 36)<br>Control: 90.3 (IQ 33)
Moffet et al, 2004	To evaluate the effectiveness of a new intensive functional rehabilitation program on functional ability and quality of life.	Walking distance (6 minute walk test)	No significant difference in mean WOMAC pain scores (p=0.161)<br>Intervention: 9.4 (SD 12.4)<br>Control: 11.8 (SD 13.0)
Monticone et al, 2013	To compare the improvement in disability, kinesiophobia, pain, and quality of life obtained by means of home-based functional exercises aimed at managing kinesiophobia with advice to stay active after discharge from a rehabilitation unit.	Pain and function (KOOS)	Mean KOOS pain score significantly lower in intervention group (p<0.001)<br>Intervention: 87.35 (SD 11.71)<br>Control: 77.38 (SD 15.07)
Petterson et al, 2009	To determine the effectiveness of progressive quadriceps strengthening with or without neuromuscular electrical stimulation (NMES).	Quadriceps strength and volitional muscle activation (burst superimposition technique)	No significant difference in mean KOS ADL item 'affect of pain on function' (p value not reported)<br>Intervention: 0.82 (SD not reported)<br>Control: 0.89 (SD not reported)
Szots et al, 2016	To evaluate the effects of structured nurse-managed telephone follow-up.	Function (WOMAC function scale)	No significant difference in mean change in WOMAC pain scores (p=0.329)<br>Intervention: -25.9 (95% CI = -30.8, -

For peer review only

Appendix 3: Trial results summary

Study	Primary aim of the study	Primary outcome (and measure)	Results summary
			21.0)<br>Control: -29.5 (95% CI = -35.2, -23.8)
Vuorenmaa et al, 2014	To evaluate the efficacy of a delayed home exercise programme compared with normal care.	Pain (WOMAC pain scale)	No significant difference in mean change in WOMAC pain scores (p=0.70)<br>Intervention: -15 (95% CI -20 to -10)<br>Control: -14 (95% CI -19 to -9)

For peer review only

Appendix 4: Adverse event reporting and findings

Publication	Adverse events assessment/definition	Results summary
Buker et al, 2014	None	
Bruun-Olsen et al, 2013	None	
Buhagiar et al, 2017	Postdischarge complication and adverse event data were collected until 1 year after surgery by self-report at follow-up visits and by a review of hospital electronic medical records.	Detailed breakdown of post-operative complications is provided in Table 3. There were no significant between-group differences in emergency department visits, readmissions and manipulations under anaesthetic.
Chen et al, 2016	None	
Fransen et al, 2017	Adverse events defined as event resulting in readmission to the hospital or resulting in a medical intervention or reduced function for 3 or more days.	Intervention: 1 death, 24 hospital admissions (15 TKR related), 20 other adverse events<br><br>Control: 1 death, 16 hospital admissions (13 TKR related), 17 other adverse events
Frost et al, 2002	None	
Kauppila et al, 2010	Not stated	No adverse events due to the intervention were reported
Ko et al, 2013	Postoperative adverse events were monitored via treating therapists and documented by the blinded assessor.	Detailed breakdown of post-operative adverse events provided in Appendix 1. Superficial wound infection, major infection, venous thrombotic embolism, neurovascular, TKR-related readmission and manipulation under anaesthetic similar between groups. No adverse events were associated with any of the treatment arms.
Kramer et al, 2003	None	
Liebs et al, 2010	Not stated	Prevalence of postoperative complications was similar between groups.
Liebs et al, 2012	Not stated	5 patients in early aquatic therapy group and 1 patient in the late aquatic therapy group admitted to hospital within 3 months of surgery.
Minns Lowe et al, 2012	Not stated	No adverse events
Moffet et al, 2004	Not stated	No adverse events
Monticone et al, 2013	None	
Petterson et al, 2009	Not stated	No adverse events were related to the

For peer review only - http://bmjopen.bmj.com/site/about/guidelines.xhtml

		exercise intervention. Only 1 patient reported feeling dizzy and lightheaded following the first NMES treatment.
Szots et al, 2016	None	
Vuorenmaa et al, 2014	Not stated	Intervention: 5 patients discontinued training due to pain (2 reported knee pain on operated side, 1 reported knee pain on the contralateral side, 1 reported back pain, and 1 reported hip pain), 1 patient had surgery due to reduced range of motion

For peer review only

BMJ Open

The effectiveness of post-discharge intervention for reducing the severity of chronic pain after total knee replacement: Systematic review of randomised controlled trials

Journal:	*BMJ Open*
Manuscript ID	bmjopen-2017-020368.R2
Article Type:	Research
Date Submitted by the Author:	16-Jan-2018
Complete List of Authors:	Wylde, Vikki; University of Bristol, Musculoskeletal Research Unit<br>Dennis, Jane; University of Bristol, Musculoskeletal Research Unit<br>Gooberman-Hill, Rachael; University of Bristol, School of Clinical Sciences<br>Beswick, Andrew; University of Bristol, School of Clinical Sciences
**Primary Subject Heading**:	Rehabilitation medicine
Secondary Subject Heading:	Surgery, Health services research
Keywords:	Total knee replacement, Chronic post-surgical pain, Prevention, Systematic review

SCHOLARONE™
Manuscripts

For peer review only

The effectiveness of post-discharge intervention for reducing the severity of chronic pain after total knee replacement: Systematic review of randomised controlled trials

Wylde V[1,2], Dennis J[1], Gooberman-Hill R[1,2], Beswick AD[1]

[1]Musculoskeletal Research Unit, Translational Health Sciences, Bristol Medical School, University of Bristol, UK

[2]National Institute for Health Research Bristol Biomedical Research Centre, University of Bristol, UK

E-mail addresses

Vikki Wylde: v.wylde@bristol.ac.uk

Jane Dennis: jane.dennis@bristol.ac.uk

Rachael Gooberman-Hill: r.gooberman-hill@bristol.ac.uk

Andrew Beswick: andy.beswick@bristol.ac.uk

Corresponding author

Dr Vikki Wylde, Musculoskeletal Research Unit, Translational Health Sciences, Bristol Medical School, Learning and Research Building, Southmead Hospital, Bristol, BS10 5NB, UK

E-mail: V.Wylde@bristol.ac.uk, Telephone: +44 (0)117 414 7878

Word count: 2744

Running title: Post-discharge interventions and chronic pain after TKR

For peer review only

For peer review only - http://bmjopen.bmj.com/site/about/guidelines.xhtml

ABSTRACT

Objective

Approximately 20% of patients experience chronic pain after total knee replacement (TKR). The aim of this systematic review was to evaluate the effectiveness of post-discharge interventions commenced in the first three months after surgery in reducing the severity of chronic pain after TKR

Design

The protocol for this systematic review was registered on PROSPERO (CRD42017041382). MEDLINE, Embase, CINAHL, PsycINFO and *The Cochrane Library* were searched from inception to November 2016. Randomised controlled trials of post-discharge intervention which commenced in the first three months after TKR surgery were included. The primary outcome of the review was self-report pain severity at 12 months or longer after TKR. Risk of bias was assessed using the Cochrane risk-of-bias tool.

Results

Seventeen trials with data from 2,485 randomised participants were included. The majority of trials evaluated physiotherapy interventions (n=13); other interventions included nurse-led interventions (n=2), neuromuscular electrical stimulation (n=1) and a multidisciplinary intervention (n=1). Opportunities for meta-analysis were limited by heterogeneity. No study found a difference in long-term pain severity between trial arms, with the exception of one trial which found home-based functional exercises aimed at managing kinesiophobia resulted in lower pain severity scores at 12 months post-operative compared to advice to stay active.

Conclusion

For peer review only - http://bmjopen.bmj.com/site/about/guidelines.xhtml

This systematic review and narrative synthesis found no evidence that one type of physiotherapy intervention is more effective than another at reducing the severity of chronic pain after TKR. Further research is needed to evaluate non-physiotherapy interventions, including the provision of care as part of a stratified and multidisciplinary care package.

Key words: Total knee replacement, chronic post-surgical pain, prevention, systematic review

Strengths and limitations of this study

- This is the first systematic review to evaluate the effectiveness of post-discharge interventions delivered in the first three months after surgery in reducing the severity of chronic pain after total knee replacement.
- Synthesis of adverse events data was not possible because assessment and reporting was variable and often poor.
- We did not include studies that used a composite pain and function measure to assess pain outcome.

For peer review only

For peer review only - http://bmjopen.bmj.com/site/about/guidelines.xhtml

INTRODUCTION

Total knee replacement (TKR) is a common operation to provide pain relief, predominately due to osteoarthritis. Despite good outcomes for many, some patients report chronic pain in the months and years after TKR. Chronic post-surgical pain is defined as pain that is present or increases in intensity at ≥3 months after surgery [1]. In representative populations, unfavourable long-term pain outcomes have been reported by 10-34% of patients with TKR [2]. Patients with bothersome pain at ≥3 months after surgery are disappointed with their outcome [3 4]. Given the prevalence and impact of chronic pain, it is important to evaluate interventions that may optimise patients' outcomes after TKR.

During the hospital stay after TKR, rehabilitation focuses on regaining range of motion and improving mobility. After discharge, rehabilitation aims to enhance recovery, through supporting a person to regain function and quality of life, optimising pain relief and re-integration into social and personal environments [5]. While physiotherapy often focusses on functional health, another key outcome is the prevention of long-term pain [6]. Post-operative physiotherapy may be combined with other interventions to provide comprehensive, multidisciplinary and holistic rehabilitation [7]. A key step to improving patients' outcomes after TKR is to evaluate if early post-operative rehabilitation interventions can reduce the severity of chronic pain after TKR. Chronic pain is difficult to treat once established [8], and therefore it is important to evaluate the effectiveness of early post-operative interventions in reducing the severity of chronic pain.

The aim of this systematic review was to evaluate the effectiveness of post-discharge interventions delivered in the first three months after surgery for reducing the severity of chronic pain after TKR.

For peer review only

For peer review only - http://bmjopen.bmj.com/site/about/guidelines.xhtml

METHODS

The review was registered on the international prospective register of systematic reviews (PROSPERO) on 17th January 2017 (registration number CRD42017041382). The review was conducted following guidance from the Cochrane Handbook [9] and reported in accordance with PRISMA guidelines [10] (Appendix 1).

Eligibility criteria

Studies were eligible for inclusion in the review if they met the following criteria:

Population: Adults discharged from hospital after primary TKR predominantly for osteoarthritis.

Intervention: Any post-discharge intervention which commenced in the first three months after TKR surgery.

Control: Any, including no intervention, usual care, placebo or an alternative intervention.

Outcomes: The primary outcome was pain severity at 12 months or longer after TKR, as patient-reported levels of pain plateau by this time point [11 12]. Pain severity could be assessed using a patient-reported joint-specific pain measure (e.g. WOMAC or KOOS pain domains), a quality of life measure (e.g. SF-36 or SF-12) or a Visual Analogue Scale (VAS). The secondary outcome was adverse events.

Study type: Randomised controlled trials (RCTs).

Information sources and searches

MEDLINE, Embase, CINAHL, PsycINFO and *The Cochrane Library* were searched from inception to 15th November 2016 (Appendix 2). No language restrictions were applied and relevant non-English articles were translated and included if appropriate. Studies reported

For peer review only - http://bmjopen.bmj.com/site/about/guidelines.xhtml

only as abstracts or that were unobtainable as full text copies using inter-library loans or email contact with authors were excluded. Citations of key reviews and studies were checked in ISI Web of Science.

Screening

Records identified by searches were imported into Endnote X7 (Thomson Reuters) and duplicates removed. From the searches, an Endnote database of all RCTs and systematic reviews in TKR was established. Within this database, interventions conducted during the post-operative period were identified. An initial screen for potential eligibility was undertaken by one reviewer (ADB) to exclude articles that were clearly not relevant. Subsequently, abstracts and full-text articles were screened independently by two reviewers (VW and ADB or JD). Results of screening were compared, and any discrepancies were resolved through further review of the full text articles and discussion between reviewers. Reasons for exclusion were recorded.

Data extraction

Data from studies that met the eligibility criteria were extracted onto a standardised proforma by one reviewer (VW). Data extraction was checked against source articles by a second reviewer (JD). Extracted data comprised: country, date, participant characteristics, selection criteria; intervention and control treatment; follow up intervals; losses to follow-up; primary outcome; outcome data for pain; adverse events (any untoward medical occurrence in a clinical study participant regardless of the causal relationship with the study treatment) and information for risk of bias assessment. Any disagreements between reviewers were discussed with a third reviewer (ADB) and consensus reached.

A single e-mail was sent to authors of studies with an appropriate follow-up period but no pain outcome to enquire if an appropriate outcome was available. If a combined pain and

For peer review only - http://bmjopen.bmj.com/site/about/guidelines.xhtml

function outcome was reported, such as the OKS or the total WOMAC score, separate pain subscale data were requested. Authors were contacted when necessary for clarification purposes or to request unpublished relevant data. If a study reported data that were combined for knee and hip replacement patients, then disaggregated data for patients with TKR were requested. If this was not available, then the study was excluded.

Risk of bias assessment

Potential sources of bias were assessed using the Cochrane risk of bias tool [9]. At the protocol stage, analysis was planned which included all studies, with sensitivity analyses conducted to exclude studies judged to be at high risk of bias.

Strategy for data synthesis

At the protocol stage, meta-analysis using RevMan 5 [13] was planned if two or more studies were identified with similar interventions and comparator groups and appropriate outcome data. If continuous pain outcomes were measured differently across studies, overall standardised mean differences and 95% confidence intervals would be calculated and presented alongside measures of heterogeneity (I^2). Where possible, subgroup analyses were planned to explore the effectiveness of different intervention content and intensity, and different comparator interventions.

Opportunities for pooling outcome data in meta-analysis were limited by heterogeneity. This included the content, duration and intensity of both the treatments in both the intervention and comparison group. For example, a number of the trials were pragmatic with the control group receiving ‘usual care’, which varied considerably between studies. Therefore, a narrative synthesis was performed.

For peer review only

For peer review only - http://bmjopen.bmj.com/site/about/guidelines.xhtml

RESULTS

Searches identified 7,954 articles. After detailed evaluation of full-text articles, 17 studies with 2,485 randomised participants were included [14-30] (Figure 1). Two included studies were published after the search dates, but were identified from protocols published within the search dates [15 17].

Study characteristics

Table 1 provides an overview of study characteristics. Included studies were from Australia (n=3), Canada (n=2), Finland (n=2), Germany (n=2), United Kingdom (n=2), China (n=1), Denmark (n=1), Italy (n=1), Norway (n=1), Turkey (n=1) and United States of America (n=1). The number of centres was reported for 15 studies: eight studies were conducted in a single centre, three studies were conducted in two centres, and four studies were conducted in ≥4 centres. Sample sizes ranged from 34 to 422 participants, with a median of 117. All studies had two arms, with the exception of one three-arm trial [20]. Three studies were described as pilot or feasibility studies [16 18 24].

Study quality

Risk of bias assessments for individual studies are displayed in Figure 2. All studies were at high risk of bias for blinding of participants and pain outcome assessment due to the nature of the intervention and the self-reporting of pain. Five studies were at high risk of bias due to incomplete outcome data and one due to selective outcome reporting.

Outcomes assessment

The primary outcome was specified for 13 trials; this was function in eight trials; a composite of pain and function in four trials, and pain in one trial (Appendix 3). Pain severity was most commonly assessed using the WOMAC pain scale (n=9); other tools included the KOOS pain

For peer review only - http://bmjopen.bmj.com/site/about/guidelines.xhtml

scale (n=4), VAS (n=2), and single items from the OKS (n=1) and KOS ADL (n=1). Adverse events were poorly described and reported in the majority of studies and therefore pooling of harms data was not possible. A summary of adverse events findings is presented in Appendix 4. Pain was assessed at 12 months after TKR in 16 studies and at 14 months in one study. A summary of results from the individual studies is provided in Appendix 3.

Interventions

The majority of studies evaluated physiotherapy interventions (n=13); other interventions evaluated included nurse-led interventions (n=2), neuromuscular electrical stimulation (NMES) (n=1) and a multidisciplinary intervention (n=1).

Physiotherapy interventions

Thirteen studies with 1,880 randomised patients evaluated the effectiveness of post-discharge physiotherapy interventions. All interventions started within two months of surgery, with the majority commencing within two weeks of surgery. In addition to all studies being at risk of bias due to issues with blinding, risk of bias due to incomplete outcome data was evident for four studies [18 22 23 25]. Seven studies compared physiotherapy interventions with usual care or minimal care; interventions included a walking skills programme [14], group-based circuit exercise classes [17], erogometer cycling [22], home-based functional rehabilitation [24], clinic-based functional rehabilitation [25], home-based functional exercises aimed at managing kinesiophobia [26], and delayed monitored home exercises [29]. Five studies compare two forms of treatment including inpatient rehabilitation compared with home exercise [15], home-based functional exercise and home-based traditional exercise [18], 1:1 physiotherapy and home-based rehabilitation [21], supervised and home-based physiotherapy [30], early aquatic therapy and late aquatic therapy [23], and a three-arm trial comparing 1:1 physiotherapy, group-based circuit classes and a monitored home exercise programme [20].

For peer review only - http://bmjopen.bmj.com/site/about/guidelines.xhtml

For peer review only

Of the 13 studies, only one trial reported a difference in pain severity between groups; patients randomised to 6 months of home-based exercises aimed at managing kinesiophobia had lower pain severity scores at 12 months post-operative compared with patients who received general advice to stay active [26].

Nurse-led interventions

Two studies with 319 randomised patients reported evaluation of a nurse-led intervention compared with no care or usual care. Except for issues relating to blinding, both studies were at low risk of bias. Both studies evaluated nurse-led structured telephone follow-up; one aimed to improve adherence to home exercise [16] and the other to provide information regarding well-being, integrity, prophylaxis, safety and other issues relevant to patients after TKR [28]. Pain outcome data (mean and standard deviations) were not available for latter study and therefore meta-analysis was not possible. Neither study found a difference in pain severity scores at 12 months post-operative between the intervention and control group.

Other interventions

Two studies reported evaluations of other interventions. Except for issues relating to blinding, both studies were at low risk of bias. One trial involving 86 patients compared a group-based multidisciplinary programme with usual care [19]. This 10-day programme involved physiotherapy, Nordic walking, relaxation strategies, and sessions with a psychologist, social worker, nutritionist and orthopaedic surgeon. Another trial with 200 patients, at high risk of bias due to incomplete outcome data and selective outcome reporting, evaluated a combined NMES and volitional strength training programme compared with volitional strength training program without NMES [27]. Both studies found no difference in pain severity scores at 12 months post-operative between the intervention and control group.

For peer review only - http://bmjopen.bmj.com/site/about/guidelines.xhtml

Ongoing research

In searches of databases and citation searches on ISI Web of Science we identified a number of published RCT protocols that are evaluating post-discharge interventions with a pain severity outcome at ≥12 months after TKR. Interventions being evaluated include a digital activity coaching system for home exercise [31], Wii-enhanced rehabilitation [32], group-based outpatient physiotherapy with an individualised element [33], multicomponent rehabilitation for patients at risk of a poor outcome [34] and physiotherapy for patients performing poorly at six weeks after TKR [35]. Some of these studies are now finished and findings are likely to be reported imminently.

DISCUSSION

This systematic review aimed to evaluate the effectiveness of post-discharge interventions delivered in the first three months after surgery for reducing the severity of chronic pain after TKR. Interventions that predominately comprise physiotherapy have been evaluated in RCTs. In most studies, the control group received some form of physiotherapy care and therefore the aim of the trials was to compare the effectiveness of different types of physiotherapy, rather than comparing the effectiveness of physiotherapy to no care. A narrative synthesis of the evidence suggests that no physiotherapy intervention appears to be more effective than another at reducing the severity of chronic pain after TKR. However, findings from the trial of a 6 month home-based functional exercise programme aimed at managing kinesiophobia [26] compared to advice to stay active were encouraging and warrant further evaluation. Few studies have been conducted to evaluate the effectiveness of non-physiotherapy interventions at reducing chronic pain after TKR, and further research is needed.

For peer review only - http://bmjopen.bmj.com/site/about/guidelines.xhtml

There are a number of strengths and limitations to this systematic review. The main outcome of interest in this review was pain severity at ≥12 months after TKR. Although the primary outcome of many of the included studies was function, pain severity was an important secondary outcome in these studies. Studies that used a composite pain and function measure to assess outcome, for example the OKS or WOMAC, were excluded if authors were unable to provide pain subscales scores. Although this reduced the number of studies eligible for inclusion, this approach was taken because pain and function are distinct outcome domains, with different predictors and recovery trajectories [36 37]. The secondary outcome of this review was adverse events, to allow the synthesis of harms data. However, synthesis was not possible because assessment and reporting of adverse events was variable and often poor. The quality of adverse events reporting is a common issue in surgical trials [38], and evidence-based recommendations are needed to promote standardisation, improve quality and reduce heterogeneity of adverse events reporting in orthopaedic studies. A potential limitation of the included studies was that they were all at high risk of bias due to the lack of participant blinding for self-report pain. However, blinding of participants is rarely possible in RCTs of this nature. Also, it would be expected that the risk would arise from participants in the intervention group reporting less pain, which may potentially be an issue with shorter-term outcomes, but this was not evident from the longer-term follow-up of the studies included in this review.

This systematic review took a broad approach by evaluating the effectiveness of any type of post-discharge intervention that aimed to reduce the severity of chronic pain after TKR. Interventions that span the post-operative period may be delivered as part of a comprehensive peri-operative package of care, and these would not have been identified in this review; however, evaluations of the effectiveness of pre-operative and peri-operative interventions for reducing chronic pain severity are being conducted separately (CRD42017041382).

For peer review only - http://bmjopen.bmj.com/site/about/guidelines.xhtml

Previous systematic reviews of interventions to improve long-term outcomes after TKR have been conducted, but these have evaluated pre-operative interventions or have been narrower in focus. Systematic reviews of pre-operative interventions have found that exercise and education have a limited effect on improving long-term pain and function after TKR [39-43]. Previous systematic reviews of post-discharge interventions have focussed on physiotherapy, finding some evidence of short-term benefit but a lack of evidence to draw conclusions about long-term benefit [6 44 45]. One systematic review has evaluated interventions for the management of chronic pain after TKR, identifying only a single RCT of botulinum toxin A injections [46]. Our systematic review adds to this literature by providing evidence that no specific type of post-discharge physiotherapy intervention appears to be more effective than another at reducing the severity of chronic pain after TKR, although the positive impact of a home-based programme aimed at managing kinesiophobia compared with advice to stay active warrants further investigation.

The aim of this review was to evaluate the effectiveness of post-discharge interventions at reducing chronic pain severity after TKR. However, the primary aim of most trials included in the review was to improve functional ability after TKR. Only one trial had a primary outcome of pain severity [29], although a number of other trials assessed their primary outcome with a composite measure of pain and function [17 20 24 26]. However, pain severity was assessed as a secondary outcome in these trials and therefore it was expected that the intervention may reduce long-term pain. All but one study found that the intervention did not provide any benefit on long-term pain severity compared to the control group. However, the treatment received in the control group, particularly in the physiotherapy trials, varied considerably between studies, including a different form or intensity of physiotherapy, provision of physiotherapy based on a needs assessment, delayed treatment or no treatment. Therefore, it is not appropriate to draw conclusions on the effectiveness of any particular type

For peer review only - http://bmjopen.bmj.com/site/about/guidelines.xhtml

of physiotherapy intervention based on the findings of this review. However, the evidence does suggest that no type of physiotherapy intervention is more effective than another at reducing the severity of chronic pain after TKR. An important finding of this review is that only four trials have been conducted which have evaluated non-physiotherapy interventions, highlighting the need for more research in this field. In particular, further research with pain severity as the pain outcome is needed to ensure that RCTs are adequately powered to evaluate the effectiveness of post-discharge interventions on reducing chronic pain severity.

There are important considerations in the design and delivery of future post-discharge interventions that warrant further discussion. All the interventions included in this review were uniformly delivered to all patients, rather than just those patients who may be at most risk of poor outcome. Only 20% of patients will develop chronic pain after TKR [2] and delivering physiotherapy to all patients may reduce the ability to detect clinical benefit in terms of pain severity. In the future, interventions might be more effective if they include processes to identify patients at high risk of chronic pain and provide these patients with intensive and comprehensive interventions that have been specifically designed to reduce their risk of developing chronic pain. However, identifying high risk patients is challenging; pre-operative models to identify patients at risk of a poor outcome have low predictive power [36 37], and the evidence for post-operative risk factors is limited [47]. However, research is currently ongoing to evaluate whether providing a rehabilitation programme for patients at risk of a poor outcome [48] or are ‘functioning poorly’ at six weeks after TKR [35] can improve longer-term outcomes.

Preventing chronic pain after TKR is challenging because of the complexity of this pain condition. Although chronic pain after TKR is not yet fully understood, the aetiology of this pain is multifactorial, including surgical factors, complex regional pain syndrome, pain sensitisation, neuropathic pain and psychosocial factors [49-54]. Therefore, an intervention

For peer review only - http://bmjopen.bmj.com/site/about/guidelines.xhtml

comprising a single treatment modality may be insufficient to address and reduce the causes of pain for all patients. As with the treatment of other chronic pain conditions, this highlights the importance of focused, individualised and multidisciplinary treatment [8 55]. Such an approach is being evaluated in an ongoing RCT of a care pathway for patients with chronic pain after TKR (ISRCTN92545361). Therefore, further research is needed to evaluate the effectiveness of providing stratified and multidisciplinary care packages for preventing chronic pain after TKR.

In conclusion, findings from this systematic review and narrative synthesis are that there is no evidence that one type of physiotherapy intervention is more effective than another at reducing the severity of chronic pain after TKR. Further research is needed to evaluate non-physiotherapy interventions, including the provision of care as part of stratified and multidisciplinary care package.

For peer review only

For peer review only - http://bmjopen.bmj.com/site/about/guidelines.xhtml

FIGURE LEGENDS

Figure 1: Systematic review flow diagram

Figure 2: Risk of bias assessment for individual studies

For peer review only

For peer review only - http://bmjopen.bmj.com/site/about/guidelines.xhtml

ACKNOWLEDGEMENTS

We would like to thank all the study authors who took the time to reply to our requests for further clarification or additional data.

AUTHOR CONTRIBUTIONS

All authors contributed to the concept and design of the study. ADB, JD and VW contributed to the acquisition and analysis of data. VW drafted the article and ADB, JD and RGH revised it critically for important intellectual content. VW and ADB take responsibility for the integrity of the work as a whole, from inception to finished article.

FUNDING

This article presents independent research funded by the National Institute for Health Research (NIHR) under its Programme Grants for Applied Research programme (RP-PG-0613-20001). The views expressed are those of the authors and not necessarily those of the NHS, the NIHR or the Department of Health. The funder had no involvement in the study design, data collection, data analysis, interpretation of data or writing of the manuscript.

This study was supported by the NIHR Biomedical Research Centre at the University Hospitals Bristol NHS Foundation Trust and the University of Bristol. The views expressed in this publication are those of the author(s) and not necessarily those of the NHS, the National Institute for Health Research or the Department of Health.

COMPETING INTERESTS

The authors have no conflicts of interest to declare.

DATA SHARING STATEMENT

No additional data are available.

For peer review only - http://bmjopen.bmj.com/site/about/guidelines.xhtml

Table 1: Overview of study characteristics

Publication<br>Location<br>Date of study<br>Number of centres	Randomised<br>Mean age<br>% female	Intervention treatment	Control treatment	Pain assessment<br>Adherence to treatment<br>Losses to follow-up
Bruun-Olsen et al, 2013<br>Norway<br>2008-2010<br>2 centres	N=57(29:28)<br>68:69 years<br>62:50%	Group-based physiotherapist-led walking skills programme (2-6 patients per group). Commenced 6 weeks after surgery. 12 sessions over 6-8 weeks.	1:1 usual physiotherapy care consisting of 12 individual physiotherapy sessions. Commenced 6 weeks after surgery. Twice weekly sessions until 12-14 weeks after surgery.	KOOS pain scale<br>28/29 completed intervention<br>28/28 received control treatment<br>6 (2:4) lost to follow-up
Buhagiar et al, 2017<br>Australia<br>2012-2015<br>2 centres	N=165(81:84)<br>67:67 years<br>69:68%	Inpatient rehabilitation at rehabilitation facility with twice daily supervised sessions of 1:1 and group-based exercises. Commenced after hospital discharge for 10 days. Home exercise programme after discharge from rehabilitation facility.	Home exercise programme comprising of 2-3 group-based outpatient session to practice and progress exercises. Commenced 2 weeks after surgery.	KOOS pain scale<br>72/81 adhered to intervention<br>74/84 adhered to control treatment<br>6(2:4) lost to follow-up
Buker et al, 2014*<br>Turkey<br>2009-2011<br>1 centre	N=34 (18:16)<br>64:68 years<br>89:94%	20 sessions of supervised physiotherapy and rehabilitation including range of motion and strengthening exercises, application of heat and TENS application. Five days a week for four weeks.	Home exercises including range of motion and strengthening exercise for one hour per day. Five days a week for four weeks.	Pain VAS<br>Adherence not reported<br>Losses to follow-up not reported
Chen et al, 2016<br>China<br>2013-2014<br>1 centre	N=202(101:101)<br>66:67 years<br>63:67%	Structured telephone follow-up by nurse at 1, 3 and 6 weeks after hospital discharge to improve adherence to home exercise routine.	No telephone follow-up	Pain VAS<br>Adherence not reported<br>15(7:8) lost to follow-up
Fransen et al, 2017<br>Australia<br>2009-2012	N=422(212:210)<br>64:65 years<br>54:52%	Group-based circuit exercise classes supervised by physiotherapist. Up to 6 patients per class. Commenced 6 weeks after surgery. Twice	Usual physiotherapy care. 22% of participants reported 6 or more occasions of physiotherapy	WOMAC pain scale<br>140/212 participants attended ≥12 classes

For peer review only - http://bmjopen.bmj.com/site/about/guidelines.xhtml

12 centres		weekly sessions for at least 8 weeks.	during the 6–12-week period after TKR	210/210 received control treatment<br>74(43:31) lost to follow-up
Frost et al, 2002<br>UK<br>1995-1996<br>Not reported	N=47(23:24)<br>72:71 years<br>48:50%	Home-based functional exercise. Commenced following discharge from hospital. Duration not reported.	Home-based traditional exercise.	OKS item (pain on walking)<br>Adherence not reported<br>20 (7:13) lost to follow-up
Kauppila et al, 2010[$]<br>Finland<br>2002-2005<br>1 centre	N=86(44:42)<br>71:71 years<br>76:79%	Group-based multidisciplinary rehabilitation programme. Up to 8 patients per group. Commenced 2-4 months after surgery for 10 days.	Usual physiotherapy care. Supervised exercise programme at 2 month outpatient visit, with provision of further rehabilitation based on needs assessment.	WOMAC pain scale<br>44/44 attended intervention<br>42:42 received control treatment<br>11 (8:3) lost to follow-up
Ko et al, 2013<br>Australia<br>2008-2010<br>3 arm trial<br>4 centres	N= 249(85:84:80)<br>67:68:67 years<br>68:60:61%	1:1 physiotherapy with home-based sessions. Commenced 2 weeks after surgery. Twice weekly 1:1 and home-based sessions over 6 weeks.	1.) Group-based circuit classes supervised by physiotherapist with home-based sessions. Up to 8 patients per class. Commenced 2 weeks after surgery. Twice weekly group and home-based sessions over 6 weeks.<br><br>2.) Monitored home programme, 2 1:1 physiotherapy sessions and 1 telephone follow-up call. Commenced 2 weeks after surgery. 4 sessions per week for 6 weeks.	WOMAC pain scale<br>80% participants attended 9 or more 1:1 sessions, 77% attended 9 or more group sessions, 83% attended both sessions in monitored home programme group.<br>16(7:3:6) lost to follow-up
Kramer et al, 2003<br>Canada<br>Not reported<br>Not reported	N=160(80:80)<br>68:69 years<br>59:55%	1:1 clinic-based rehabilitation programme with home exercise programme. Commenced 2 weeks after surgery. Up to 2 sessions a week for 10 weeks.	Home-based rehabilitation, monitored by telephone calls from physiotherapist. Commenced 2 weeks after surgery. At least 1 telephone call in weeks 2-6 and 1 call in	WOMAC pain scale<br>76/80 received intervention<br>78/80 received control treatment<br>26 (15:22) lost to follow-up

For peer review only

For peer review only - http://bmjopen.bmj.com/site/about/guidelines.xhtml

			weeks 7-12.	
Liebs et al, 2010*<br>Germany<br>2005-2006<br>5 centres	N=159(85:74)<br>70:70 years<br>73:70%	Ergometer cycling supervised by physiotherapist. Commenced 2 weeks after surgery. 3 sessions a week for at least 3 weeks.	No ergometer cycling.	WOMAC pain scale<br>Adherence not reported<br>33(15:18) lost to follow-up
Liebs et al, 2012*<br>Germany<br>2003-2004<br>2 centres	N=185(87:98)<br>69:71 years<br>70:73%	Early aquatic therapy. Commenced 6 days after surgery. 3 times a week up to 5th week post-operative.	Late aquatic therapy. Commenced 14 days after surgery. 3 times a week up to 5th week post-operative.	WOMAC pain scale<br>Adherence not reported<br>41(18:23) lost to follow-up
Minns Lowe et al, 2012<br>UK<br>2006-2008<br>1 centre	N=107 (56:51)<br>68:71 years<br>57:59%	Home-based functional rehabilitation with 2 visits from physiotherapist at 2 weeks and 6-8 weeks after hospital discharge. Twice daily exercises for at least 3 months.	Usual physiotherapy care involving provision of an exercise booklet, with outpatient physiotherapy on a needs only basis. No additional home visits.	KOOS pain scale<br>46/56 patients received 2 visits<br>47/51 received control treatment<br>9 (7:2) lost to follow-up
Moffet et al, 2004 [25]<br>Canada<br>1997-1999<br>5 centres	N=77(38:39)<br>67:69 years<br>63:56%	Functional rehabilitation programme with individualised home exercises. Commenced at 2 months after surgery. 12 supervised sessions over 6-8 weeks.	Usual physiotherapy care, which included supervised home rehabilitation visits for 26% of patients.	WOMAC pain scale<br>38/38 participated in 12 sessions<br>39/39 received control treatment<br>8(0:8) lost to follow-up
Monticone et al, 2010-2013<br>Italy<br>1 centre	N=110(55:55)<br>67:68 years<br>65:62%	Home-based functional exercises aimed at managing kinesiophobia, with monthly phone calls to encourage adherence. Commenced after hospital discharge. Twice weekly sessions for 6 months.	No physiotherapy, advice to stay active.	KOOS pain scale<br>Adherence not reported<br>0 lost to follow-up
Petterson et al, 2009<br>USA<br>200-2005<br>1 centre	N=200(100:100)<br>65:65 years<br>47:45%	Combined neuromuscular electric stimulation (NMES) and volitional strength training programme. Commenced 3-4 weeks after surgery. 2 or 3 sessions a week for 6 weeks.	Volitional strength training program without NMES.	KOS ADL item (effect of pain on function)<br>84/100 completed intervention<br>97/100 completed control treatment<br>51 (32:19) lost to follow-up
Szots et al, 2016<br>Denmark	N=117(59:58)<br>67:68 years	Two nurse-led structured telephone follow-up calls. Telephone calls at 4 days and 14 days	No telephone follow-up.	WOMAC pain scale<br>54/59 patients had both telephone

For peer review only - http://bmjopen.bmj.com/site/about/guidelines.xhtml

2013<br>1 centre	61:67%	after hospital discharge.		follow-up calls<br>54/58 received control treatment<br>9(5:4) lost to follow-up
Vuorenmaa et al, 2014 **<br>Finland<br>2008-2010<br>1 centre	N=108(53:55)<br>69:69 years<br>57:65%	Delayed monitored home exercises, with guidance from physiotherapist at 2, 3 and 6 months post-operative. Commenced at 2 months after surgery for 12 months.	Usual care, which involved no additional guidance from 2 months post-operative.	WOMAC pain scale<br>72% of patients performed the training sessions at least twice per week in the first 6 months<br>53/53 received control treatment<br>4(2:2) lost to follow-up

* 24 month follow-up also conducted but data from 12 month follow-up included in table to be consistent with follow-up period of other studies

** Follow-up at 14 months post-operative

$ Pain-specific outcome data was provided by the authors for a previous review [56] and was used again in this review

For peer review only - http://bmjopen.bmj.com/site/about/guidelines.xhtml

REFERENCES

1. Werner MU, Kongsgaard UE. I. Defining persistent post-surgical pain: is an update required? Br J Anaesth 2014;**113**(1):1-4.

2. Beswick AD, Wylde V, Gooberman-Hill R, et al. What proportion of patients report long-term pain after total hip or knee replacement for osteoarthritis? A systematic review of prospective studies in unselected patients. BMJ Open 2012;**2**(1):e000435.

3. Scott CE, Howie CR, MacDonald D, et al. Predicting dissatisfaction following total knee replacement: a prospective study of 1217 patients. J Bone Joint Surg Br 2010;**92**(9):1253-8.

4. Jeffery AE, Wylde V, Blom AW, et al. "It's there and I'm stuck with it": patients' experiences of chronic pain following total knee replacement surgery. Arthritis Care Res (Hoboken) 2011;**63**(2):286-92.

5. Artz N, Dixon S, Wylde V, et al. Physiotherapy provision following discharge after total hip and total knee replacement: a survey of current practice at high-volume NHS hospitals in England and wales. Musculoskeletal Care 2013;**11**(1):31-8.

6. Artz N, Elvers K, Minns Lowe C, et al. Effectiveness of physiotherapy exercise following total knee replacement: systematic review and meta-analysis. BMC Musculoskeletal Disorders 2015;**16**(15).

7. Khan F, Ng L, Gonzalez S, et al. Multidisciplinary rehabilitation programmes following joint replacement at the hip and knee in chronic arthropathy. Cochrane Database Syst Rev 2008(2):CD004957.

8. Turk DC, Wilson HD, Cahana A. Treatment of chronic non-cancer pain. Lancet 2011;**377**(9784):2226-35.

9. Higgins J, Green SE. Cochrane Handbook for Systematic Reviews of Interventions Version 5.1.0 [updated March 2011]. The Cochrane Collaboration, 2011. Available from www.cochrane-handbook.org, 2011.

10. Moher D, Liberati A, Tetzlaff J, et al. Preferred reporting items for systematic reviews and meta-analyses: the PRISMA statement. BMJ 2009;**339**:b2535.

11. Lenguerrand E, Wylde V, Gooberman-Hill R, et al. Trajectories of Pain and Function after Primary Hip and Knee Arthroplasty: The ADAPT Cohort Study. PLoS One 2016;**11**(2):e0149306.

For peer review only - http://bmjopen.bmj.com/site/about/guidelines.xhtml

12. Kennedy DM, Stratford PW, Hanna SE, et al. Modeling early recovery of physical function following hip and knee arthroplasty. BMC Musculoskelet Disord 2006;**7**:100.

13. Review Manager. (RevMan) [Computer program]. Version 5.3. Copenhagen: The Nordic Cochrane Centre, The Cochrane Collaboration. 2014.

14. Bruun-Olsen V, Heiberg KE, Wahl AK, et al. The immediate and long-term effects of a walking-skill program compared to usual physiotherapy care in patients who have undergone total knee arthroplasty (TKA): a randomized controlled trial. Disability and rehabilitation 2013;**35**(23):2008-15.

15. Buhagiar MA, Naylor JM, Harris IA, et al. Effect of Inpatient Rehabilitation vs a Monitored Home-Based Program on Mobility in Patients With Total Knee Arthroplasty: The HIHO Randomized Clinical Trial. JAMA 2017;**317**(10):1037-46.

16. Chen M, Li P, Lin F. Influence of structured telephone follow-up on patient compliance with rehabilitation after total knee arthroplasty. Patient Preference and Adherence 2016;**10**:257-64.

17. Fransen M, Nairn L, Bridgett L, et al. Post-Acute Rehabilitation After Total Knee Replacement: A Multicenter Randomized Clinical Trial Comparing Long-Term Outcomes. Arthritis Care Res (Hoboken) 2017;**69**(2):192-200.

18. Frost H, Lamb SE, Robertson S. A randomized controlled trial of exercise to improve mobility and function after elective knee arthroplasty. Feasibility, results and methodological difficulties. Clinical rehabilitation 2002;**16**(2):200-9.

19. Kauppila AM, Kyllonen E, Ohtonen P, et al. Multidisciplinary rehabilitation after primary total knee arthroplasty: a randomized controlled study of its effects on functional capacity and quality of life. Clinical rehabilitation 2010;**24**(5):398-411.

20. Ko V, Naylor J, Harris I, et al. One-to-one therapy is not superior to group or home-based therapy after total knee arthroplasty: a randomized, superiority trial. The Journal of bone and joint surgery. American volume 2013;**95**(21):1942-9.

21. Kramer JF, Speechley M, Bourne R, et al. Comparison of clinic- and home-based rehabilitation programs after total knee arthroplasty. Clinical orthopaedics and related research 2003(410):225-34.

22. Liebs TR, Herzberg W, Ruther W, et al. Ergometer cycling after hip or knee replacement surgery: a randomized controlled trial. The Journal of bone and joint surgery. American volume 2010;**92**(4):814-22.

For peer review only - http://bmjopen.bmj.com/site/about/guidelines.xhtml

23. Liebs T, Herzberg W, Rüther W, et al. Multicenter randomized controlled trial comparing early versus late aquatic therapy after total hip or knee arthroplasty. Archives of physical medicine and rehabilitation 2012; 93(2). http://onlinelibrary.wiley.com/o/cochrane/clcentral/articles/334/CN-00832334/frame.html

http://ac.els-cdn.com/S0003999311008434/1-s2.0-S0003999311008434-main.pdf?_tid=41aa00ce-f508-11e6-b06b-00000aab0f27&acdnat=1487332829_31eb2562ac20096dc09cc02ed9cf1f7e.

24. Minns Lowe CJ, Barker KL, Holder R, et al. Comparison of postdischarge physiotherapy versus usual care following primary total knee arthroplasty for osteoarthritis: an exploratory pilot randomized clinical trial. Clinical rehabilitation 2012;**26**(7):629-41.

25. Moffet H, Collet JP, Shapiro SH, et al. Effectiveness of intensive rehabilitation on functional ability and quality of life after first total knee arthroplasty: A single-blind randomized controlled trial. Archives of physical medicine and rehabilitation 2004;**85**(4):546-56.

26. Monticone M, Ferrante S, Rocca B, et al. Home-based functional exercises aimed at managing kinesiophobia contribute to improving disability and quality of life of patients undergoing total knee arthroplasty: a randomized controlled trial. Archives of physical medicine and rehabilitation 2013;**94**(2):231-9.

27. Petterson SC, Mizner RL, Stevens JE, et al. Improved function from progressive strengthening interventions after total knee arthroplasty: a randomized clinical trial with an imbedded prospective cohort. Arthritis and rheumatism 2009;**61**(2):174-83.

28. Szöts K, Konradsen H, Solgaard S, et al. Long-term effects of telephone follow-up after total knee arthroplasty. Journal of Nursing Education and Practice 2016;**6**(7):151-54.

29. Vuorenmaa M, Ylinen J, Piitulainen K, et al. Efficacy of a 12-month, monitored home exercise programme compared with normal care commencing 2 months after total knee arthroplasty: a randomized controlled trial. Journal of rehabilitation medicine 2014;**46**(2):166-72.

30. Buker N, Akkaya S, Akkaya N, et al. Comparison of effects of supervised physiotherapy and a standardized home program on functional status in patients with total knee arthroplasty: a prospective study. J Phys Ther Sci 2014;**26**(10):1531-6.

31. Harmelink KEM, Zeegers A, Tonis TM, et al. The effectiveness of the use of a digital activity coaching system in addition to a two-week home-based exercise program in

For peer review only

For peer review only - http://bmjopen.bmj.com/site/about/guidelines.xhtml

patients after total knee arthroplasty: study protocol for a randomized controlled trial. BMC Musculoskelet Disord 2017;**18**(1):290.

32. Negus JJ, Cawthorne DP, Chen JS, et al. Patient outcomes using Wii-enhanced rehabilitation after total knee replacement - the TKR-POWER study. Contemp Clin Trials 2015;**40**:47-53.
33. Wylde V, Artz N, Marques E, et al. Effectiveness and cost-effectiveness of outpatient physiotherapy after knee replacement for osteoarthritis: study protocol for a randomised controlled trial. Trials 2016;**17**(1):289.
34. Barker KL, Beard D, Price A, et al. COmmunity-based Rehabilitation after Knee Arthroplasty (CORKA): Study protocol for a randomised controlled trial. Trials 2016;**17**(1):no pagination.
35. Simpson AH, Hamilton DF, Beard DJ, et al. Targeted rehabilitation to improve outcome after total knee replacement (TRIO): study protocol for a randomised controlled trial. Trials 2014;**15**:44.
36. Judge A, Arden NK, Cooper C, et al. Predictors of outcomes of total knee replacement surgery. Rheumatology (Oxford) 2012;**51**(10):1804-13.
37. Lopez-Olivo MA, Landon GC, Siff SJ, et al. Psychosocial determinants of outcomes in knee replacement. Ann Rheum Dis 2011;**70**(10):1775-81.
38. Wylde V, Dennis J, Beswick AD, et al. Systematic review of management of chronic pain after surgery. British Journal of Surgery 2017;**104**(10):1293-306.
39. Blom AW, Artz N, Beswick AD, et al. Improving patients' experience and outcome of total joint replacement: the RESTORE programme. NIHR Journals Library. Southampton (UK), 2016.
40. Peer MA, Rush R, Gallacher PD, et al. Pre-surgery exercise and post-operative physical function of people undergoing knee replacement surgery: A systematic review and meta-analysis of randomized controlled trials. J Rehabil Med 2017;**49**(4):304-15.
41. Jordan RW, Smith NA, Chahal GS, et al. Enhanced education and physiotherapy before knee replacement; is it worth it? A systematic review. Physiotherapy 2014;**100**(4):305-12.
42. Wang L, Lee M, Zhang Z, et al. Does preoperative rehabilitation for patients planning to undergo joint replacement surgery improve outcomes? A systematic review and meta-analysis of randomised controlled trials. BMJ Open 2016;**6**(2):e009857.

For peer review only

For peer review only - http://bmjopen.bmj.com/site/about/guidelines.xhtml

43. Chesham RA, Shanmugam S. Does preoperative physiotherapy improve postoperative, patient-based outcomes in older adults who have undergone total knee arthroplasty? A systematic review. Physiother Theory Pract 2017;**33**(1):9-30.

44. Minns Lowe CJ, Barker KL, Dewey M, et al. Effectiveness of physiotherapy exercise after knee arthroplasty for osteoarthritis: systematic review and meta-analysis of randomised controlled trials. BMJ 2007;**335**(7624):812.

45. Florez-Garcia M, Garcia-Perez F, Curbelo R, et al. Efficacy and safety of home-based exercises versus individualized supervised outpatient physical therapy programs after total knee arthroplasty: a systematic review and meta-analysis. Knee Surg Sports Traumatol Arthrosc 2017;**25**(11):3340-53.

46. Beswick AD, Wylde V, Gooberman-Hill R. Interventions for the prediction and management of chronic postsurgical pain after total knee replacement: systematic review of randomised controlled trials. BMJ Open 2015;**5**(5):e007387.

47. Wylde V, Beswick A, Dennis J, et al. Post-operative patient-related risk factors for chronic pain after total knee replacement: a systematic review. BMJ Open In press.

48. Barker KL, Beard D, Price A, et al. COmmunity-based Rehabilitation after Knee Arthroplasty (CORKA): study protocol for a randomised controlled trial. Trials 2016;**17**(1):501.

49. Haroutiunian S, Nikolajsen L, Finnerup NB, et al. The neuropathic component in persistent postsurgical pain: A systematic literature review. Pain 2013;**154**(1):95-102.

50. Howells N, Murray J, Wylde V, et al. Persistent pain after knee replacement: do factors associated with pain vary with degree of patient dissatisfaction? Osteoarthritis Cartilage 2016;**24**(12):2061-68.

51. Mandalia V, Eyres K, Schranz P, et al. Evaluation of patients with a painful total knee replacement. J Bone Joint Surg Br 2008;**90**(3):265-71.

52. Toms AD, Mandalia V, Haigh R, et al. The management of patients with painful total knee replacement. J Bone Joint Surg Br 2009;**91**(2):143-50.

53. van Bussel CM, Stronks DL, Huygen FJ. Complex regional pain syndrome type I of the knee: a systematic literature review. Eur J Pain 2014;**18**(6):766-73.

54. Vissers MM, Bussmann JB, Verhaar JA, et al. Psychological factors affecting the outcome of total hip and knee arthroplasty: a systematic review. Semin Arthritis Rheum 2012;**41**(4):576-88.

For peer review only - http://bmjopen.bmj.com/site/about/guidelines.xhtml

55. Lavand'homme P, Thienpont E. Pain after total knee arthroplasty: a narrative review focusing on the stratification of patients at risk for persistent pain. Bone Joint J 2015;**97-B**(10 Suppl A):45-8.

56. Artz N, Elvers KT, Lowe CM, et al. Effectiveness of physiotherapy exercise following total knee replacement: systematic review and meta-analysis. BMC musculoskeletal disorders 2015;**16**:15.

For peer review only

For peer review only - http://bmjopen.bmj.com/site/about/guidelines.xhtml

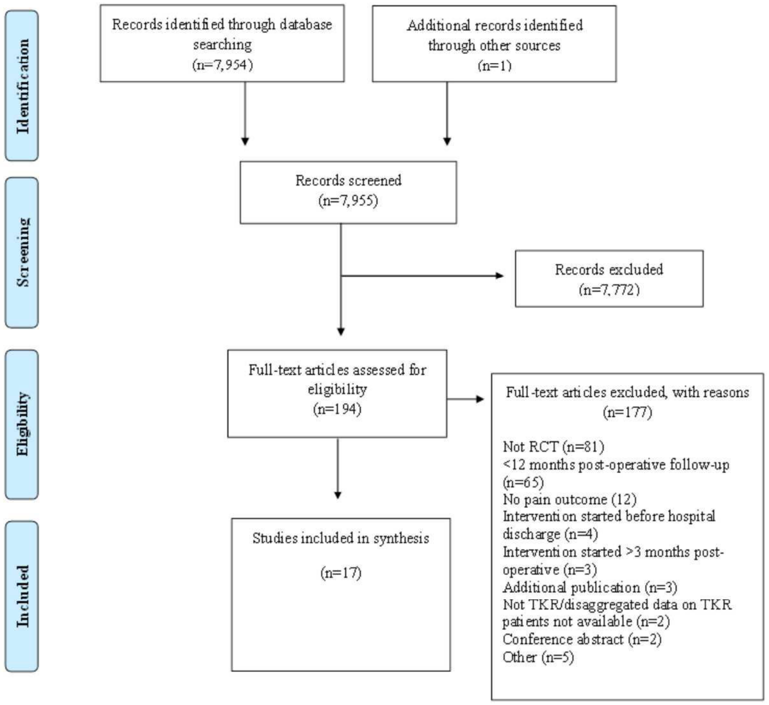

Figure 1: Systematic review flow diagram

254x190mm (300 x 300 DPI)

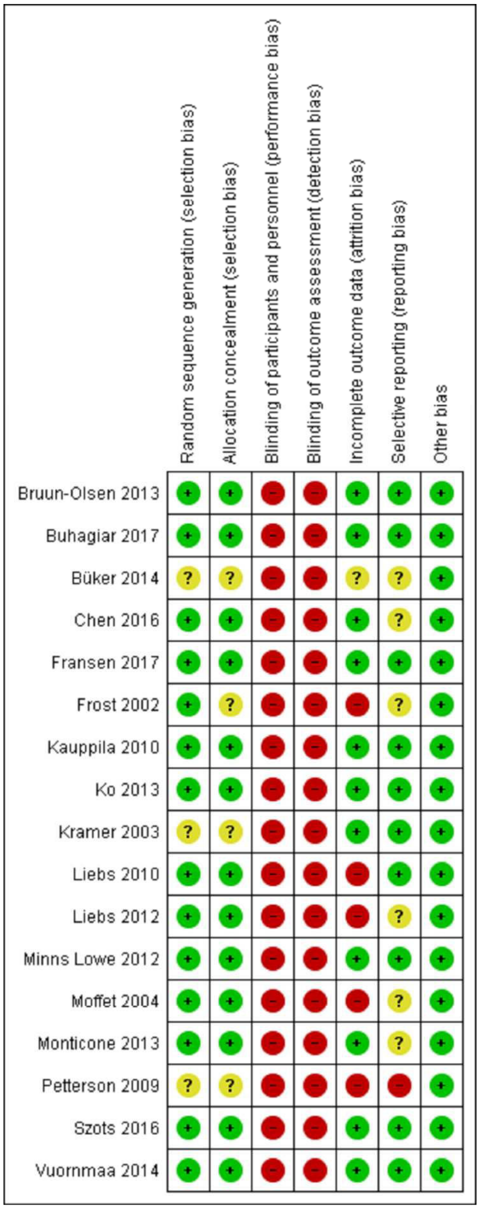

Figure 2: Risk of bias assessment for individual studies

211x535mm (300 x 300 DPI)

Appendix 1: PRISMA checklist

Section/topic	#	Checklist item	Reported on page #
**TITLE**			
Title	1	Identify the report as a systematic review, meta-analysis, or both.	1
**ABSTRACT**			
Structured summary	2	Provide a structured summary including, as applicable: background; objectives; data sources; study eligibility criteria, participants, and interventions; study appraisal and synthesis methods; results; limitations; conclusions and implications of key findings; systematic review registration number.	2-3
**INTRODUCTION**			
Rationale	3	Describe the rationale for the review in the context of what is already known.	4
Objectives	4	Provide an explicit statement of questions being addressed with reference to participants, interventions, comparisons, outcomes, and study design (PICOS).	4-5
**METHODS**			
Protocol and registration	5	Indicate if a review protocol exists, if and where it can be accessed (e.g., Web address), and, if available, provide registration information including registration number.	5
Eligibility criteria	6	Specify study characteristics (e.g., PICOS, length of follow-up) and report characteristics (e.g., years considered, language, publication status) used as criteria for eligibility, giving rationale.	5-6
Information sources	7	Describe all information sources (e.g., databases with dates of coverage, contact with study authors to identify additional studies) in the search and date last searched.	6
Search	8	Present full electronic search strategy for at least one database, including any limits used, such that it could be repeated.	Appendix 2
Study selection	9	State the process for selecting studies (i.e., screening, eligibility, included in systematic review, and, if applicable, included in the meta-analysis).	6-7
Data collection process	10	Describe method of data extraction from reports (e.g., piloted forms, independently, in duplicate) and any processes for obtaining and confirming data from investigators.	6-7
Data items	11	List and define all variables for which data were sought (e.g., PICOS, funding sources) and any assumptions and simplifications made.	6-7
Risk of bias in individual studies	12	Describe methods used for assessing risk of bias of individual studies (including specification of whether this was done at the study or outcome level), and how this information is to be used in any data synthesis.	7
Summary measures	13	State the principal summary measures (e.g., risk ratio, difference in means).	7
Synthesis of results	14	Describe the methods of handling data and combining results of studies, if done, including measures of consistency (e.g., I^2) for each meta-analysis.	7

For peer review only

Appendix 1: PRISMA checklist

Section/topic	#	Checklist item	Reported on page #
Risk of bias across studies	15	Specify any assessment of risk of bias that may affect the cumulative evidence (e.g., publication bias, selective reporting within studies).	7
Additional analyses	16	Describe methods of additional analyses (e.g., sensitivity or subgroup analyses, meta-regression), if done, indicating which were pre-specified.	7
**RESULTS**			
Study selection	17	Give numbers of studies screened, assessed for eligibility, and included in the review, with reasons for exclusions at each stage, ideally with a flow diagram.	Figure 1
Study characteristics	18	For each study, present characteristics for which data were extracted (e.g., study size, PICOS, follow-up period) and provide the citations.	Table 1
Risk of bias within studies	19	Present data on risk of bias of each study and, if available, any outcome level assessment (see item 12).	Table 2
Results of individual studies	20	For all outcomes considered (benefits or harms), present, for each study: (a) simple summary data for each intervention group (b) effect estimates and confidence intervals, ideally with a forest plot.	Figure 3, Appendix 3 and 4
Synthesis of results	21	Present results of each meta-analysis done, including confidence intervals and measures of consistency.	N/A
Risk of bias across studies	22	Present results of any assessment of risk of bias across studies (see Item 15).	Table 2
Additional analysis	23	Give results of additional analyses, if done (e.g., sensitivity or subgroup analyses, meta-regression [see Item 16]).	N/A
**DISCUSSION**			
Summary of evidence	24	Summarize the main findings including the strength of evidence for each main outcome; consider their relevance to key groups (e.g., healthcare providers, users, and policy makers).	12-15
Limitations	25	Discuss limitations at study and outcome level (e.g., risk of bias), and at review-level (e.g., incomplete retrieval of identified research, reporting bias).	12-13
Conclusions	26	Provide a general interpretation of the results in the context of other evidence, and implications for future research.	13-15
**FUNDING**			
Funding	27	Describe sources of funding for the systematic review and other support (e.g., supply of data); role of funders for the systematic review.	16

For peer review only

Appendix 2: Search terms

MEDLINE (Ovid) (1946 to 15 November 2016)

1 randomized controlled trial/ or randomized controlled trial.pt.

2 controlled clinical trial.pt.

3 randomized.ab.

4 placebo.ab.

5 randomly.ab

6 trial.ab

7 randomised.tw

8 1 or 2 or 3 or 4 or 5 or 6 or 7

9 review/

10 'systematic review$'.mp

11 9 or 10

12 8 or 11

13 Arthroplasty, Replacement, Knee/

14 Knee Prosthesis/

15 (arthoplast$ adj3 knee$).mp. [mp=title, abstract, original title, name of substance word, subject heading word, keyword heading word, protocol supplementary concept word, rare disease supplementary concept word, unique identifier]

16 (knee$ adj3 replac$).mp. [mp=title, abstract, original title, name of substance word, subject heading word, keyword heading word, protocol supplementary concept word, rare disease supplementary concept word, unique identifier]

17 (knee adj3 implant$).mp. [mp=title, abstract, original title, name of substance word, subject heading word, keyword heading word, protocol supplementary concept word, rare disease supplementary concept word, unique identifier]

18 13 or 14 or 15 or 16 or 17

19 12 and 18

EMBASE (Ovid) (1980 to 15 November 2016)

1 Randomized controlled trial/ or Randomization/ or Single blind procedure/ or Double blind procedure/ or Crossover procedure/ or Placebo/ or Randomised controlled trial$.tw. or Randomized controlled trial$.tw. or RCT.tw. or Random allocation.tw. or Randomly allocated.tw. or Allocated randomly.tw. or (allocated adj2 random).tw. or Single blind$.tw. or Double blind$.tw. or ((treble or triple) adj blind$).tw. or Placebo$.tw.

2 "systematic review"/

3 meta analysis/

For peer review only - http://bmjopen.bmj.com/site/about/guidelines.xhtml

4 2 or 3

5 1 or 4

6 knee arthroplasty/

7 total knee arthroplasty/

8 knee prosthesis/

9 (knee$ adj3 arthoplast$).mp. [mp=title, abstract, heading word, drug trade name, original title, device manufacturer, drug manufacturer, device trade name, keyword, floating subheading]

10 (knee$ adj3 replac$).mp. [mp=title, abstract, heading word, drug trade name, original title, device manufacturer, drug manufacturer, device trade name, keyword, floating subheading]

11 (knee$ adj3 implant$).mp. [mp=title, abstract, heading word, drug trade name, original title, device manufacturer, drug manufacturer, device trade name, keyword, floating subheading]

12 6 or 7 or 8 or 9 or 10 or 11

13 5 and 12

PsycINFO (Ovid) (inception [1806] to 15 November 2016)

1. (knee$ adj3 arthoplast$).mp. [mp=title, abstract, heading word, table of contents, key concepts, original title, tests & measures]

2. (knee$ adj3 replac$).mp. [mp=title, abstract, heading word, table of contents, key concepts, original title, tests & measures]

3. (knee$ adj3 surg$).mp. [mp=title, abstract, heading word, table of contents, key concepts, original title, tests & measures]

4. (knee$ adj3 implant$).mp. [mp=title, abstract, heading word, table of contents, key concepts, original title, tests & measures]

5. (knee adj3 prosthe$).mp. [mp=title, abstract, heading word, table of contents, key concepts, original title, tests & measures]

6. 1 or 2 or 3 or 4 or 5

The Cochrane Library (Wiley) (inception to 15 November 2016)

#1 MeSH descriptor: [Knee Prosthesis] explode all trees

#2 MeSH descriptor: [Arthroplasty, Replacement, Knee] explode all trees

#3 arthoplast* N3 knee*

#4 knee* N3 replac*

#5 knee* N3 implant*

#6 #1 or #2 or #3 or #4 or #5

For peer review only - http://bmjopen.bmj.com/site/about/guidelines.xhtml

CINAHL (EBSCOHOST) (1982 to 15 November 2016)

S25	S15 AND S23 Limiters - Exclude MEDLINE records
S24	S15 AND S23
S23	S16 OR S17 OR S18 OR S19 OR S20 OR S21 OR S22
S22	knee* N3 implant*
S21	knee* N3 arthoplast*
S20	arthoplast* N3 knee*
S19	knee* N3 replac*
S18	"knee prosthes*"
S17	MH "Knee surgery"
S16	MH "Arthroplasty, Replacement, Knee"
S15	(S8 OR S14)
S14	(S9 OR S10 OR S11 OR S12 OR S13)
S13	metaanalyses
S12	metaanalysis
S11	meta-analyses
S10	meta-analysis
S9	systematic review
S8	S1 OR S2 OR S3 OR S4 OR S5 OR S6 OR S7
S7	AB trial$
S6	AB randomly
S5	AB randomised OR randomized
S4	(MH "clinical trials")
S3	clinical trials
S2	(MH "randomized controlled trials")
S1	randomized controlled trials

For peer review only - http://bmjopen.bmj.com/site/about/guidelines.xhtml

Appendix 3: Trial results summary

Study	Primary aim of the study	Primary outcome (and measure)	Results summary
Bruun-Olsen et al, 2013	To examine the immediate and long-term effects of a walking-skill program compared with usual physiotherapy care.	Walking distance (6 minute walk test)	No significant difference in mean 0-100 KOOS pain scores (p-value not reported):<br>Intervention: 82 (SD 21)<br>Control: 74 (SD 23)<br><br>Mean change from baseline (6 weeks post-operative):<br>Intervention: 21<br>Control: 20
Buhagiar et al, 2017	To determine if 10 days of inpatient rehabilitation followed by a monitored home-based program provided greater improvements than a monitored home-based program alone.	Walking distance (6 minute walk test)	No significant difference in median 0-100 KOOS pain scores between groups (p-value not reported):<br>Intervention: 86 (IQR 74 to 97)<br>Control: 91 (IQR 78-98)<br><br>Mean change from baseline (pre-surgery):<br>Intervention: 53<br>Control: 55
Buker et al, 2014	Determine the functional differences between patients who were treated with supervised physiotherapy or a standardized home program and perform a cost analysis.	Not specified	No significant difference in mean pain 0-10 VAS scores (p-value not reported):<br>Rest pain - Intervention: 0.44 (SD 0.51); Control: 0.37 (SD 0.80)<br>Activity pain - Intervention: 3.11 (SD 1.96); Control: 2.50 (SD 1.77)<br><br>Mean change from baseline (pre-surgery):<br>Rest pain - Intervention: 4.86; Control: 4.78<br>Activity pain - Intervention: 6.14; Control: 5.46
Chen et al, 2016	To assess the impact of structured telephone reinforcement on patient	Not specified (pilot study)	No significant difference in mean 0-100 VAS pain scores (p-value not reported):

For peer review only

Appendix 3: Trial results summary

Study	Primary aim of the study	Primary outcome (and measure)	Results summary
	compliance with home exercises.		Intervention: 8.7 (SD 5.1)<br>Control: 9.3 (SD 5.5)<br><br>Mean change from baseline (pre-surgery):<br>Intervention: 63.5<br>Control: 63.8
Fransen et al, 2017	To evaluate the long-term benefit of providing a post-acute, class-based outpatient exercise program compared with current usual rehabilitation care.	Pain and function (WOMAC pain and function scales)	No significant difference in mean 0-20 WOMAC pain scores (p=0.71):<br>Intervention: 2.6 (SE 0.2)<br>Control: 2.5 (SE 0.2)<br><br>Mean change from baseline (pre-surgery):<br>Intervention: 8.7<br>Control: 8.5
Frost et al, 2002	To assess the feasibility of comparing traditional exercise regimes with a more functional and dynamic approach.	Not specified (feasibility study)	No significant difference in mean 1-5 OKS item 'pain on walking' scores (p=0.68):<br>Intervention: 1.6 (SD 0.8)<br>Control: 1.5 (SD 0.93)<br><br>Mean change from baseline (pre-surgery):<br>Intervention: 2.6<br>Control: 2.8
Kauppila et al, 2010	To examine whether a multidisciplinary rehabilitation programme can improve functional recovery and quality of life and reduce the use of rehabilitation services compared with conventional care.	Function (WOMAC function scale)	No significant difference in mean 0-100 WOMAC pain scores (p=0.17):<br>Intervention: 23.5 (SD 22.3)<br>Control: 19.3 (SD 17.5)<br><br>Mean change from baseline (pre-surgery):

For peer review only

Appendix 3: Trial results summary

Study	Primary aim of the study	Primary outcome (and measure)	Results summary
			Intervention: 39.3<br>Control: 37.1
Ko et al, 2013	To determine whether center-based, one-to-one physical therapy provides superior outcomes compared with group-based therapy or a simple monitored home-based program.	Pain and function (Oxford Knee Score)	No significant difference in mean 0-50 WOMAC pain scores (p=0.79):<br>1:1 sessions: Median 3.8 (IQR 0.5-9.6)<br>Group sessions: Median 1.6 (IQR 0-7.5)<br>Home programme: Median 2.5 (IQR 0-9.5)<br><br>Mean change from baseline (pre-surgery):<br>1:1 sessions: 25.65<br>Group sessions: 18.4<br>Home programme: 25.7
Kramer et al, 2003	To compare clinic-based rehabilitation delivered in outpatient physical therapy clinics and home-based rehabilitation monitored by a physical therapist via periodic telephone calls.	Not specified	No significant difference in mean WOMAC pain scores (p-value and mean pain scores not reported)
Liebs et al, 2010	Evaluate the effect of ergometer cycling on health-related quality of life and patient satisfaction.	Function (WOMAC function scale)	No significant difference in mean 0-100 WOMAC pain scores (p=0.454):<br>Intervention: 15.6 (SD 17.9)<br>Control: 13.0 (SD 14.9)<br><br>Mean change from baseline (pre-surgery):<br>Intervention: 38.8<br>Control: 41.1
Liebs et al, 2012	To evaluate if the timing of aquatic therapy influences clinical outcomes.	Function (WOMAC function scale)	No significant difference in mean 0-100 WOMAC pain scores (p=0.334):<br>Intervention: 13.2 (SD 15.0)<br>Control: 17.4 (SD 22.4)

For peer review only

Appendix 3: Trial results summary

Study	Primary aim of the study	Primary outcome (and measure)	Results summary
			Mean change from baseline (pre-surgery):<br>Intervention: 39.9<br>Control: 32.8
Minns Lowe et al, 2012	To evaluate a pilot trial of a postdischarge physiotherapy intervention to improve patient function versus usual physiotherapy.	Pain and function (Oxford Knee Score)	No statistical comparison of median 0-100 KOOS pain scores (pilot study):<br>Intervention: 80.6 (IQ 36)<br>Control: 90.3 (IQ 33)<br><br>Mean change from baseline (pre-surgery):<br>Intervention: 39.5<br>Control: 51.4
Moffet et al, 2004	To evaluate the effectiveness of a new intensive functional rehabilitation program on functional ability and quality of life.	Walking distance (6 minute walk test)	No significant difference in mean 0-100 WOMAC pain scores (p=0.161):<br>Intervention: 9.4 (SD 12.4)<br>Control: 11.8 (SD 13.0)<br><br>Mean change from baseline (2 months post-operative):<br>Intervention: 19<br>Control: 10.8
Monticone et al, 2013	To compare the improvement in disability, kinesiophobia, pain, and quality of life obtained by means of home-based functional exercises aimed at managing kinesiophobia with advice to stay active after discharge from a rehabilitation unit.	Pain and function (KOOS)	Mean 0-100 KOOS pain score significantly lower in intervention group (p<0.001):<br>Intervention: 87.35 (SD 11.71)<br>Control: 77.38 (SD 15.07)<br><br>Mean change from baseline (before discharge from the rehabilitation unit):<br>Intervention: 41.95<br>Control: 34.64
Petterson et al, 2009	To determine the effectiveness of progressive quadriceps strengthening	Quadriceps strength and volitional	No significant difference in mean 0-5 KOS ADL item 'affect of pain on function' (p value not reported):

Appendix 3: Trial results summary

Study	Primary aim of the study	Primary outcome (and measure)	Results summary
	with or without neuromuscular electrical stimulation (NMES).	muscle activation (burst superimposition technique)	Intervention: 0.82 (SD not reported)<br>Control: 0.89 (SD not reported)<br><br>Mean change from baseline (3-4 weeks post-operative):<br>Intervention: 1.42<br>Control: 1.55
Szots et al, 2016	To evaluate the effects of structured nurse-managed telephone follow-up.	Function (WOMAC function scale)	No significant difference in mean change in 0-100 WOMAC pain scores from baseline (3 days post hospital discharge) (p=0.329):<br>Intervention: -25.9 (95% CI = -30.8, -21.0)<br>Control: -29.5 (95% CI = -35.2, -23.8)
Vuorenmaa et al, 2014	To evaluate the efficacy of a delayed home exercise programme compared with normal care.	Pain (WOMAC pain scale)	No significant difference in mean change in 0-100 WOMAC pain scores from baseline (2 months post-operative) (p=0.70):<br>Intervention: -15 (95% CI -20 to -10)<br>Control: -14 (95% CI -19 to -9)

For peer review only

Appendix 4: Adverse event reporting and findings

Publication	Adverse events assessment/definition	Results summary
Buker et al, 2014	None	
Bruun-Olsen et al, 2013	None	
Buhagiar et al, 2017	Postdischarge complication and adverse event data were collected until 1 year after surgery by self-report at follow-up visits and by a review of hospital electronic medical records.	Detailed breakdown of post-operative complications is provided in Table 3. There were no significant between-group differences in emergency department visits, readmissions and manipulations under anaesthetic.
Chen et al, 2016	None	
Fransen et al, 2017	Adverse events defined as event resulting in readmission to the hospital or resulting in a medical intervention or reduced function for 3 or more days.	Intervention: 1 death, 24 hospital admissions (15 TKR related), 20 other adverse events<br><br>Control: 1 death, 16 hospital admissions (13 TKR related), 17 other adverse events
Frost et al, 2002	None	
Kauppila et al, 2010	Not stated	No adverse events due to the intervention were reported
Ko et al, 2013	Postoperative adverse events were monitored via treating therapists and documented by the blinded assessor.	Detailed breakdown of post-operative adverse events provided in Appendix 1. Superficial wound infection, major infection, venous thrombotic embolism, neurovascular, TKR-related readmission and manipulation under anaesthetic similar between groups. No adverse events were associated with any of the treatment arms.
Kramer et al, 2003	None	
Liebs et al, 2010	Not stated	Prevalence of postoperative complications was similar between groups.
Liebs et al, 2012	Not stated	5 patients in early aquatic therapy group and 1 patient in the late aquatic therapy group admitted to hospital within 3 months of surgery.
Minns Lowe et al, 2012	Not stated	No adverse events
Moffet et al, 2004	Not stated	No adverse events
Monticone et al, 2013	None	
Petterson et al, 2009	Not stated	No adverse events were related to the

For peer review only - http://bmjopen.bmj.com/site/about/guidelines.xhtml

		exercise intervention. Only 1 patient reported feeling dizzy and lightheaded following the first NMES treatment.
Szots et al, 2016	None	
Vuorenmaa et al, 2014	Not stated	Intervention: 5 patients discontinued training due to pain (2 reported knee pain on operated side, 1 reported knee pain on the contralateral side, 1 reported back pain, and 1 reported hip pain), 1 patient had surgery due to reduced range of motion

For peer review only
